# Supplementary material for: Multiple E3 ligases control tankyrase stability and function
Source: Nat Commun. 2023 Nov 8;14:7208. doi: 10.1038/s41467-023-42939-3 (PMC10632493; doi:10.1038/s41467-023-42939-3)
Supplement: Supplementary file 1 — Supplementary Information [file 41467_2023_42939_MOESM1_ESM.pdf]

Supplementary Information

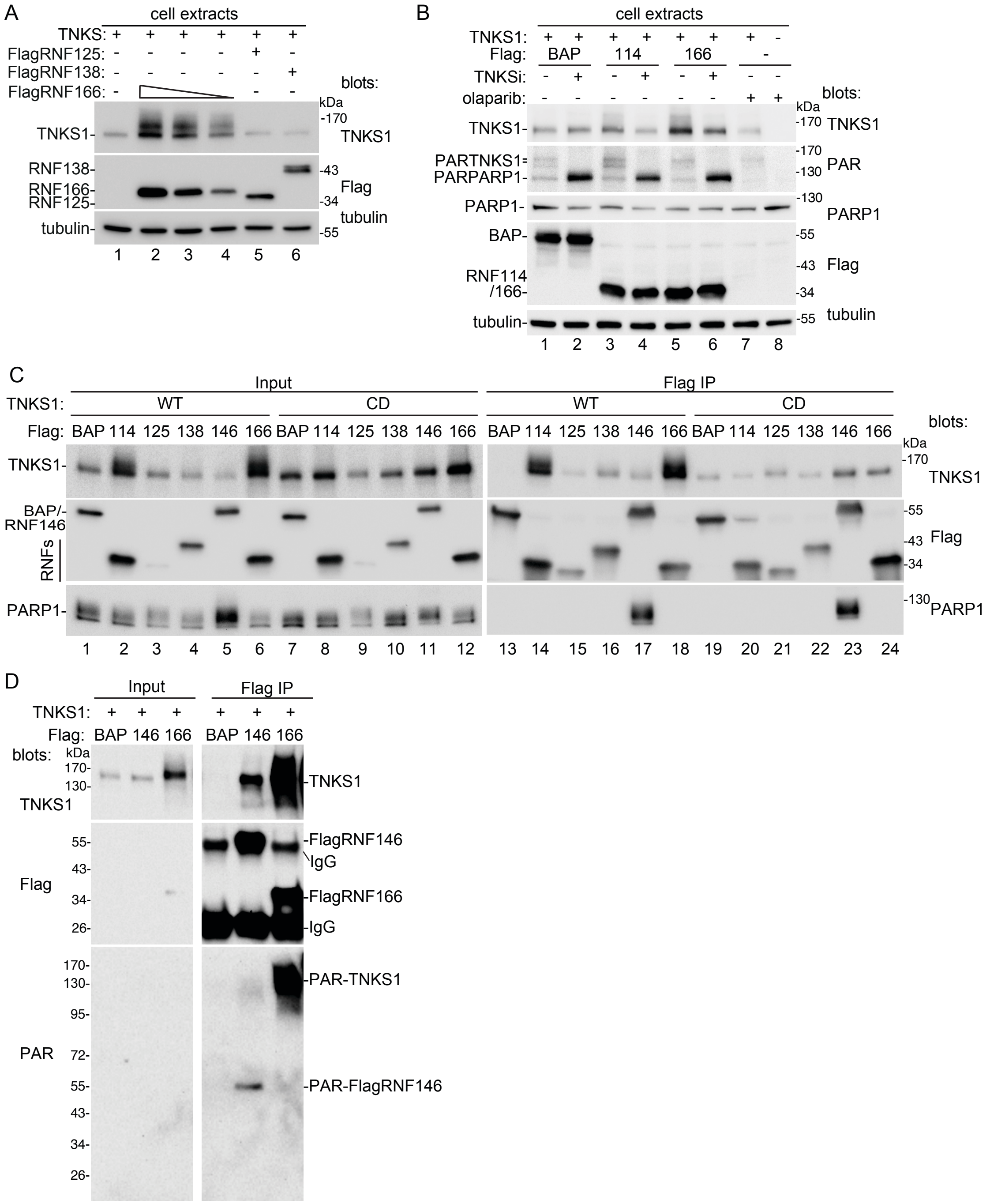

Supplementtray Fig.1. Analysis of RING-UIM E3 Ligases binding to tankyrase. Related to Fig.1 **A.** Immunoblot analysis of TNKS1/2 DKO HEK293T cells transfected with TNKS1 and the indicated Flag plasmids, and probed with the indicated antibodies. Two independent experiments produced similar results. **B.** Immunoblot analysis of TNKS1/2 DKO HEK293T cells transfected

with the indicated Flag plasmids and TNKS1, treated with and without TNKSi or olaparib, and probed with the indicated antibodies. At least three independent experiments produced similar results. **C.** Immunoblot analysis of TNKS1/2 DKO HEK293T cells transfected with TNKS1 WT or catalytically dead (CD) and the indicated Flag plasmids, immunoprecipitated with anti-Flag antibody, and probed with the indicated antibodies. Two independent experiments produced similar results. **D.** Immunoblot analysis of PARP1$^{-/-}$/PARP2$^{-/-}$ hTERT RPE-1 cells transfected with TNKS1 and the indicated Flag plasmids, immunoprecipitated with anti-Flag antibody, and probed with the indicated antibodies. Two independent experiments produced similar results. Source data are provided as a Source Data file.

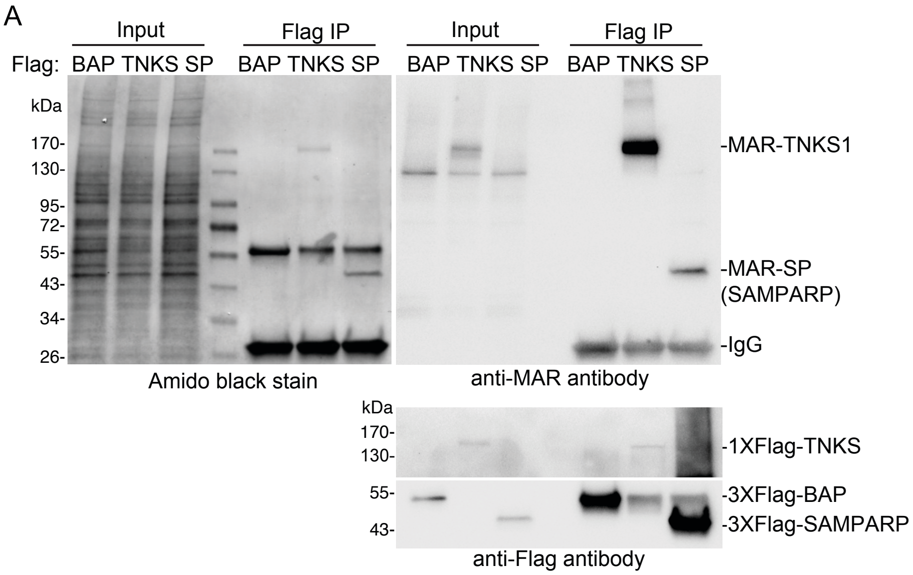

Supplementary Fig. 2. TNKS1 and SAMPARP are detected by anti-MAR antibody. Related to Fig. 2
A. Immunoblot analysis of TNKS1/2 DKO HEK293T cells transfected with indicated Flag plasmids, immunoprecipitated with anti-Flag antibody, and probed with the indicated antibodies or stained with amido black. Two independent experiments produced similar results. Source data are provided as a Source Data file.

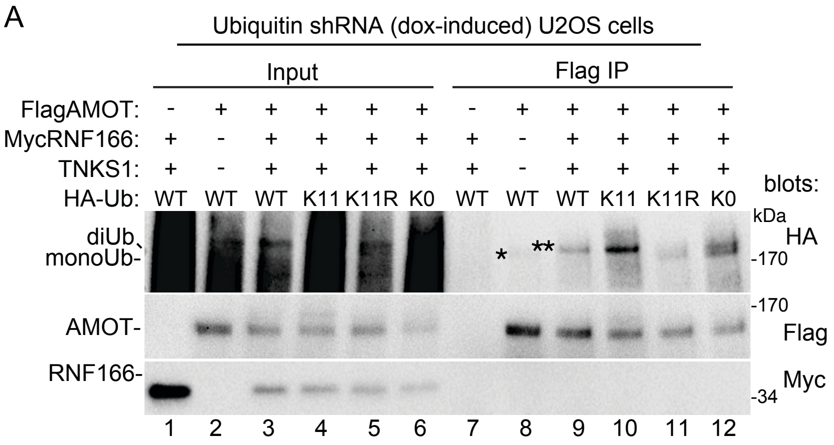

Supplementary Fig. 3. Analysis of AMOT ubiquitylation in ubiquitin shRNA cells. Related to Fig. 6. **A.** Immunoblot analysis of Ubiquitin shRNA U2OS cells treated with dox for 48 hr and transfected with FlagAMOT, MycRNF166, TNKS1, and HA-Ub plasmids, immunoprecipitated with anti-Flag antibody, and probed with the indicated antibodies. Two independent experiments produced similar results. (*) indicates monoUb; (**) indicates diUb. Source data are provided as a Source Data file.
