# Supplementary material for: Multiple E3 ligases control tankyrase stability and function
Source: Nat Commun. 2023 Nov 8;14:7208. doi: 10.1038/s41467-023-42939-3 (PMC10632493; doi:10.1038/s41467-023-42939-3)
Supplement: Supplementary file 3 — Description of Additional Supplementary Files [file 41467_2023_42939_MOESM3_ESM.pdf]

Description of Additional Supplementary Files

File name: Supplementary Data 1
Description: List of proteins and peptides obtained by mass spectrometry analysis of FlagRNF166 immunoprecipitates.

File name: Supplementary Data 2:
Description: List of plasmids, primers, and antibodies used in the paper.
