# Supplementary material for: Multiple E3 ligases control tankyrase stability and function
Source: Nat Commun. 2023 Nov 8;14:7208. doi: 10.1038/s41467-023-42939-3 (PMC10632493; doi:10.1038/s41467-023-42939-3)
Supplement: Supplementary file 6 — Reporting Summary [file 41467_2023_42939_MOESM6_ESM.pdf]

nature portfolio

Corresponding author(s): Susan Smith

Last updated by author(s): 10/19/2023

Reporting Summary

Nature Portfolio wishes to improve the reproducibility of the work that we publish. This form provides structure for consistency and transparency in reporting. For further information on Nature Portfolio policies, see our Editorial Policies and the Editorial Policy Checklist.

Statistics

For all statistical analyses, confirm that the following items are present in the figure legend, table legend, main text, or Methods section.

n/a	Confirmed	
☐	☒	The exact sample size (*n*) for each experimental group/condition, given as a discrete number and unit of measurement
☐	☒	A statement on whether measurements were taken from distinct samples or whether the same sample was measured repeatedly
☐	☒	The statistical test(s) used AND whether they are one- or two-sided<br>*Only common tests should be described solely by name; describe more complex techniques in the Methods section.*
☒	☐	A description of all covariates tested
☒	☐	A description of any assumptions or corrections, such as tests of normality and adjustment for multiple comparisons
☐	☒	A full description of the statistical parameters including central tendency (e.g. means) or other basic estimates (e.g. regression coefficient) AND variation (e.g. standard deviation) or associated estimates of uncertainty (e.g. confidence intervals)
☐	☒	For null hypothesis testing, the test statistic (e.g. *F*, *t*, *r*) with confidence intervals, effect sizes, degrees of freedom and *P* value noted<br>*Give P values as exact values whenever suitable.*
☒	☐	For Bayesian analysis, information on the choice of priors and Markov chain Monte Carlo settings
☒	☐	For hierarchical and complex designs, identification of the appropriate level for tests and full reporting of outcomes
☒	☐	Estimates of effect sizes (e.g. Cohen's *d*, Pearson's *r*), indicating how they were calculated

Our web collection on statistics for biologists contains articles on many of the points above.

Software and code

Policy information about availability of computer code

Data collection	For western blotting, acquisition and analysis was performed using Image Lab 6.0.1.<br>MS/MS spectra were searched against a Uniprot human database using Sequest within Proteome Discoverer 2.5.
Data analysis	Statistical analysis was performed using Prism 9 software. For western blotting, acquisition and analysis was performed using Image Lab 6.0.1.<br>MS/MS spectra were searched against a Uniprot human database using Sequest within Proteome Discoverer 2.5.

For manuscripts utilizing custom algorithms or software that are central to the research but not yet described in published literature, software must be made available to editors and reviewers. We strongly encourage code deposition in a community repository (e.g. GitHub). See the Nature Portfolio guidelines for submitting code & software for further information.

Data

Policy information about availability of data

All manuscripts must include a data availability statement. This statement should provide the following information, where applicable:

- Accession codes, unique identifiers, or web links for publicly available datasets
- A description of any restrictions on data availability
- For clinical datasets or third party data, please ensure that the statement adheres to our policy

The mass spec data is in the following repository: MassIVE (https://massive.ucsd.edu/): accession number MSV000092070
Reviewer log in:

Username: MSV000092070_reviewer
Password: mass_spec_raw
The mass spec data is also available in the following repository: ProteomeXchange (https://www.proteomexchange.org/): PXD042595 (use MassIVE access credential).
The data that support the findings of this study are included in this published article and available from the corresponding author upon reasonable request.

Research involving human participants, their data, or biological material

Policy information about studies with human participants or human data. See also policy information about sex, gender (identity/presentation), and sexual orientation and race, ethnicity and racism.

Reporting on sex and gender	N/A
Reporting on race, ethnicity, or other socially relevant groupings	N/A
Population characteristics	N/A
Recruitment	N/A
Ethics oversight	N/A

Note that full information on the approval of the study protocol must also be provided in the manuscript.

Field-specific reporting

Please select the one below that is the best fit for your research. If you are not sure, read the appropriate sections before making your selection.

☒ Life sciences ☐ Behavioural & social sciences ☐ Ecological, evolutionary & environmental sciences

For a reference copy of the document with all sections, see nature.com/documents/nr-reporting-summary-flat.pdf

Life sciences study design

All studies must disclose on these points even when the disclosure is negative.

Sample size	No sample-size calculation was performed. Sample sizes were based upon experience and similar published studies.
Data exclusions	No data were excluded from the analysis.
Replication	The number of replications for each experiment are indicated. Experiments were performed successfully at least two to three times independently.
Randomization	No randomization was performed. Based on similar published work, this is not relevant for our experiments.
Blinding	Blinding was not done. Based on similar published work, this is not relevant for our experiments.

Reporting for specific materials, systems and methods

We require information from authors about some types of materials, experimental systems and methods used in many studies. Here, indicate whether each material, system or method listed is relevant to your study. If you are not sure if a list item applies to your research, read the appropriate section before selecting a response.

Materials & experimental systems

n/a	Involved in the study	
☐	☒	Antibodies
☐	☒	Eukaryotic cell lines
☒	☐	Palaeontology and archaeology
☒	☐	Animals and other organisms
☒	☐	Clinical data
☒	☐	Dual use research of concern
☒	☐	Plants

Methods

n/a	Involved in the study	
☒	☐	ChIP-seq
☒	☐	Flow cytometry
☒	☐	MRI-based neuroimaging

Antibodies

Antibodies used	α-TNKS1 Smith Lab 762 762 Rabbit 1/2500; α-TNKS1 Smith Lab 763 763 Rabbit 1/1000; α-Flag Sigma F3165 M2 Mouse 1/5000; α-Tubulin Sigma T5168 B-5-1-2 Mouse 1/10000; α-Mouse-HRP GE Healthcare NA931V Polyclo. Donkey 1/2500; α-Rabbit-HRP GE Healthcare NA934V Polyclo. Donkey 1/2500; α-Myc Millipore 05-724 4A6 Mouse 1/5000; PARP1 BD Pharmingen 556493 7D3-6 Mouse 1/1000; α-O/PAR Millipore MABE1031 not an Ab Rabbit 1/5000; α-RNF146 Invitrogen PA5-55544 Polyclo. Rabbit 1/1000; α-HA (tag) Abcam ab9110 Polyclo. Rabbit 1/5000; α-RNF114 Sigma HPA021184 Polyclo. Rabbit 1/1000; α-AMOT Bethyl A303-305A-T Polyclo. Rabbit 1/2000; α-RNF166 Fisher PA5-111169 Polyclo. Rabbit 1/200; α-K11-Ubiquitin Invitrogen PA5-120621 Polyclo. Rabbit 1/1000; IgG CTRL Cell Signaling Technology 2729S Polyclo. Rabbit; α-TNKS1 Smith Lab 465 465 Rabbit.
Validation	Antibodies were validated by the manufacturers, in previous studies, or in western blots using overexpression or depletion. All the primary antibodies were validated for human species and used on human cell lines.

Eukaryotic cell lines

Policy information about cell lines and Sex and Gender in Research

Cell line source(s)	HEK293T (ATCC), U20S (ATCC), TNKS1/2 DKO(Bhardwaj et al., 2017), HEK293A WT and RNF146 KO(Nie et al, 2020) (provided by Dr. Junjie Chen), Super HeLa(Cristofari & Lingner, 2006) (provided by Dr. Joachim Lingner), hTERT RPE-1 PARP1-/-/ PARP2-/- cells(Hazlinkova et al. 2017) (provided by Dr. Keith Caldecottand), and U2OS shUb(Shearer et al., 2022) (provided by Dr. Niels Mailand).
Authentication	The cell lines were not authenticated.
Mycoplasma contamination	The cell lines were not tested for mycoplasma contamination.
Commonly misidentified lines (See ICLAC register)	None used.
