# Supplementary material for: Multiple E3 ligases control tankyrase stability and function
Source: Nat Commun. 2023 Nov 8;14:7208. doi: 10.1038/s41467-023-42939-3 (PMC10632493; doi:10.1038/s41467-023-42939-3)
Supplement: Supplementary file 7 — Source Data [file 41467_2023_42939_MOESM7_ESM.zip › Source_data_western_blots.pdf]

1b

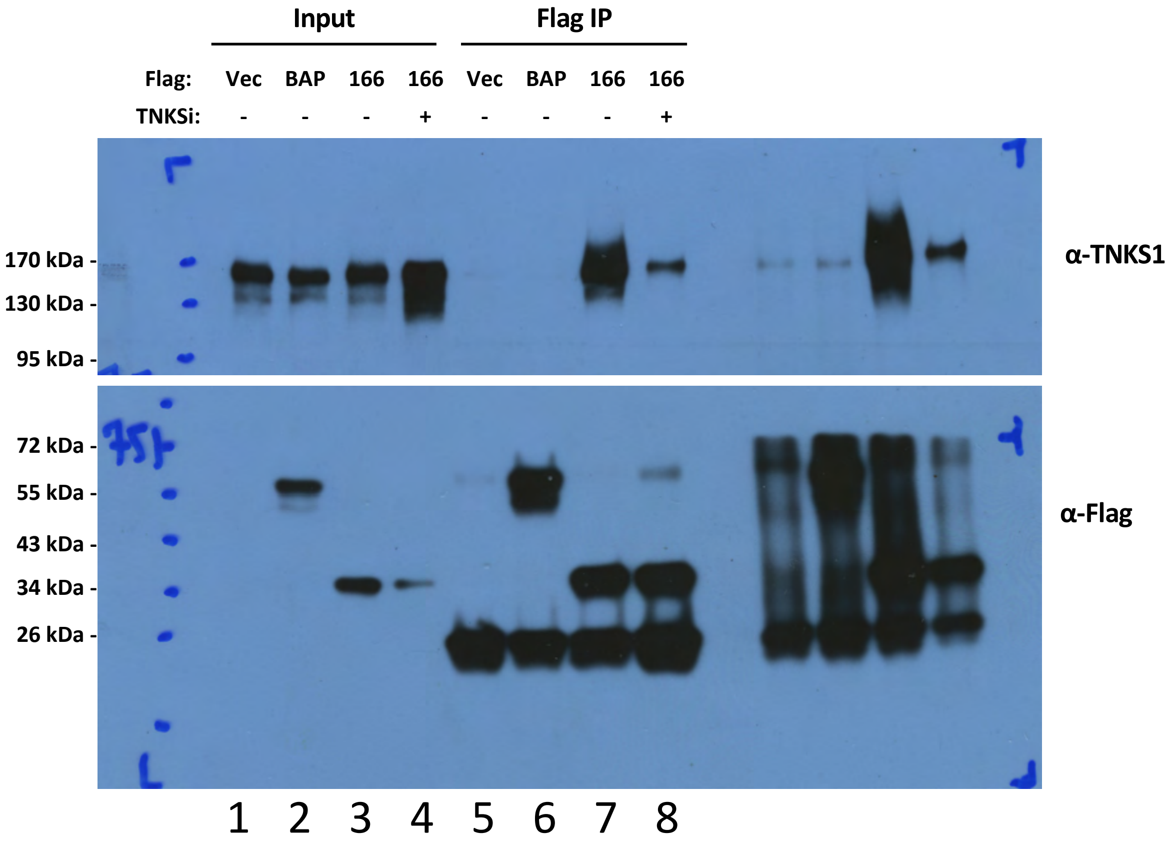

Input
Flag IP
Flag: Vec BAP 166 166 Vec BAP 166 166
TNKSi: - - - + - - - +
170 kDa
130 kDa
95 kDa
72 kDa
55 kDa
43 kDa
34 kDa
26 kDa
α-TNKS1
α-Flag
1 2 3 4 5 6 7 8

1c

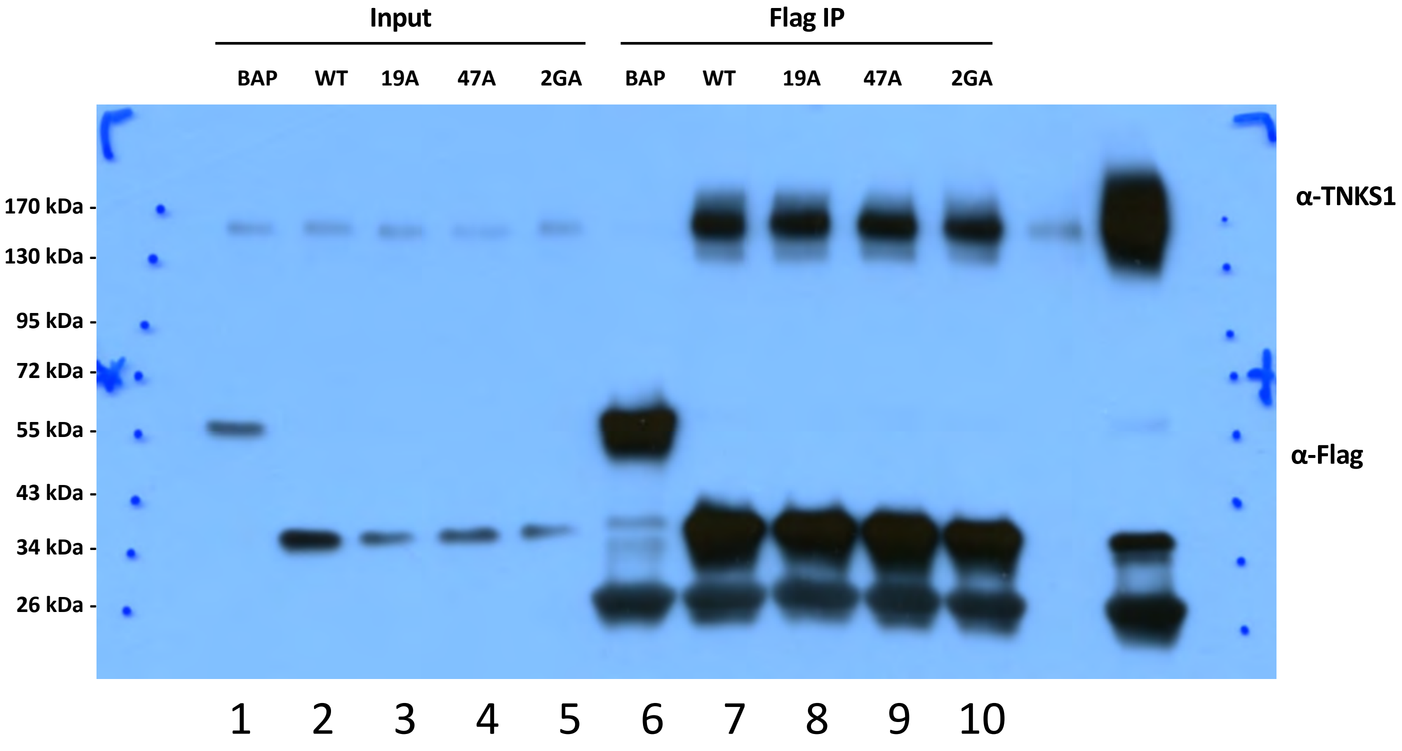

Input
Flag IP
BAP WT 19A 47A 2GA BAP WT 19A 47A 2GA
170 kDa
130 kDa
95 kDa
72 kDa
55 kDa
43 kDa
34 kDa
26 kDa
α-TNKS1
α-Flag
1 2 3 4 5 6 7 8 9 10

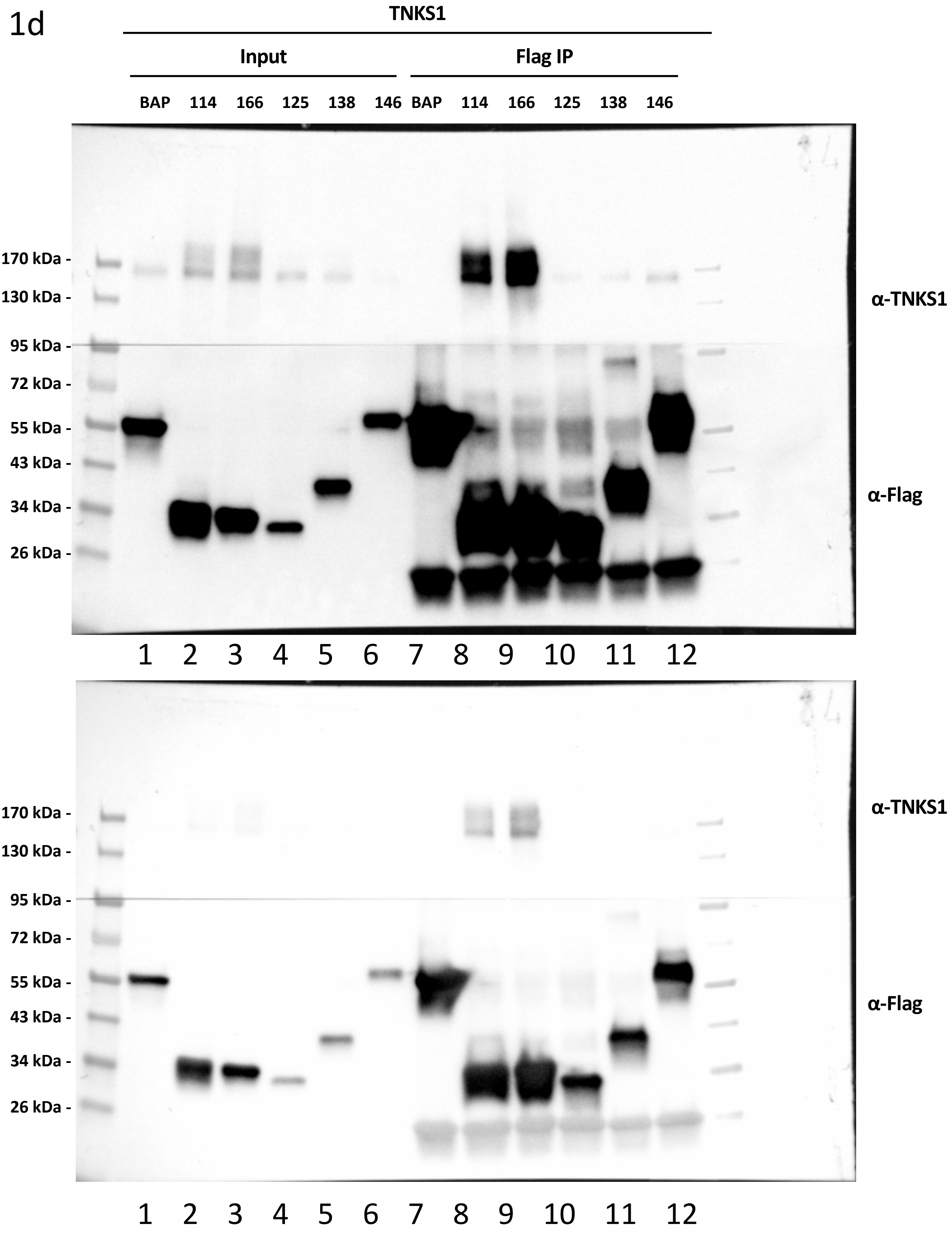
1d
TNKS1
Input
Flag IP
BAP
114
166
125
138
146
BAP
114
166
125
138
146
170 kDa -
130 kDa -
95 kDa -
72 kDa -
55 kDa -
43 kDa -
34 kDa -
26 kDa -
α-TNKS1
α-Flag
1
2
3
4
5
6
7
8
9
10
11
12
170 kDa -
130 kDa -
95 kDa -
72 kDa -
55 kDa -
43 kDa -
34 kDa -
26 kDa -
α-TNKS1
α-Flag
1
2
3
4
5
6
7
8
9
10
11
12

1e

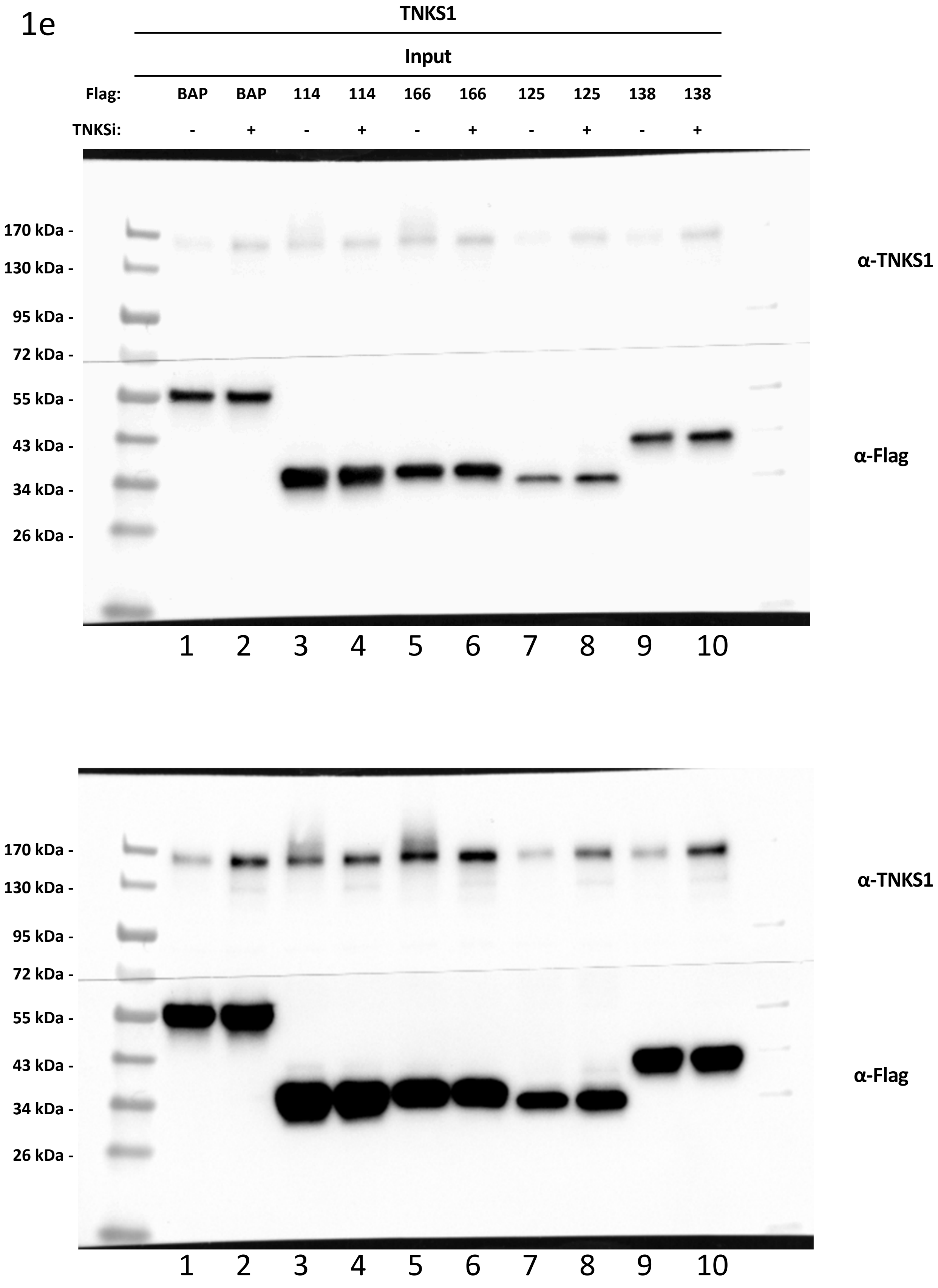
TNKS1
Input
Flag: BAP BAP 114 114 166 166 125 125 138 138
TNKSi: - + - + - + - + - +
170 kDa -
130 kDa -
95 kDa -
72 kDa -
55 kDa -
43 kDa -
34 kDa -
26 kDa -
α-TNKS1
α-Flag
1 2 3 4 5 6 7 8 9 10
170 kDa -
130 kDa -
95 kDa -
72 kDa -
55 kDa -
43 kDa -
34 kDa -
26 kDa -
α-TNKS1
α-Flag
1 2 3 4 5 6 7 8 9 10

1e

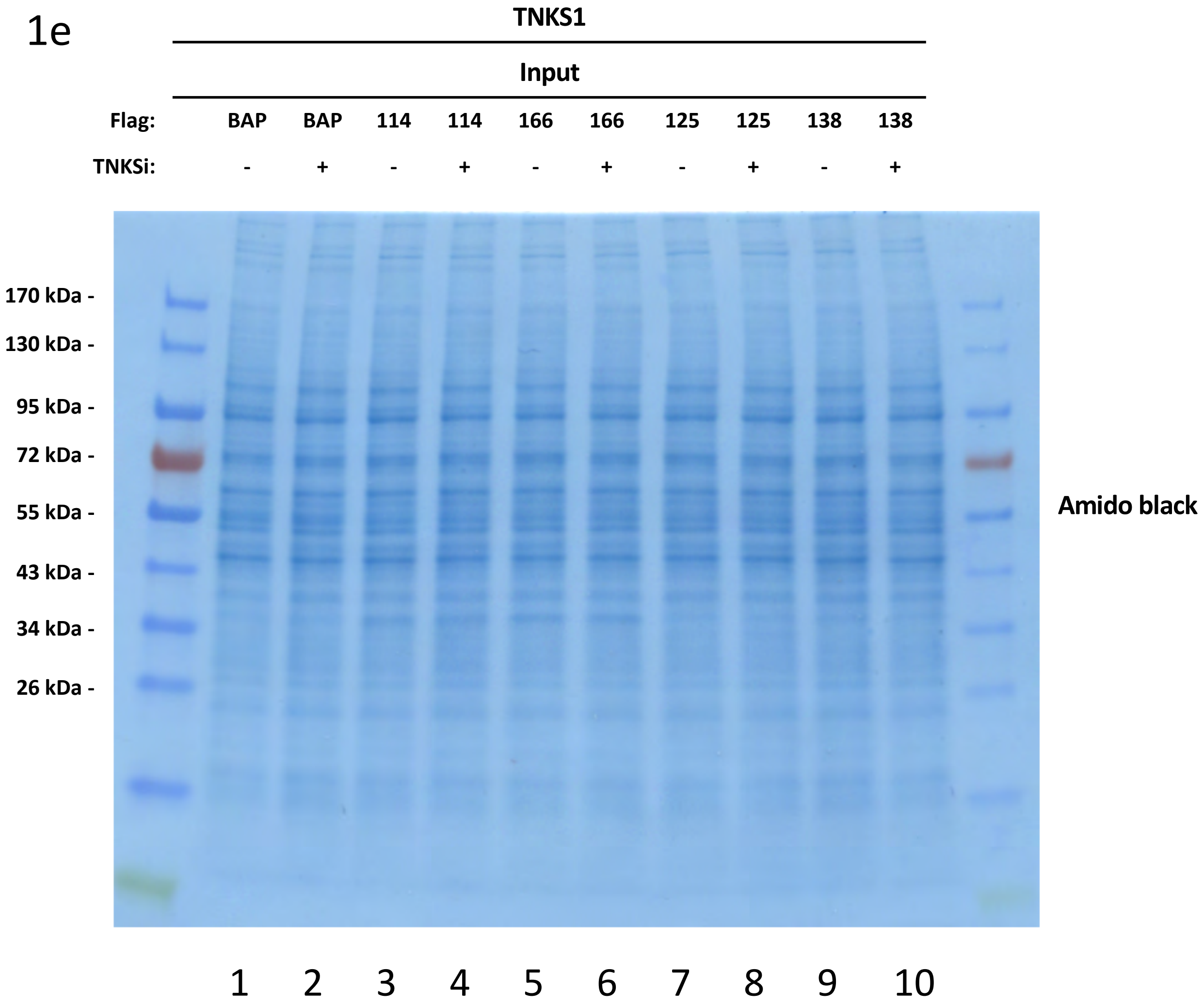
TNKS1
Input
Flag: BAP BAP 114 114 166 166 125 125 138 138
TNKSi: - + - + - + - + - +
170 kDa -
130 kDa -
95 kDa -
72 kDa -
55 kDa -
43 kDa -
34 kDa -
26 kDa -
Amido black
1 2 3 4 5 6 7 8 9 10

1e

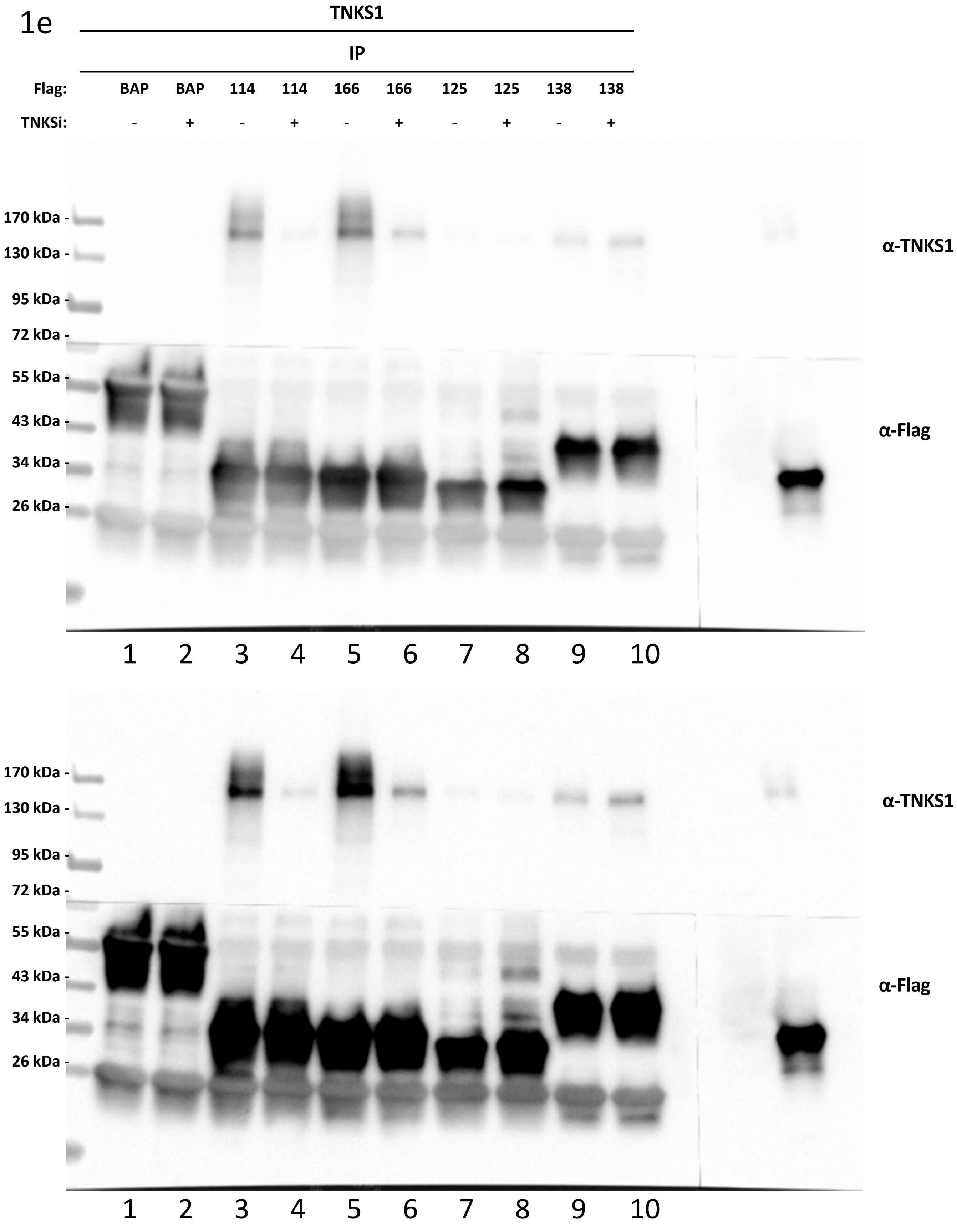
TNKS1
IP
Flag: BAP BAP 114 114 166 166 125 125 138 138
TNKSi: - + - + - + - + - +
170 kDa
130 kDa
95 kDa
72 kDa
55 kDa
43 kDa
34 kDa
26 kDa
α-TNKS1
α-Flag
1 2 3 4 5 6 7 8 9 10
170 kDa
130 kDa
95 kDa
72 kDa
55 kDa
43 kDa
34 kDa
26 kDa
α-TNKS1
α-Flag
1 2 3 4 5 6 7 8 9 10

1e

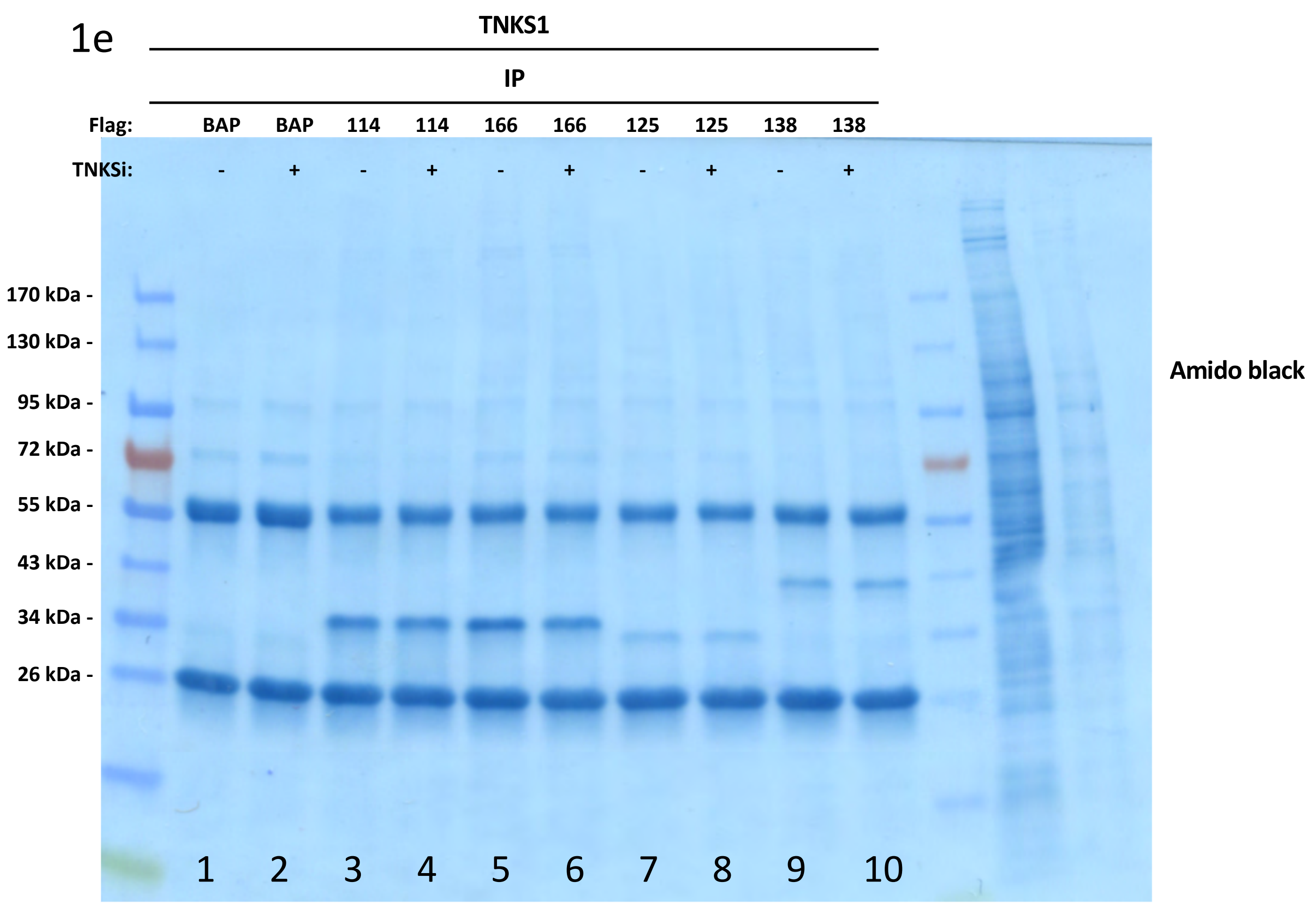
TNKS1
IP
Flag: BAP BAP 114 114 166 166 125 125 138 138
TNKSi: - + - + - + - + - +
170 kDa -
130 kDa -
95 kDa -
72 kDa -
55 kDa -
43 kDa -
34 kDa -
26 kDa -
1 2 3 4 5 6 7 8 9 10
Amido black

1f

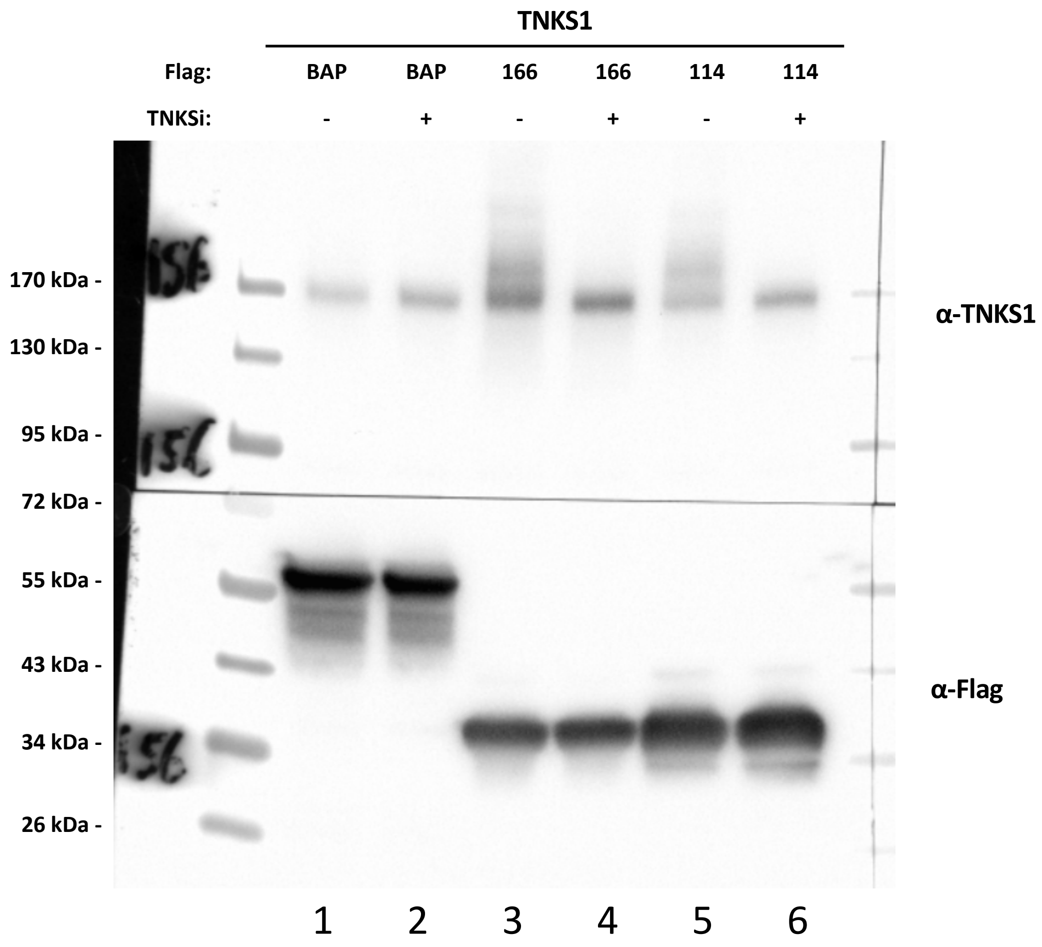

TNKS1
Flag: BAP BAP 166 166 114 114
TNKSi: - + - + - +
170 kDa -
130 kDa -
95 kDa -
72 kDa -
55 kDa -
43 kDa -
34 kDa -
26 kDa -
α-TNKS1
α-Flag
1 2 3 4 5 6

1f

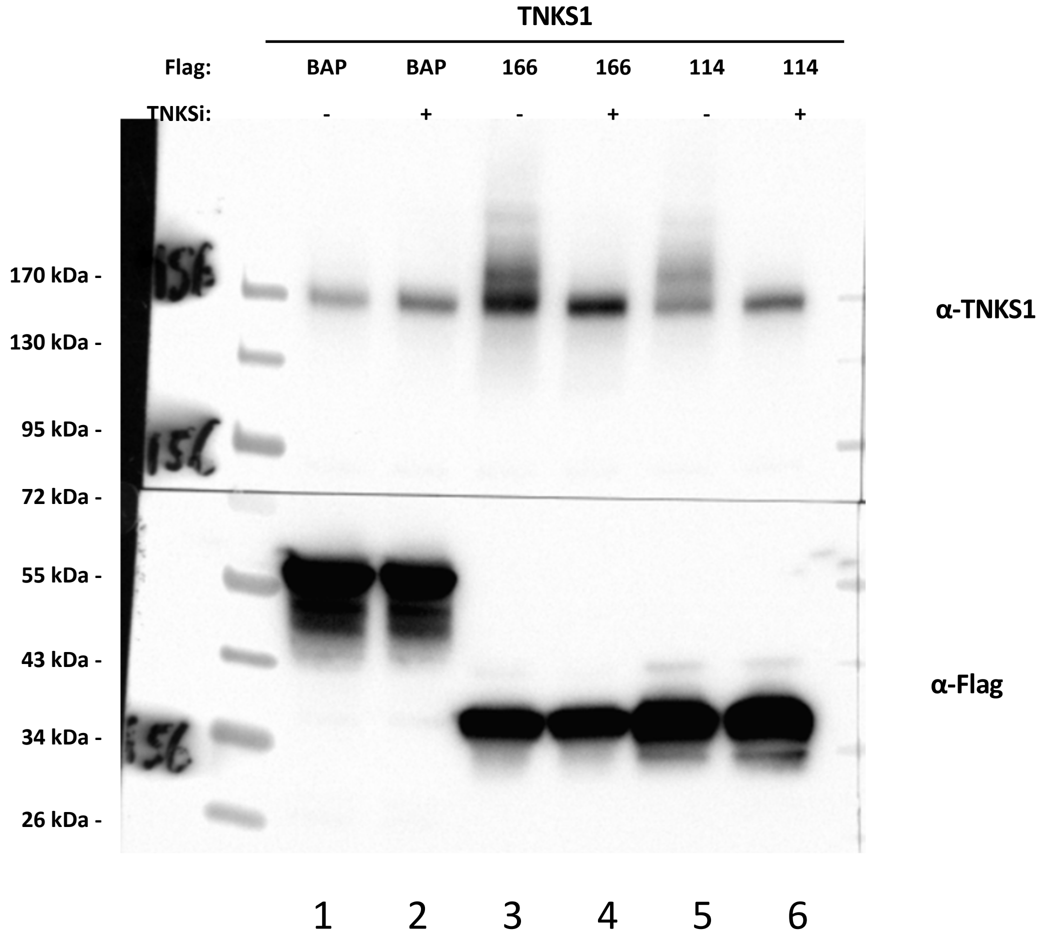
TNKS1
Flag: BAP BAP 166 166 114 114
TNKSi: - + - + - +
170 kDa -
130 kDa -
95 kDa -
72 kDa -
55 kDa -
43 kDa -
34 kDa -
26 kDa -
α-TNKS1
α-Flag
1 2 3 4 5 6

1f

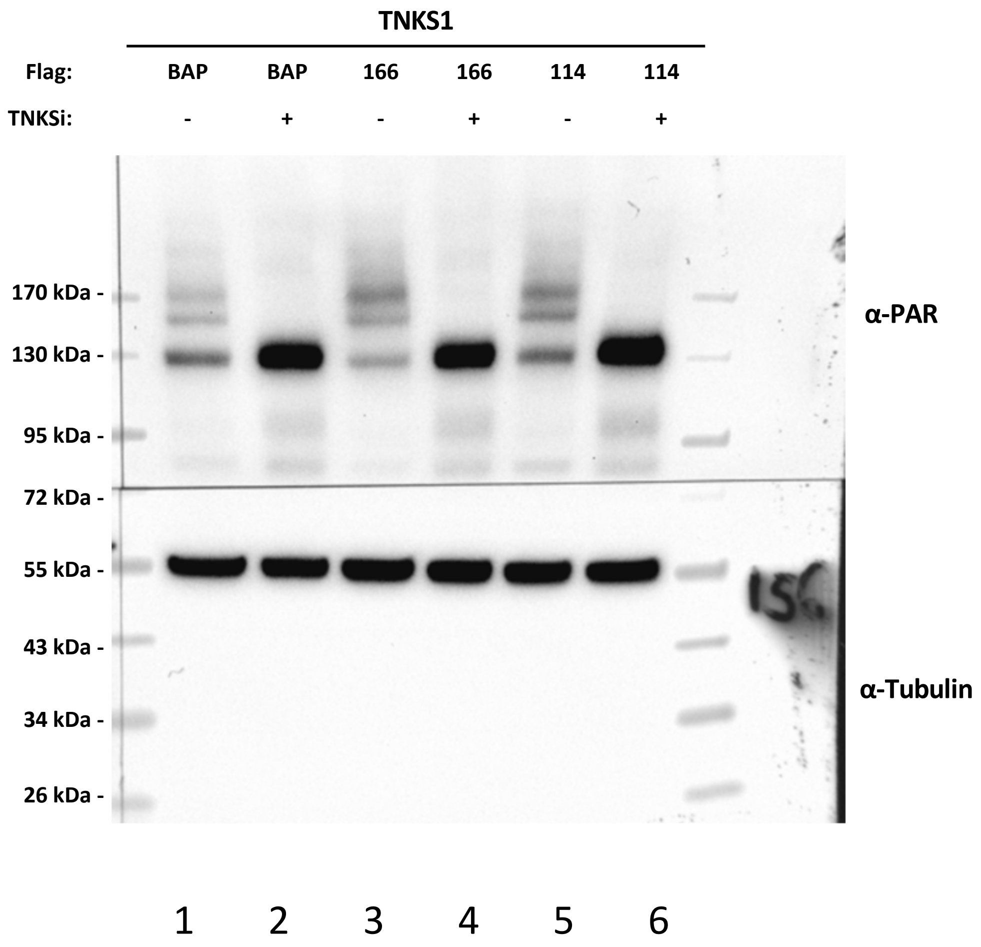
TNKS1
Flag: BAP BAP 166 166 114 114
TNKSi: - + - + - +
170 kDa -
130 kDa -
95 kDa -
72 kDa -
55 kDa -
43 kDa -
34 kDa -
26 kDa -
α-PAR
α-Tubulin
1 2 3 4 5 6

1i

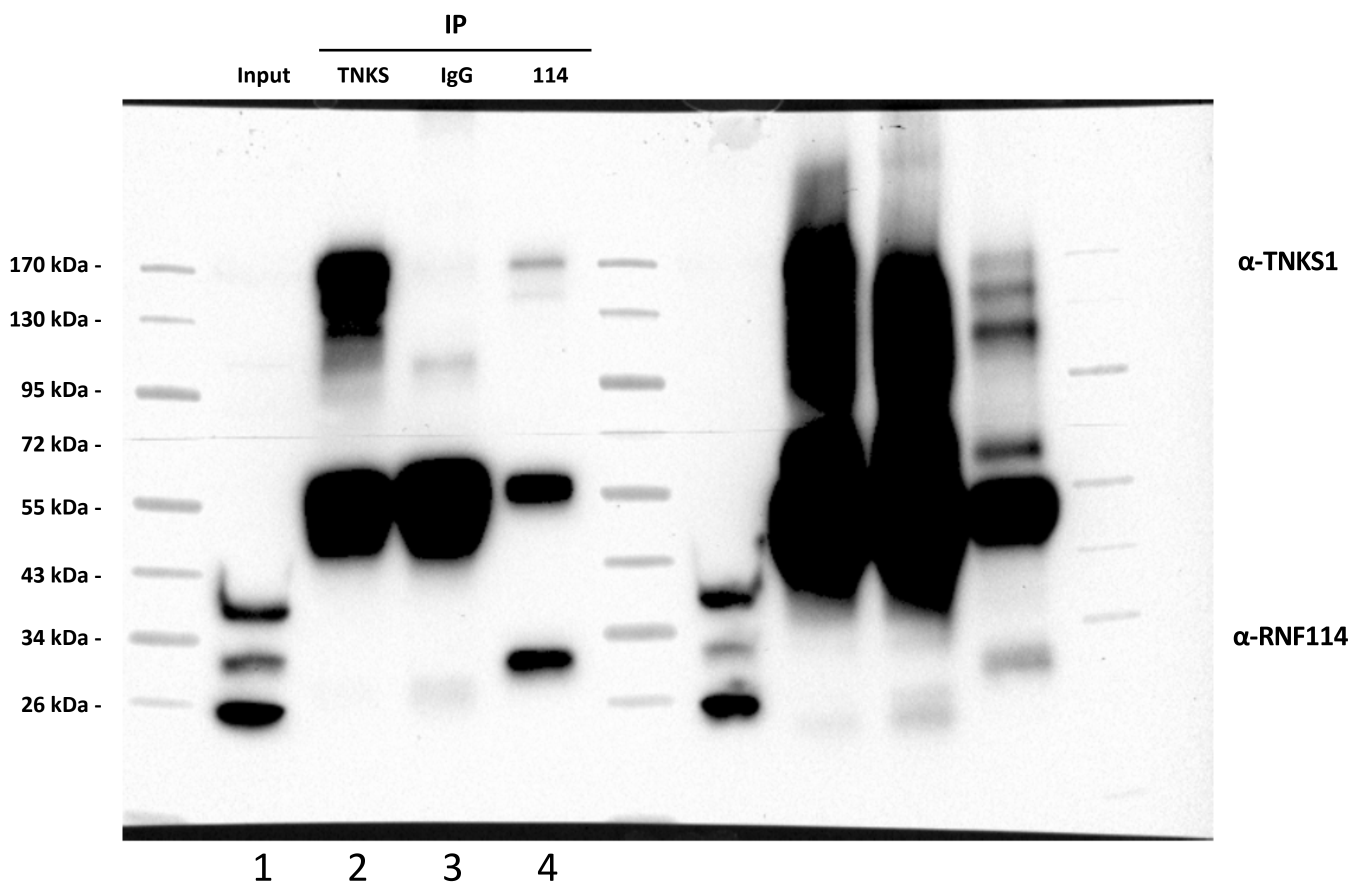
IP
Input
TNKS
IgG
114
170 kDa -
130 kDa -
95 kDa -
72 kDa -
55 kDa -
43 kDa -
34 kDa -
26 kDa -
α-TNKS1
α-RNF114
1
2
3
4

1i

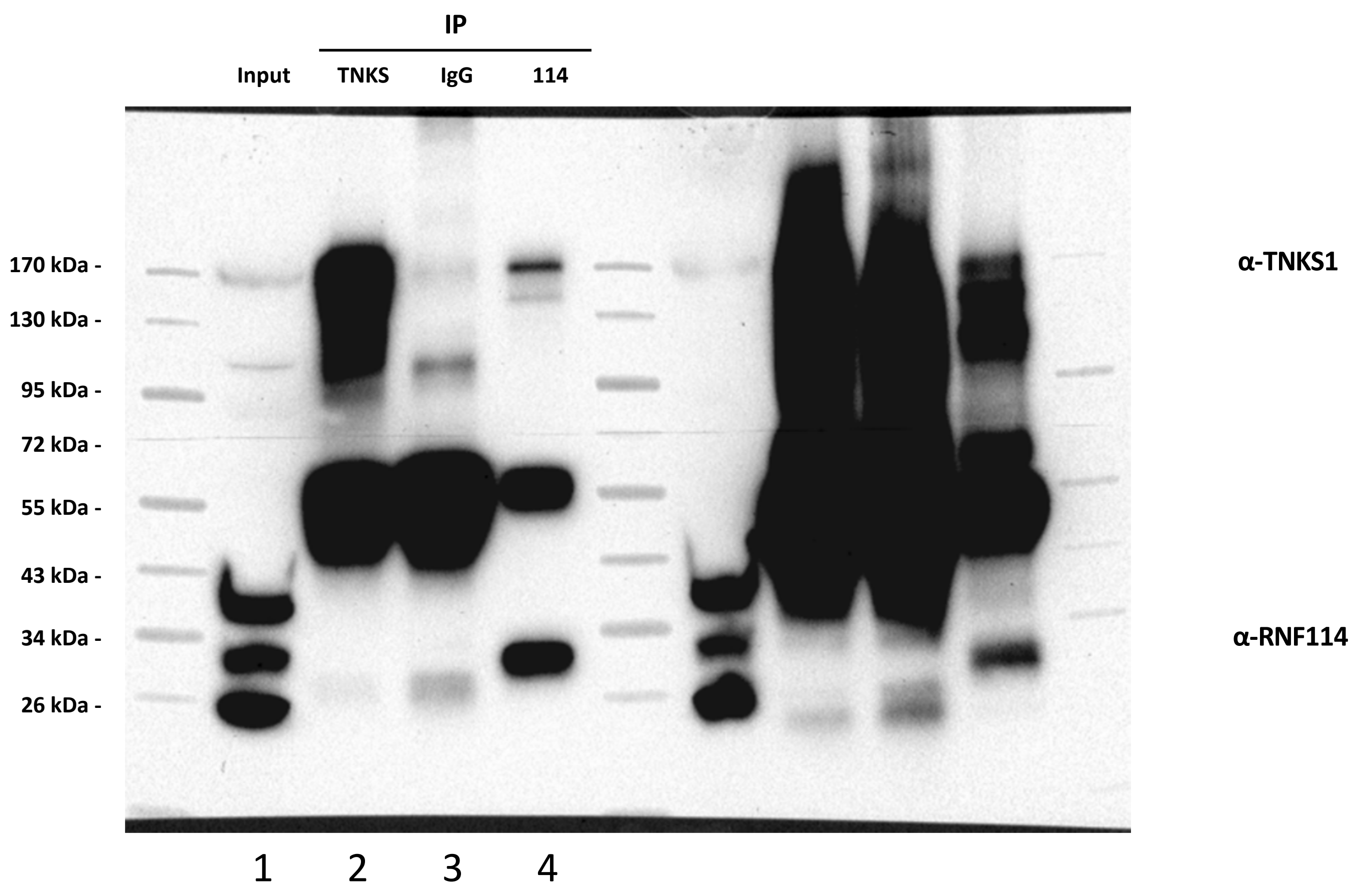
IP
Input
TNKS
IgG
114
170 kDa -
130 kDa -
95 kDa -
72 kDa -
55 kDa -
43 kDa -
34 kDa -
26 kDa -
α-TNKS1
α-RNF114
1
2
3
4

2b

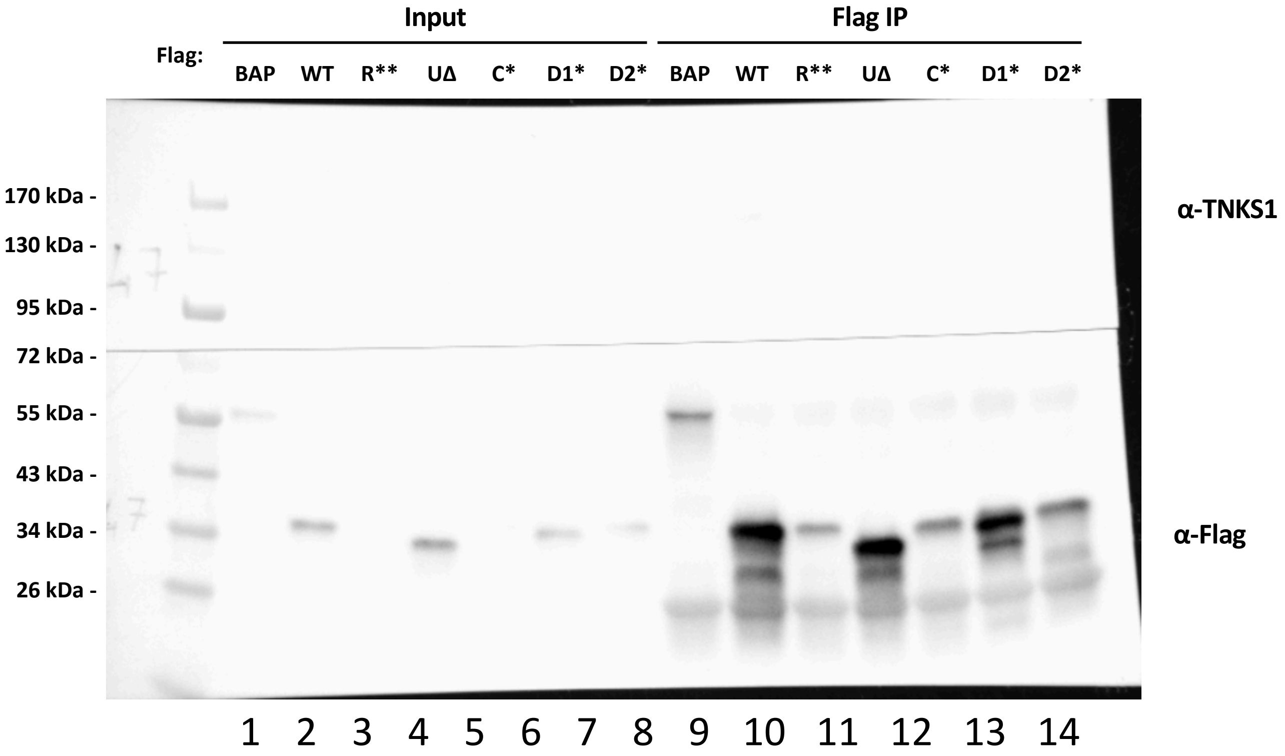
Input
Flag IP
Flag:
BAP WT R** UΔ C* D1* D2* BAP WT R** UΔ C* D1* D2*
170 kDa -
130 kDa -
95 kDa -
72 kDa -
55 kDa -
43 kDa -
34 kDa -
26 kDa -
α-TNKS1
α-Flag
1 2 3 4 5 6 7 8 9 10 11 12 13 14

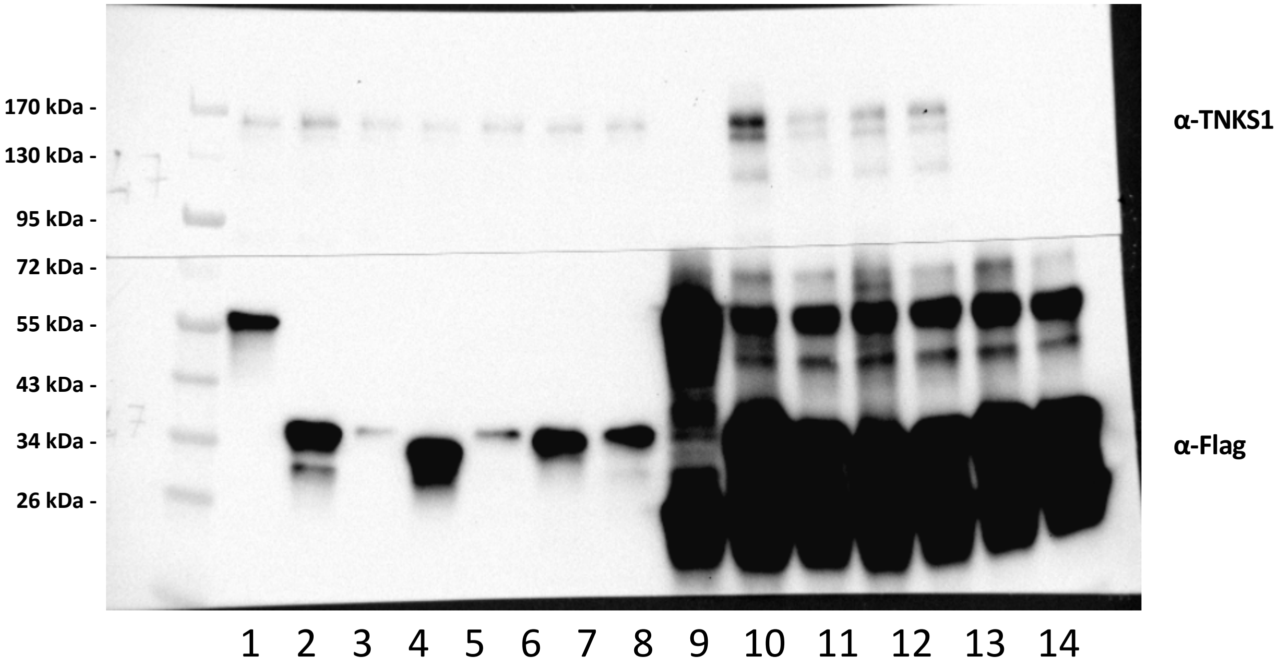
170 kDa -
130 kDa -
95 kDa -
72 kDa -
55 kDa -
43 kDa -
34 kDa -
26 kDa -
α-TNKS1
α-Flag
1 2 3 4 5 6 7 8 9 10 11 12 13 14

2c

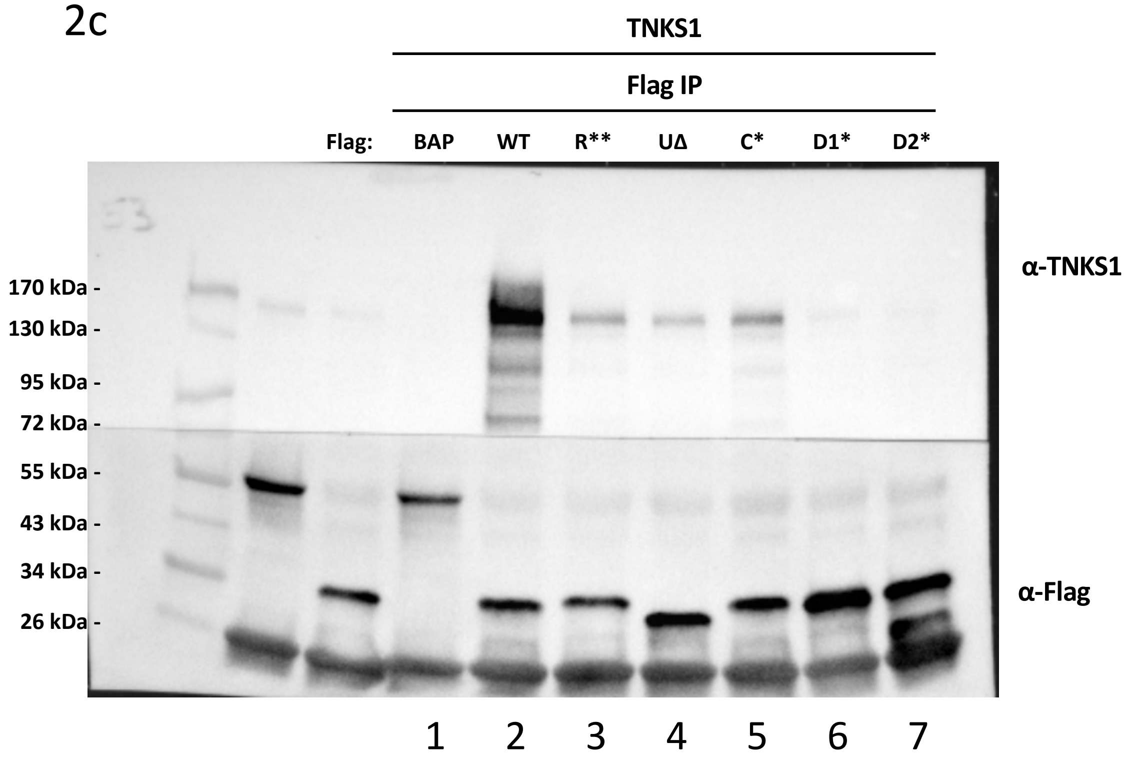
TNKS1
Flag IP
Flag:
BAP
WT
R**
UΔ
C*
D1*
D2*
170 kDa -
130 kDa -
95 kDa -
72 kDa -
55 kDa -
43 kDa -
34 kDa -
26 kDa -
α-TNKS1
α-Flag
1
2
3
4
5
6
7

2c

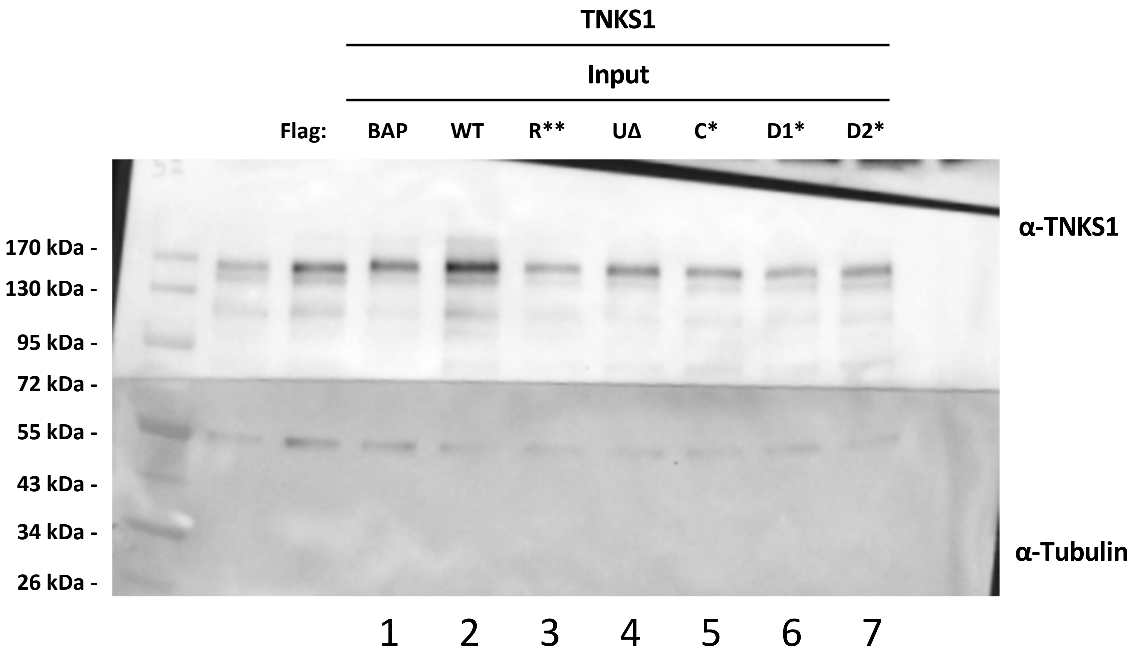
TNKS1
Input
Flag:
BAP
WT
R**
UΔ
C*
D1*
D2*
170 kDa -
130 kDa -
95 kDa -
72 kDa -
55 kDa -
43 kDa -
34 kDa -
26 kDa -
α-TNKS1
α-Tubulin
1
2
3
4
5
6
7

2e

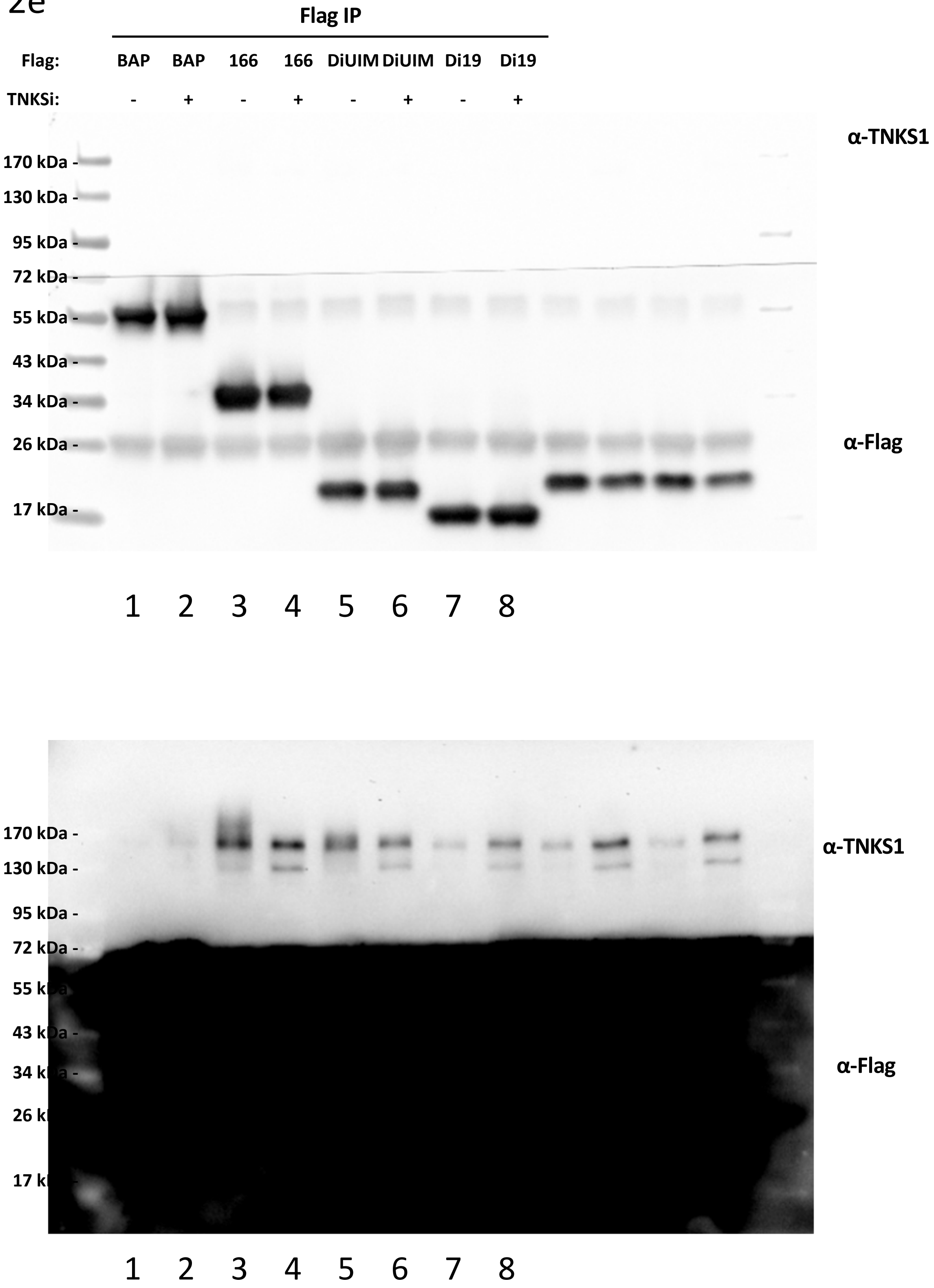

Flag IP
Flag: BAP BAP 166 166 DiUIM DiUIM Di19 Di19
TNKSi: - + - + - + - +
170 kDa -
130 kDa -
95 kDa -
72 kDa -
55 kDa -
43 kDa -
34 kDa -
26 kDa -
17 kDa -
α-TNKS1
α-Flag
1 2 3 4 5 6 7 8
170 kDa -
130 kDa -
95 kDa -
72 kDa -
α-TNKS1
α-Flag
1 2 3 4 5 6 7 8

2e

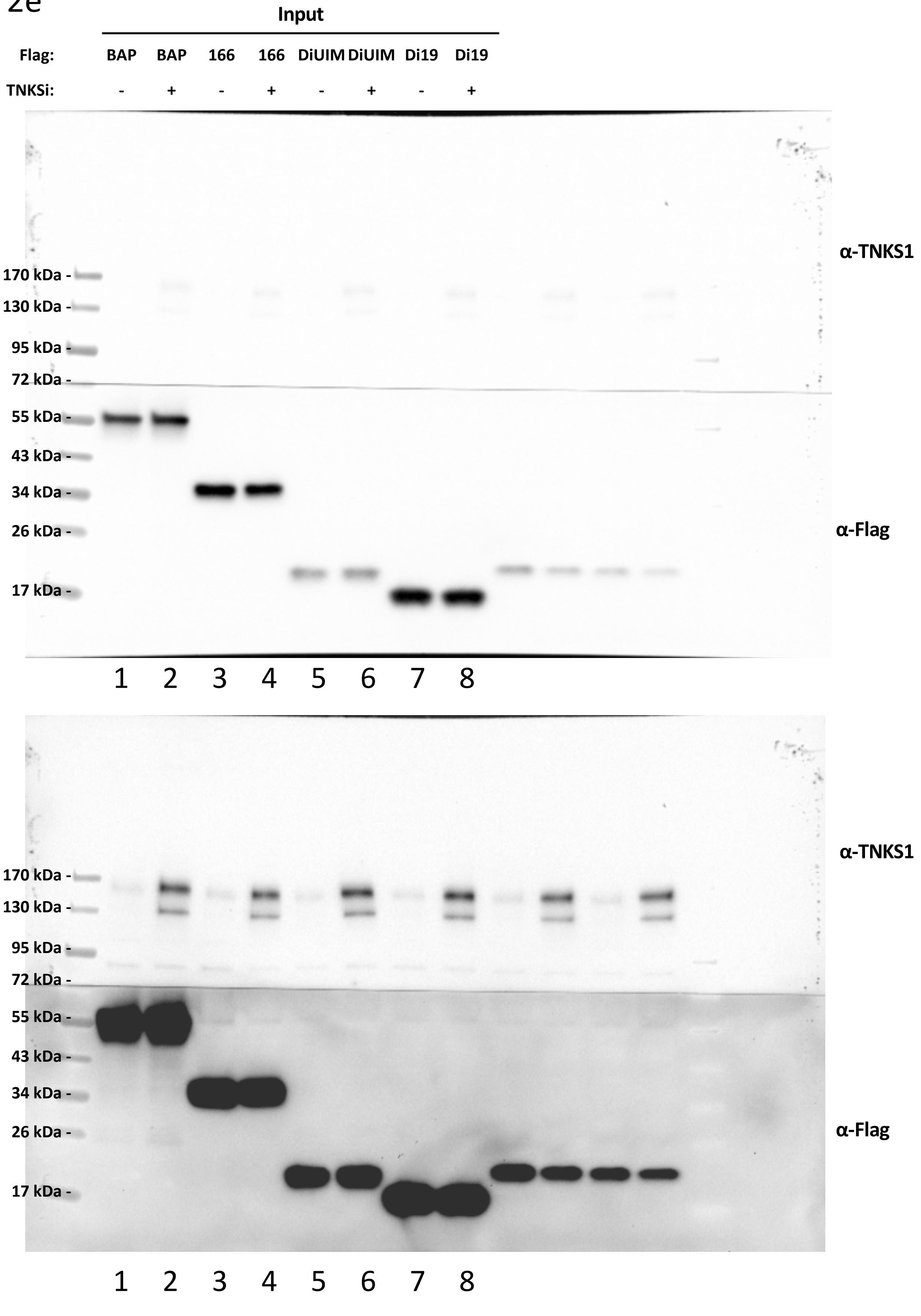
Input
Flag: BAP BAP 166 166 DiUIM DiUIM Di19 Di19
TNKSi: - + - + - + - +
170 kDa -
130 kDa -
95 kDa -
72 kDa -
55 kDa -
43 kDa -
34 kDa -
26 kDa -
17 kDa -
α-TNKS1
α-Flag
1 2 3 4 5 6 7 8
170 kDa -
130 kDa -
95 kDa -
72 kDa -
55 kDa -
43 kDa -
34 kDa -
26 kDa -
17 kDa -
α-TNKS1
α-Flag
1 2 3 4 5 6 7 8

2f

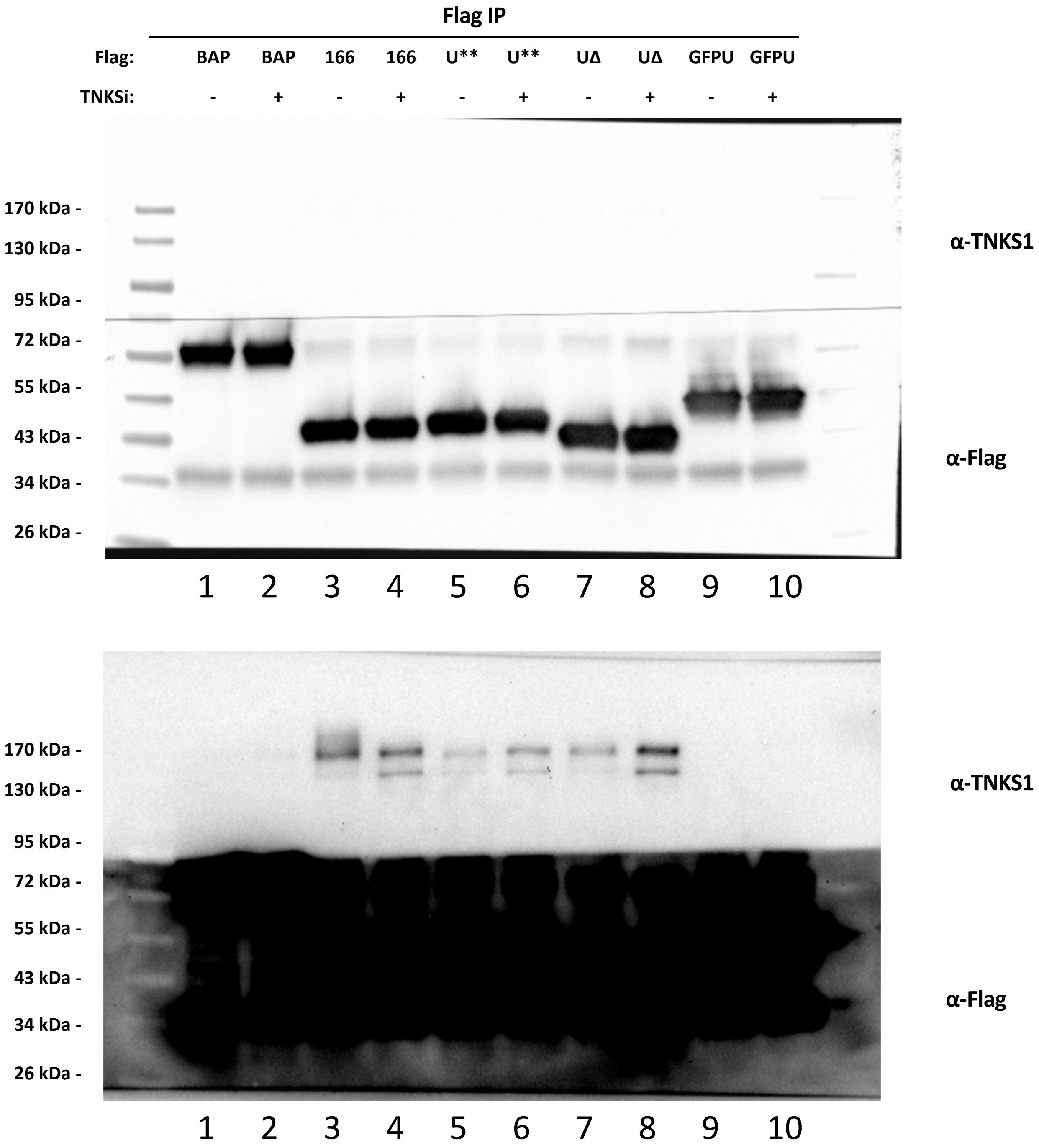
Flag IP
Flag: BAP BAP 166 166 U** U** UΔ UΔ GFPU GFPU
TNKSi: - + - + - + - + - +
170 kDa -
130 kDa -
95 kDa -
72 kDa -
55 kDa -
43 kDa -
34 kDa -
26 kDa -
α-TNKS1
α-Flag
1 2 3 4 5 6 7 8 9 10
170 kDa -
130 kDa -
95 kDa -
72 kDa -
55 kDa -
43 kDa -
34 kDa -
26 kDa -
α-TNKS1
α-Flag
1 2 3 4 5 6 7 8 9 10

2f

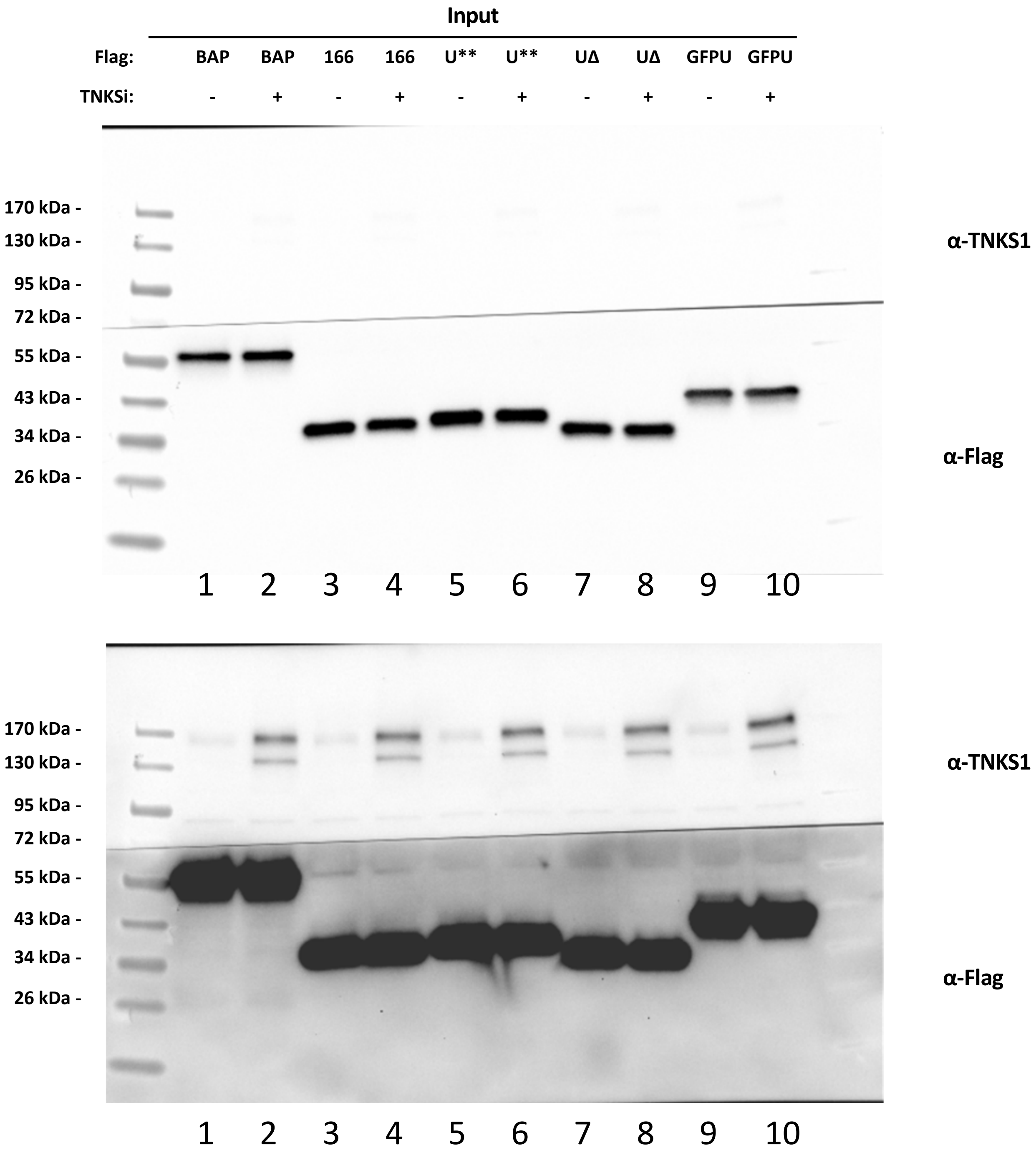
Input
Flag: BAP BAP 166 166 U** U** UΔ UΔ GFPU GFPU
TNKSi: - + - + - + - + - +
170 kDa -
130 kDa -
95 kDa -
72 kDa -
55 kDa -
43 kDa -
34 kDa -
26 kDa -
α-TNKS1
α-Flag
1 2 3 4 5 6 7 8 9 10
170 kDa -
130 kDa -
95 kDa -
72 kDa -
55 kDa -
43 kDa -
34 kDa -
26 kDa -
α-TNKS1
α-Flag
1 2 3 4 5 6 7 8 9 10

3a

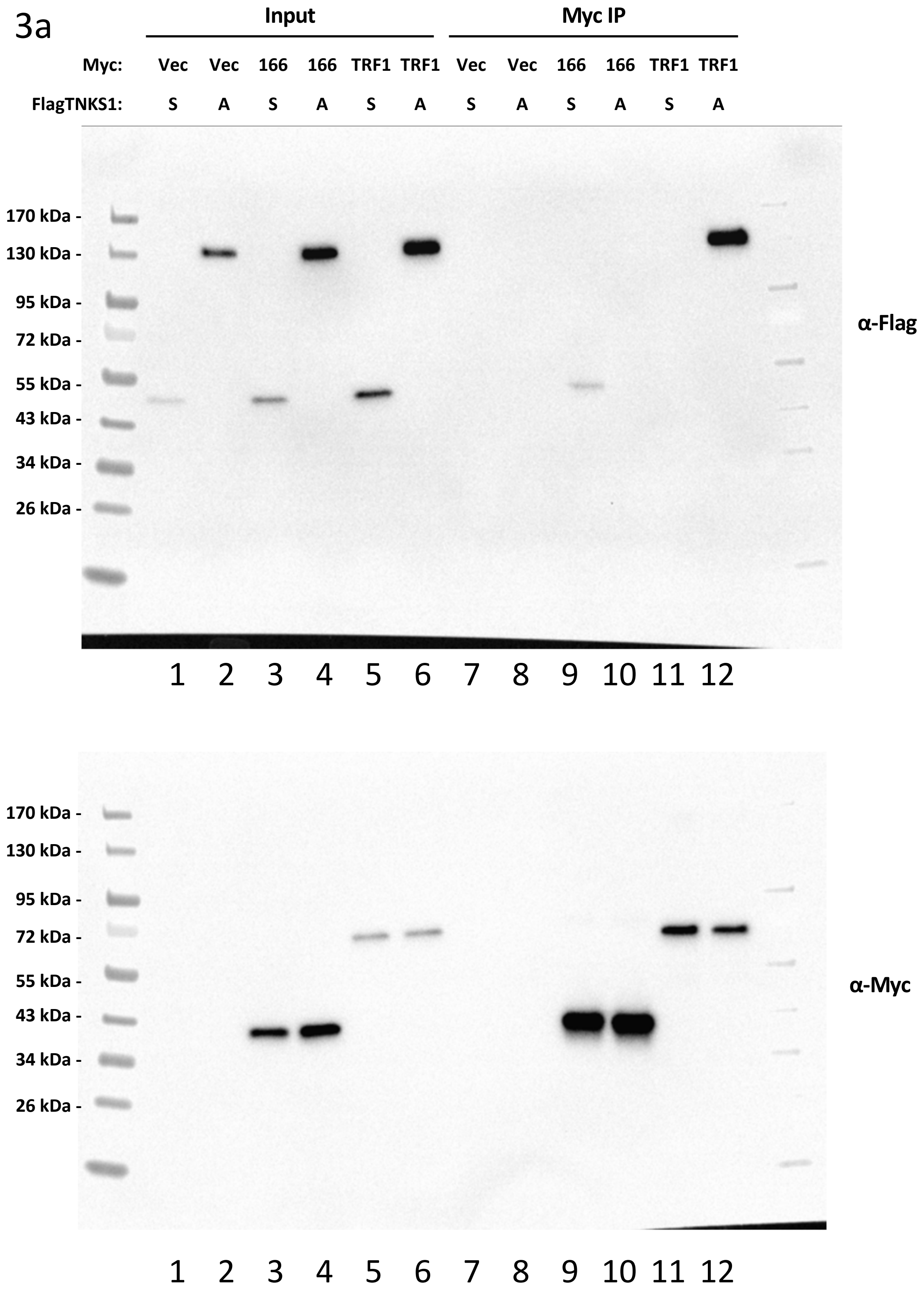
Input
Myc IP
Myc: Vec Vec 166 166 TRF1 TRF1 Vec Vec 166 166 TRF1 TRF1
FlagTNKS1: S A S A S A S A S A S A
170 kDa
130 kDa
95 kDa
72 kDa
55 kDa
43 kDa
34 kDa
26 kDa
α-Flag
1 2 3 4 5 6 7 8 9 10 11 12
170 kDa
130 kDa
95 kDa
72 kDa
55 kDa
43 kDa
34 kDa
26 kDa
α-Myc
1 2 3 4 5 6 7 8 9 10 11 12

3b

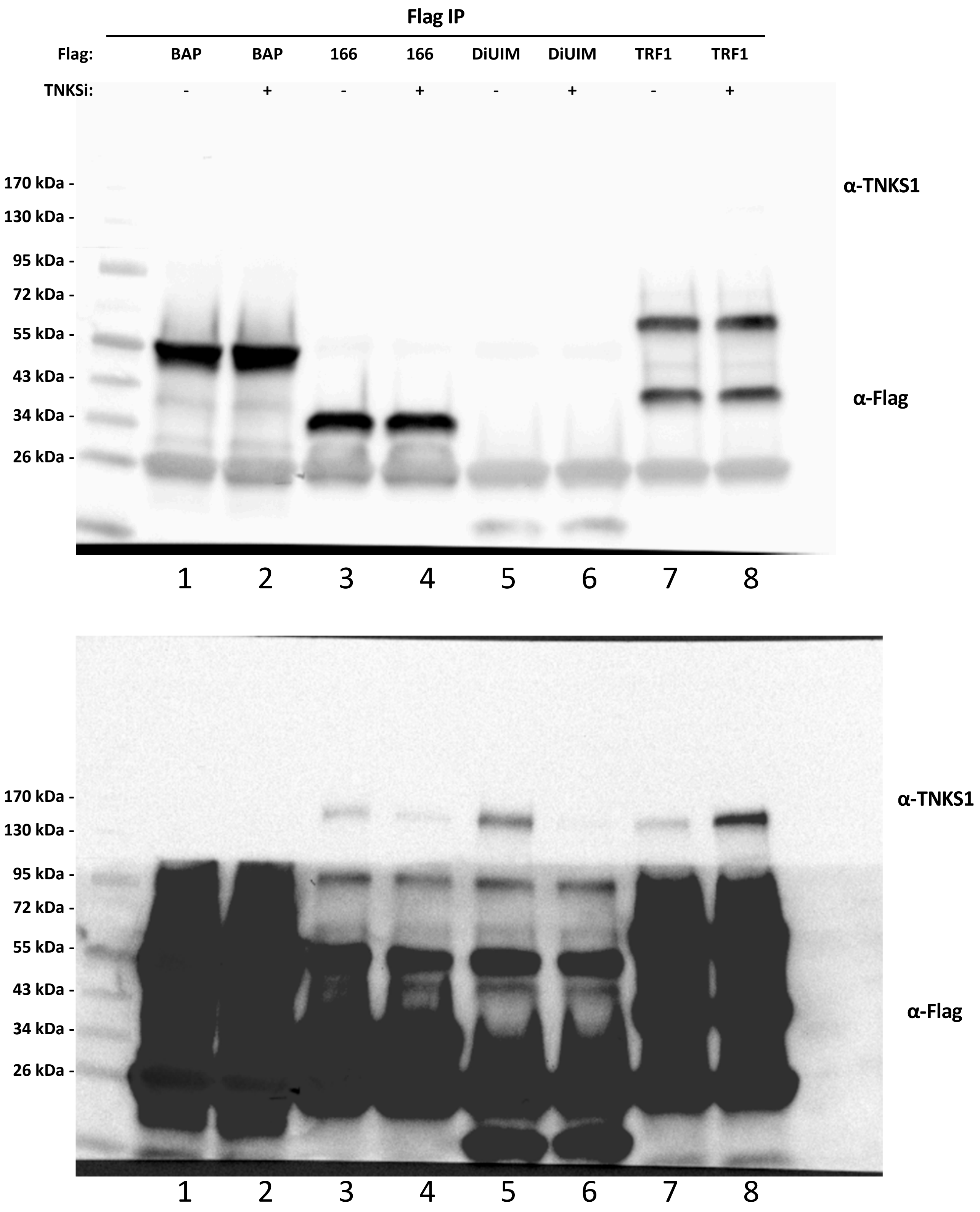
Flag IP
Flag: BAP BAP 166 166 DiUIM DiUIM TRF1 TRF1
TNKSi: - + - + - + - +
170 kDa -
130 kDa -
95 kDa -
72 kDa -
55 kDa -
43 kDa -
34 kDa -
26 kDa -
α-TNKS1
α-Flag
1 2 3 4 5 6 7 8
170 kDa -
130 kDa -
95 kDa -
72 kDa -
55 kDa -
43 kDa -
34 kDa -
26 kDa -
α-TNKS1
α-Flag
1 2 3 4 5 6 7 8

3b

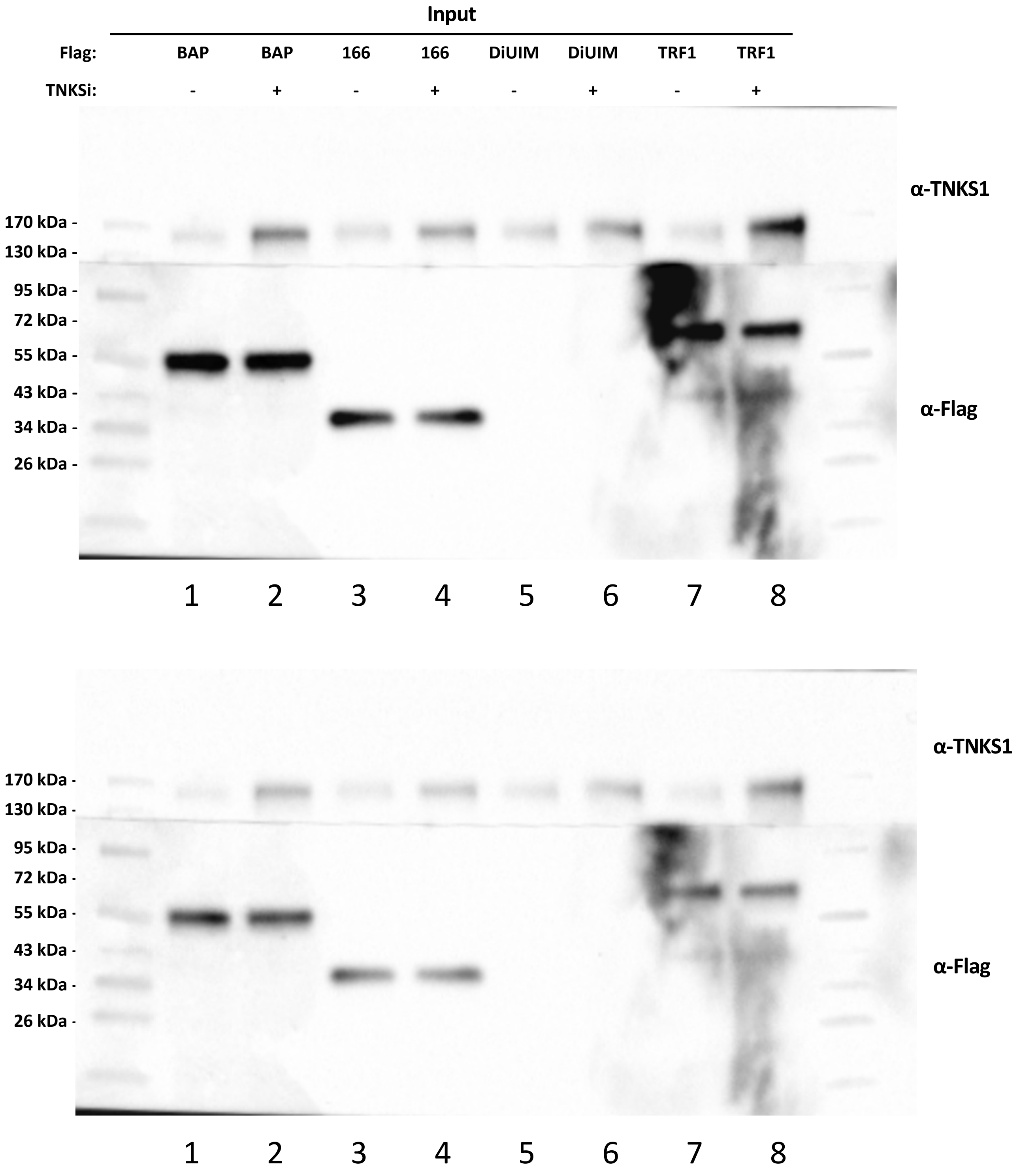

Input
Flag: BAP BAP 166 166 DiUIM DiUIM TRF1 TRF1
TNKSi: - + - + - + - +
170 kDa -
130 kDa -
95 kDa -
72 kDa -
55 kDa -
43 kDa -
34 kDa -
26 kDa -
α-TNKS1
α-Flag
1 2 3 4 5 6 7 8
170 kDa -
130 kDa -
95 kDa -
72 kDa -
55 kDa -
43 kDa -
34 kDa -
26 kDa -
α-TNKS1
α-Flag
1 2 3 4 5 6 7 8

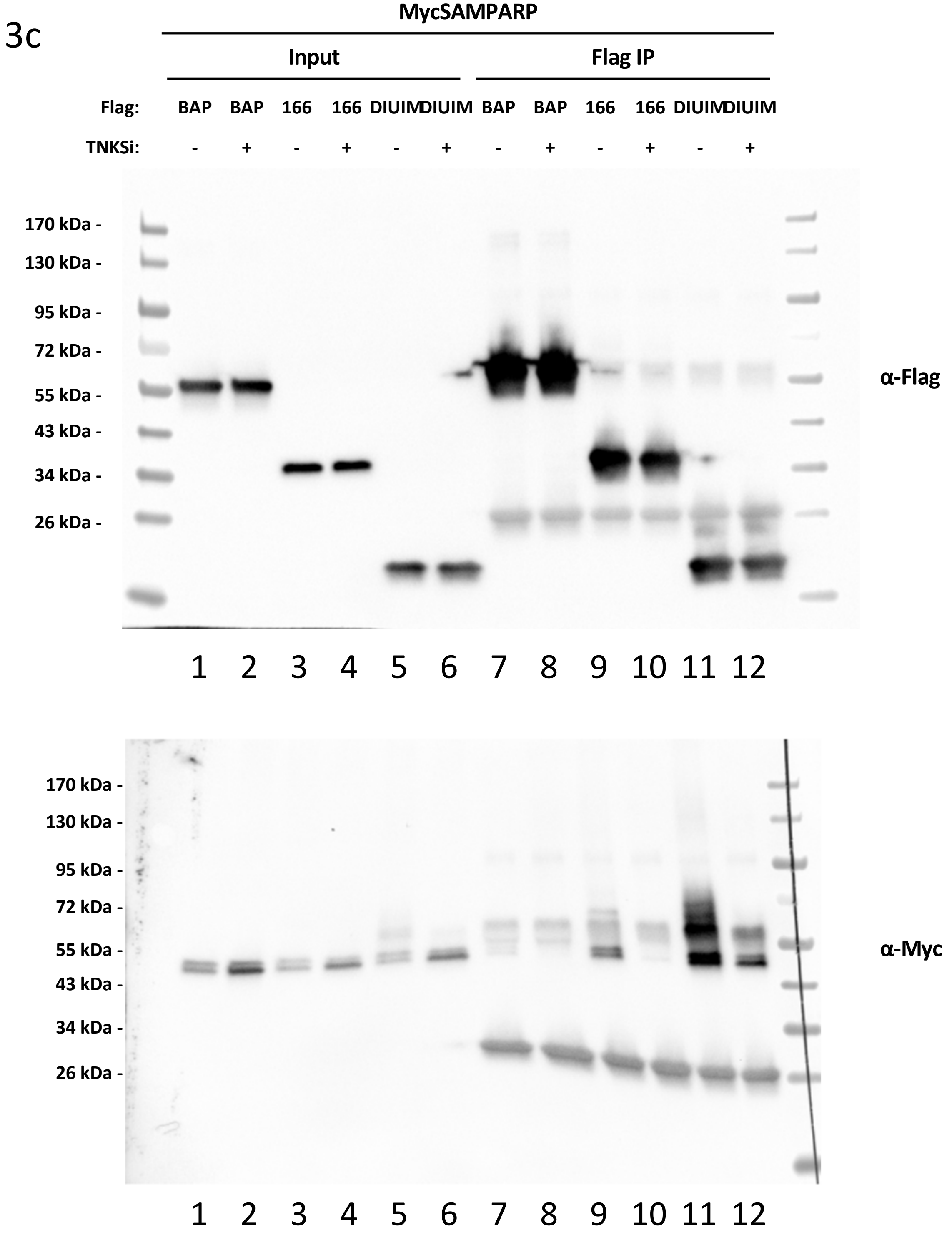
3c
MycSAMPARP
Input
Flag IP
Flag: BAP BAP 166 166 DIUIM DIUIM BAP BAP 166 166 DIUIM DIUIM
TNKSi: - + - + - + - + - + - +
170 kDa -
130 kDa -
95 kDa -
72 kDa -
55 kDa -
43 kDa -
34 kDa -
26 kDa -
α-Flag
1 2 3 4 5 6 7 8 9 10 11 12
170 kDa -
130 kDa -
95 kDa -
72 kDa -
55 kDa -
43 kDa -
34 kDa -
26 kDa -
α-Myc
1 2 3 4 5 6 7 8 9 10 11 12

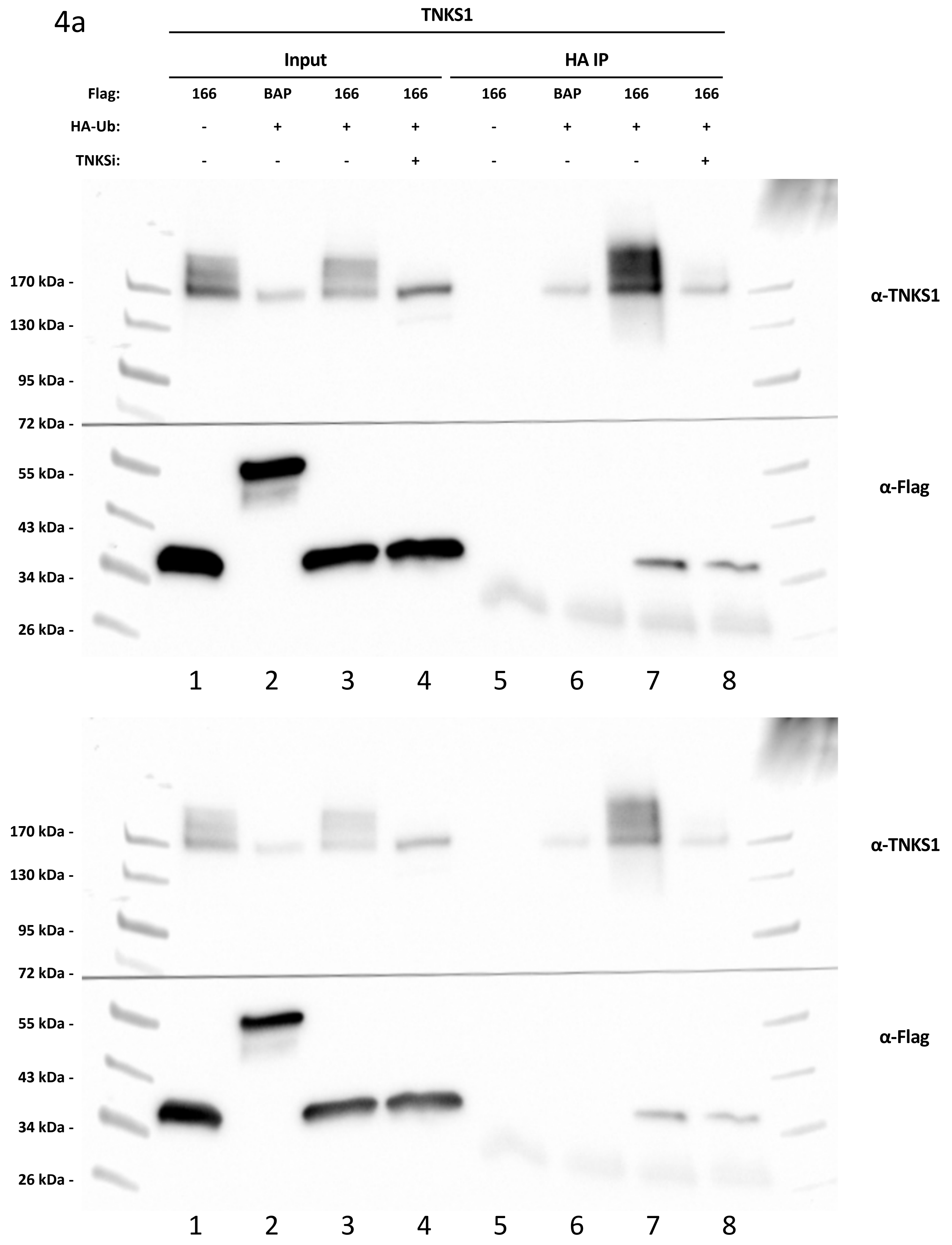
4a
TNKS1
Input
HA IP
Flag: 166 BAP 166 166 166 BAP 166 166
HA-Ub: - + + + - + + +
TNKSi: - - - + - - - +
170 kDa -
130 kDa -
95 kDa -
72 kDa -
55 kDa -
43 kDa -
34 kDa -
26 kDa -
α-TNKS1
α-Flag
1 2 3 4 5 6 7 8
170 kDa -
130 kDa -
95 kDa -
72 kDa -
55 kDa -
43 kDa -
34 kDa -
26 kDa -
α-TNKS1
α-Flag
1 2 3 4 5 6 7 8

4b

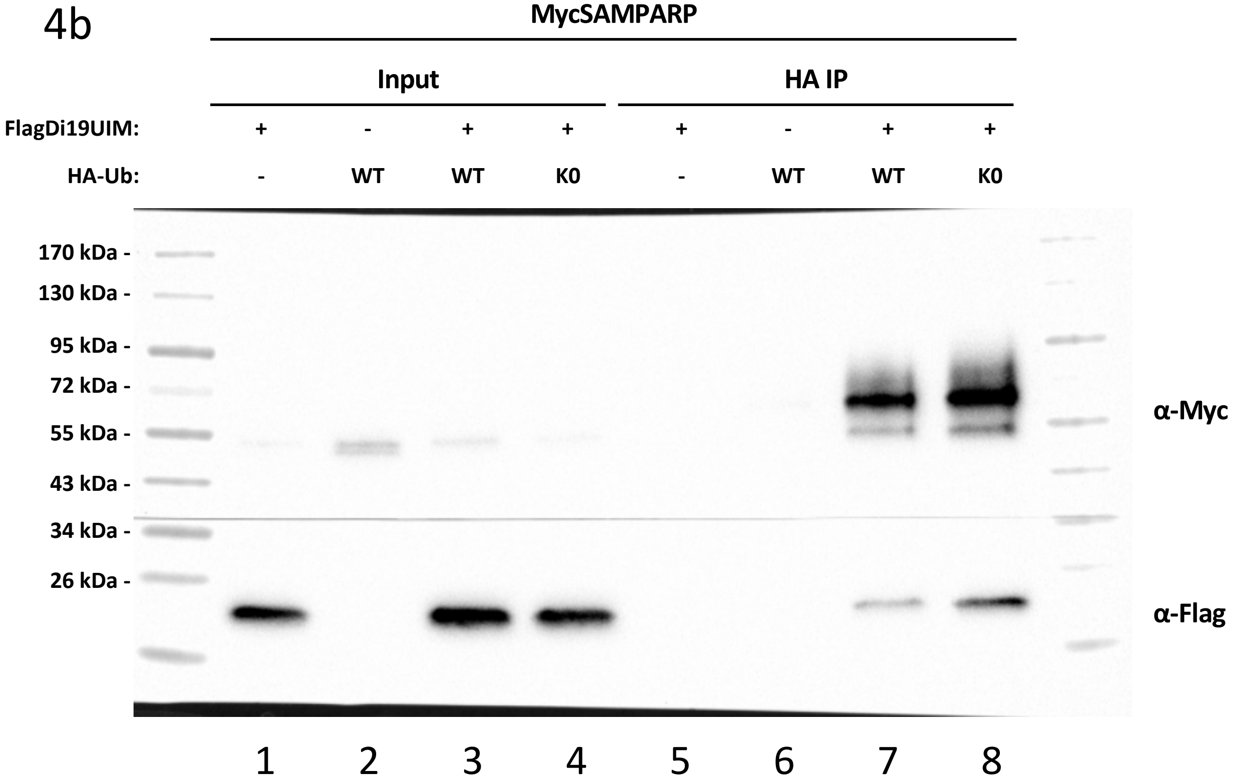

MycSAMPARP
Input
HA IP
FlagDi19UIM: + - + + + - + +
HA-Ub: - WT WT K0 - WT WT K0
170 kDa -
130 kDa -
95 kDa -
72 kDa -
55 kDa -
43 kDa -
34 kDa -
26 kDa -
α-Myc
α-Flag
1 2 3 4 5 6 7 8

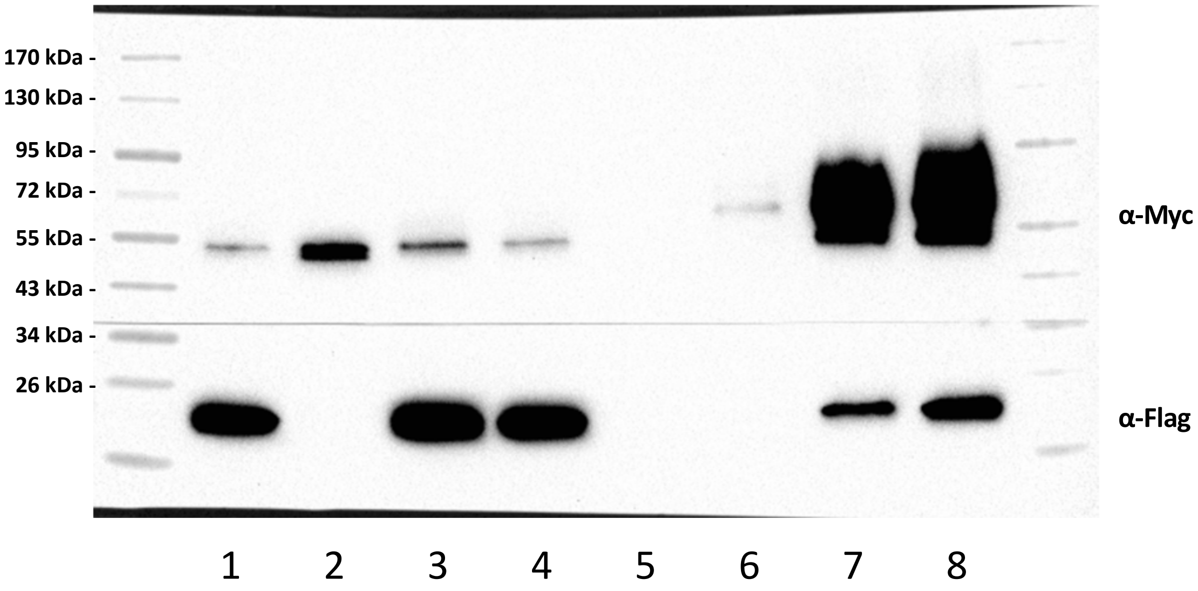

170 kDa -
130 kDa -
95 kDa -
72 kDa -
55 kDa -
43 kDa -
34 kDa -
26 kDa -
α-Myc
α-Flag
1 2 3 4 5 6 7 8

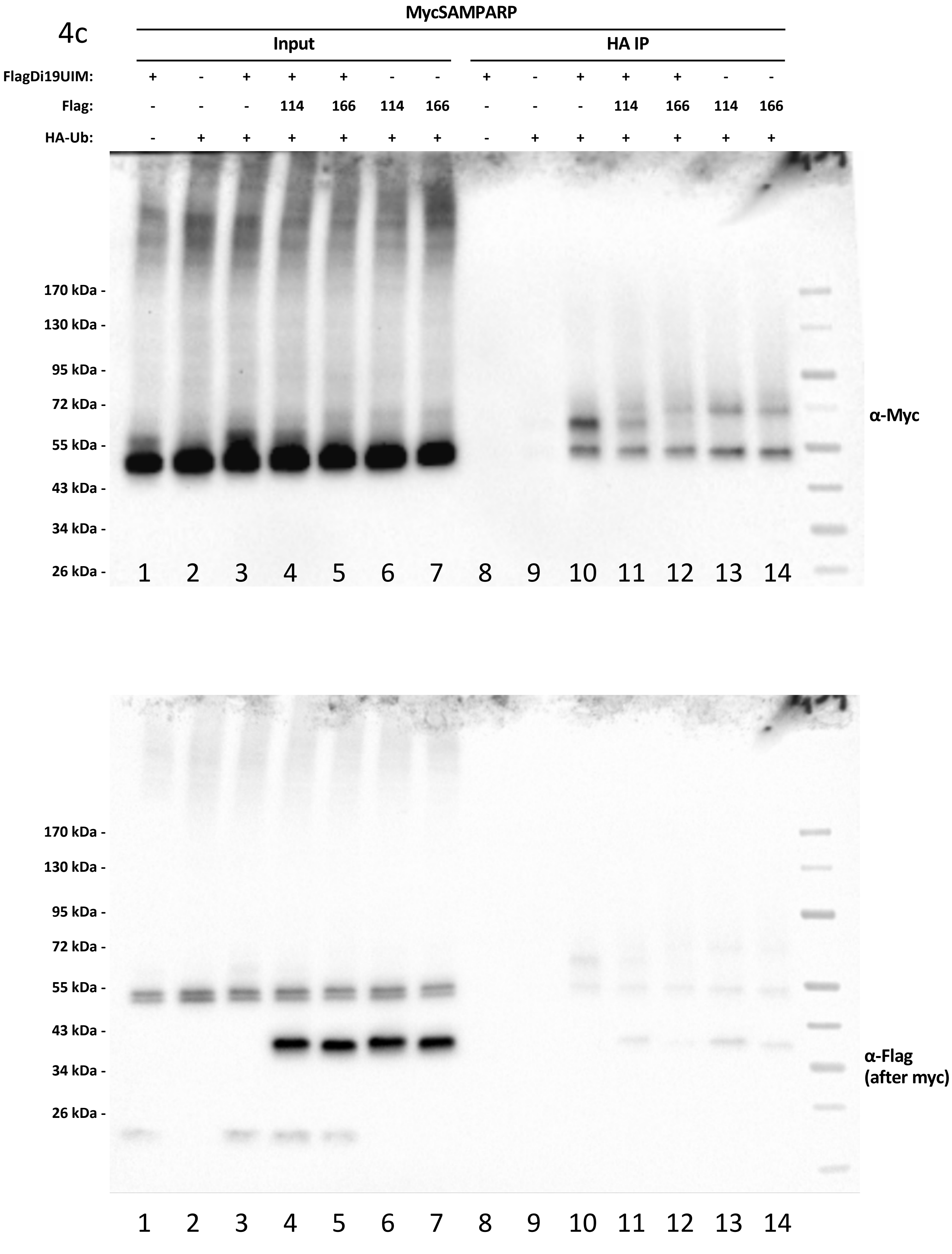

4c
MycSAMPARP
Input
HA IP
FlagDi19UIM: + - + + + - - + - + + + - -
Flag: - - - 114 166 114 166 - - - 114 166 114 166
HA-Ub: - + + + + + + - + + + + + +
170 kDa -
130 kDa -
95 kDa -
72 kDa -
55 kDa -
43 kDa -
34 kDa -
26 kDa -
1 2 3 4 5 6 7 8 9 10 11 12 13 14
α-Myc
170 kDa -
130 kDa -
95 kDa -
72 kDa -
55 kDa -
43 kDa -
34 kDa -
26 kDa -
α-Flag
(after myc)
1 2 3 4 5 6 7 8 9 10 11 12 13 14

4d

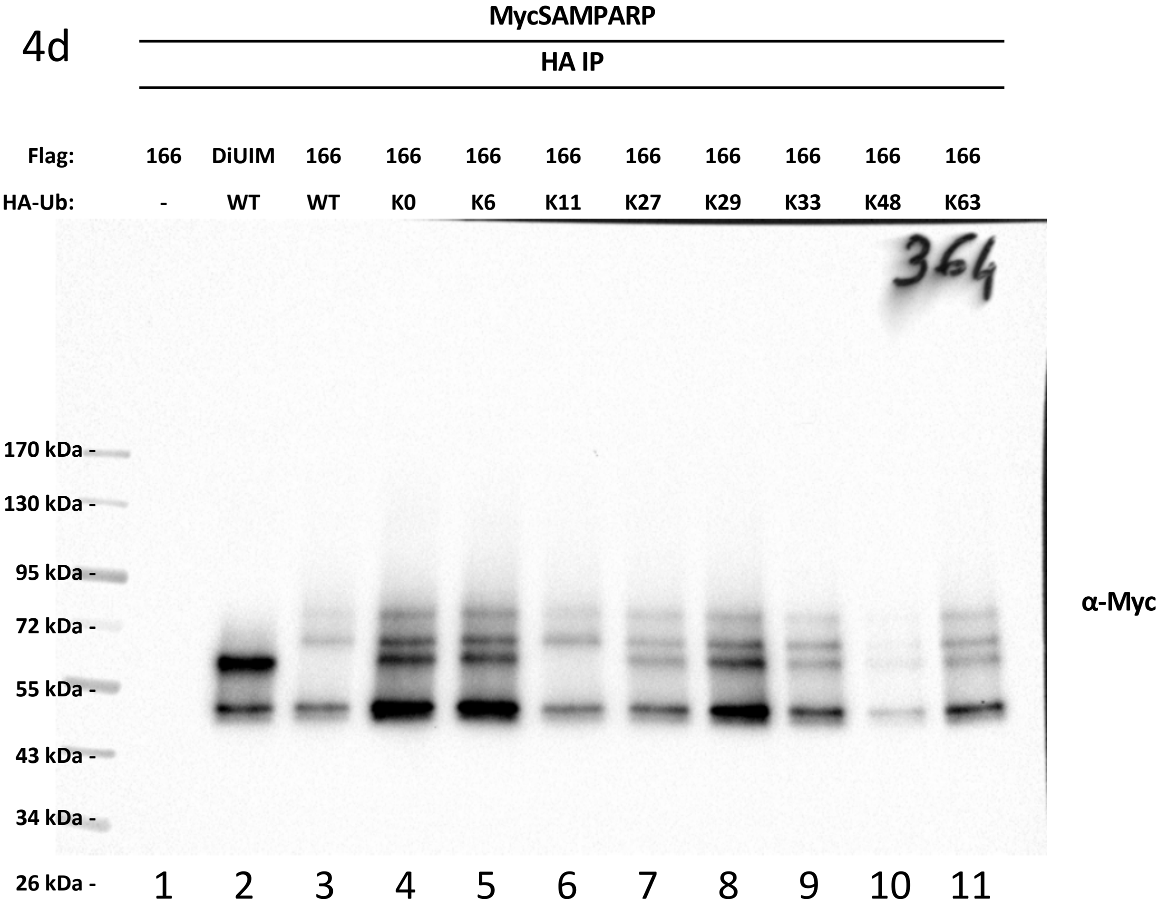
MycSAMPARP
HA IP
Flag: 166 DiUIM 166 166 166 166 166 166 166 166 166
HA-Ub: - WT WT K0 K6 K11 K27 K29 K33 K48 K63
364
170 kDa -
130 kDa -
95 kDa -
72 kDa -
55 kDa -
43 kDa -
34 kDa -
26 kDa -
1 2 3 4 5 6 7 8 9 10 11
α-Myc

4d

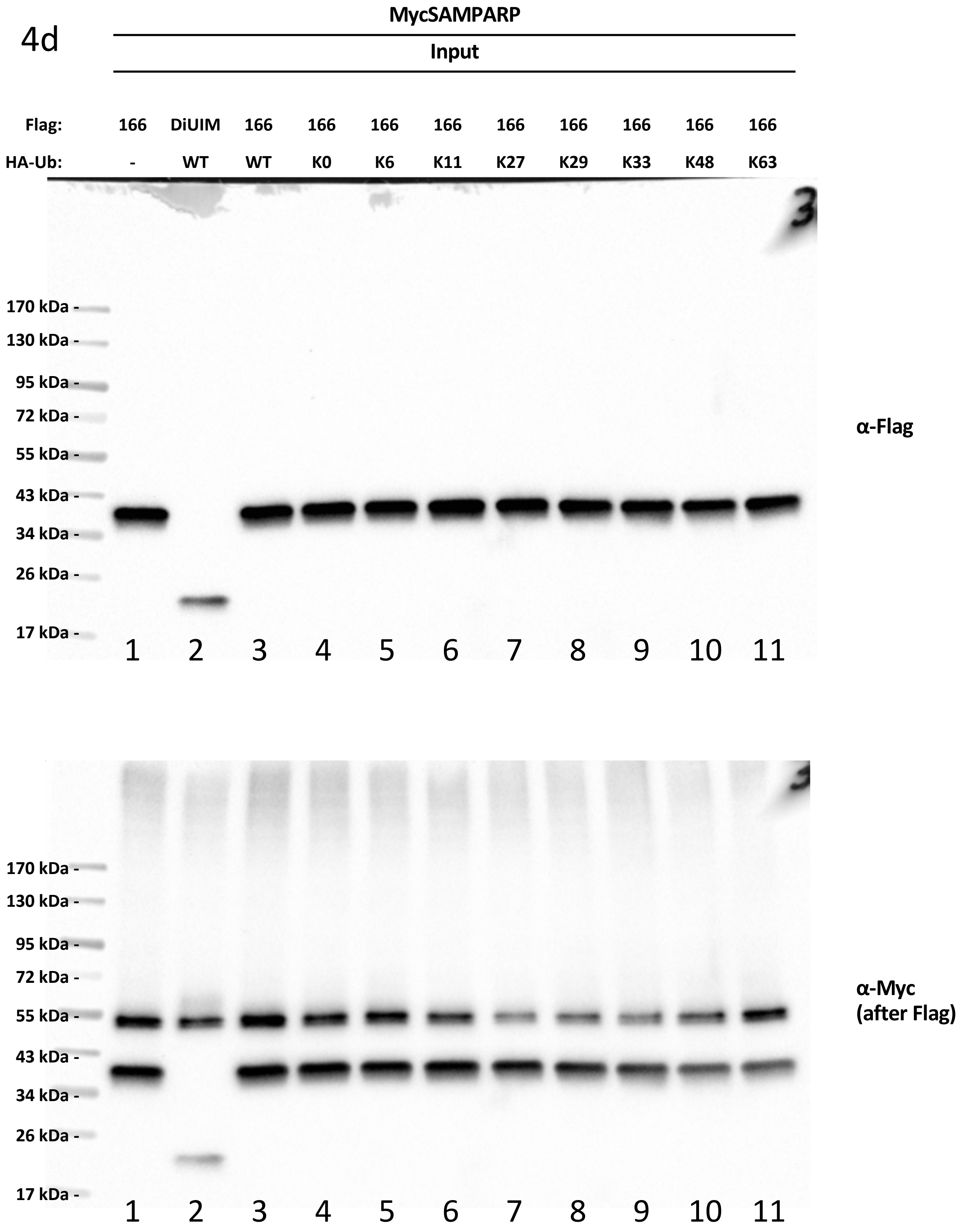
MycSAMPARP
Input
Flag:
166
DiUIM
166
166
166
166
166
166
166
166
166
HA-Ub:
-
WT
WT
K0
K6
K11
K27
K29
K33
K48
K63
170 kDa -
130 kDa -
95 kDa -
72 kDa -
55 kDa -
43 kDa -
34 kDa -
26 kDa -
17 kDa -
1 2 3 4 5 6 7 8 9 10 11
α-Flag
170 kDa -
130 kDa -
95 kDa -
72 kDa -
55 kDa -
43 kDa -
34 kDa -
26 kDa -
17 kDa -
1 2 3 4 5 6 7 8 9 10 11
α-Myc
(after Flag)

4e

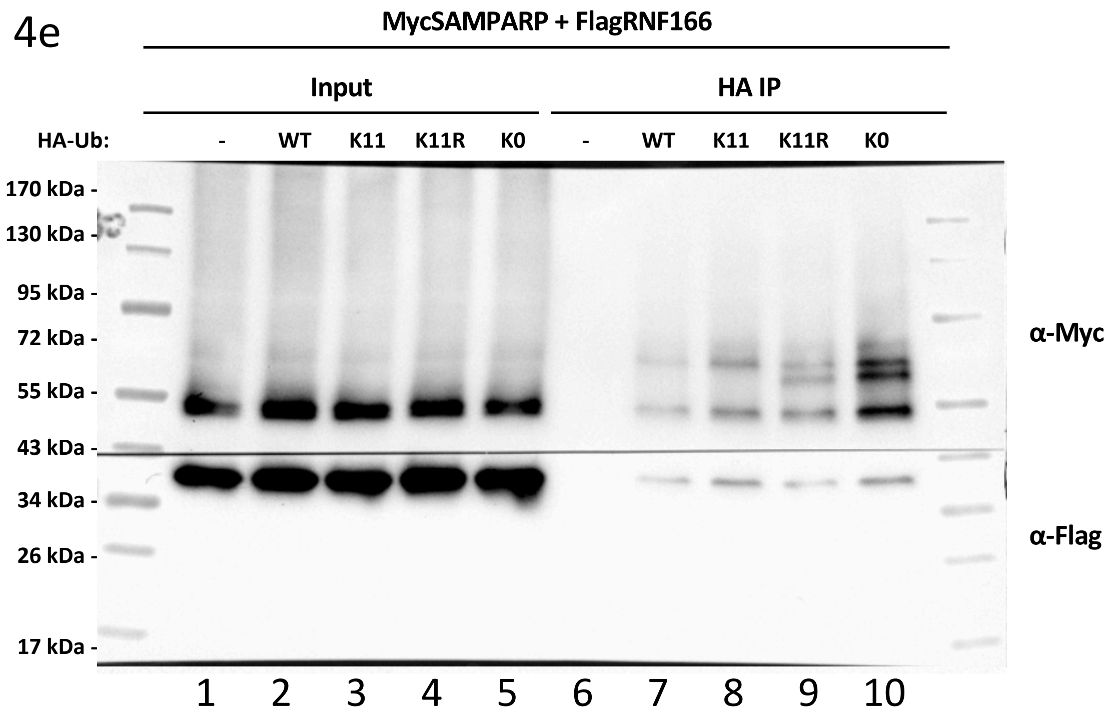
MycSAMPARP + FlagRNF166
Input
HA IP
HA-Ub:
-
WT
K11
K11R
K0
-
WT
K11
K11R
K0
170 kDa -
130 kDa -
95 kDa -
72 kDa -
55 kDa -
43 kDa -
34 kDa -
26 kDa -
17 kDa -
α-Myc
α-Flag
1
2
3
4
5
6
7
8
9
10

4f

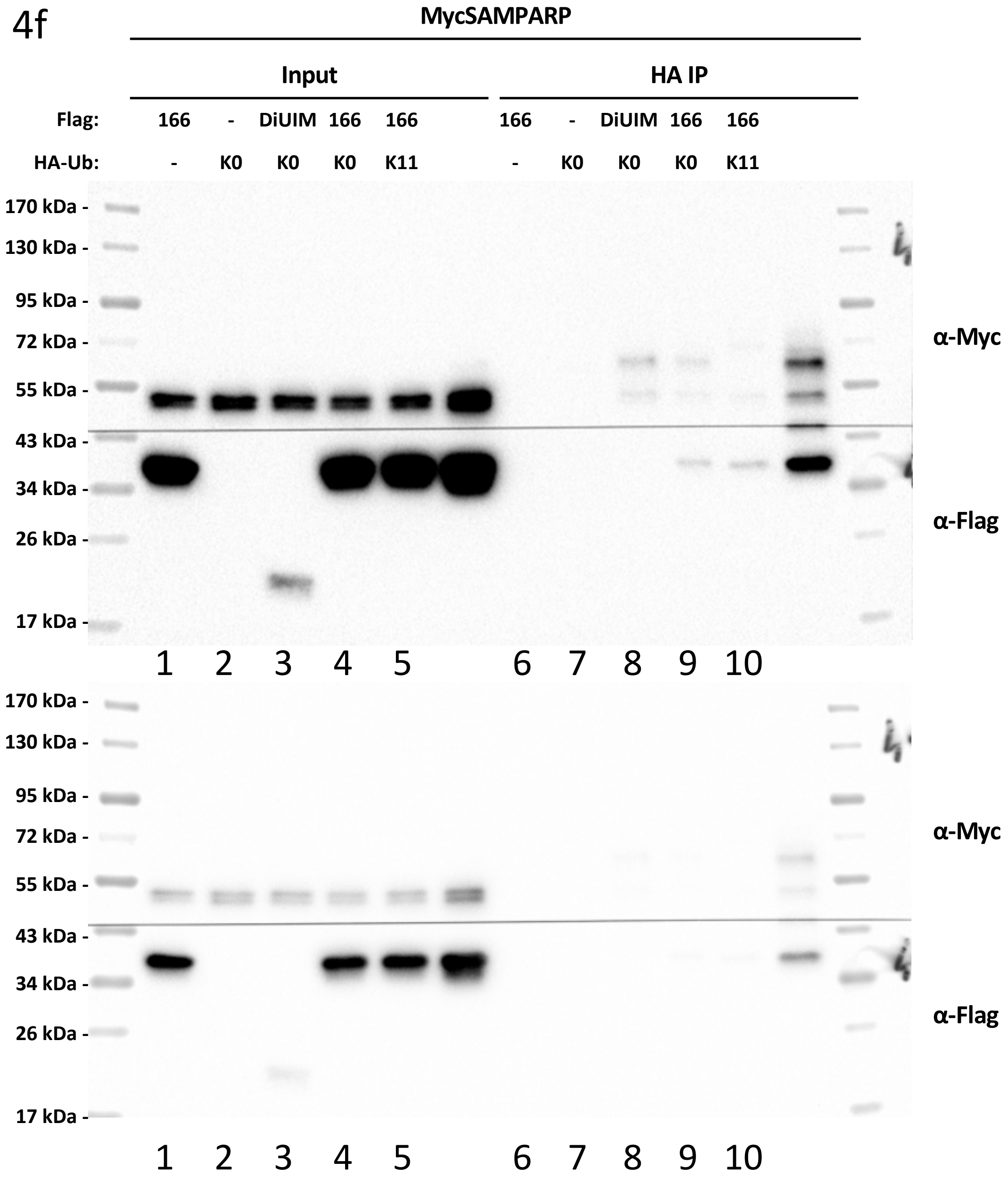
MycSAMPARP
Input
HA IP
Flag: 166 - DiUIM 166 166 166 - DiUIM 166 166
HA-Ub: - K0 K0 K0 K11 - K0 K0 K0 K11
170 kDa -
130 kDa -
95 kDa -
72 kDa -
55 kDa -
43 kDa -
34 kDa -
26 kDa -
17 kDa -
α-Myc
α-Flag
1 2 3 4 5 6 7 8 9 10
170 kDa -
130 kDa -
95 kDa -
72 kDa -
55 kDa -
43 kDa -
34 kDa -
26 kDa -
17 kDa -
α-Myc
α-Flag
1 2 3 4 5 6 7 8 9 10

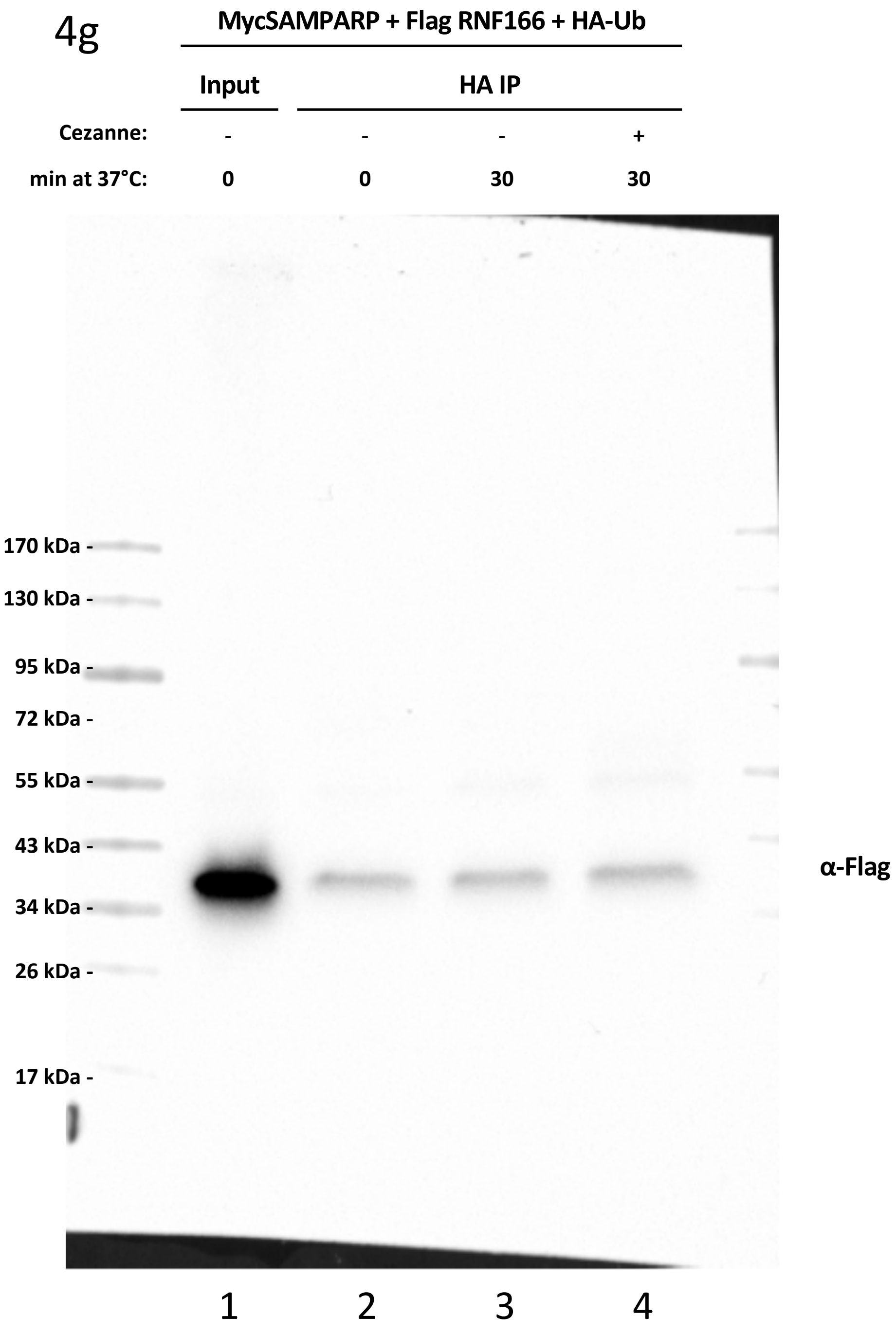
4g
MycSAMPARP + Flag RNF166 + HA-Ub
Input
HA IP
Cezanne: - - - +
min at 37°C: 0 0 30 30
170 kDa -
130 kDa -
95 kDa -
72 kDa -
55 kDa -
43 kDa -
34 kDa -
26 kDa -
17 kDa -
α-Flag
1 2 3 4

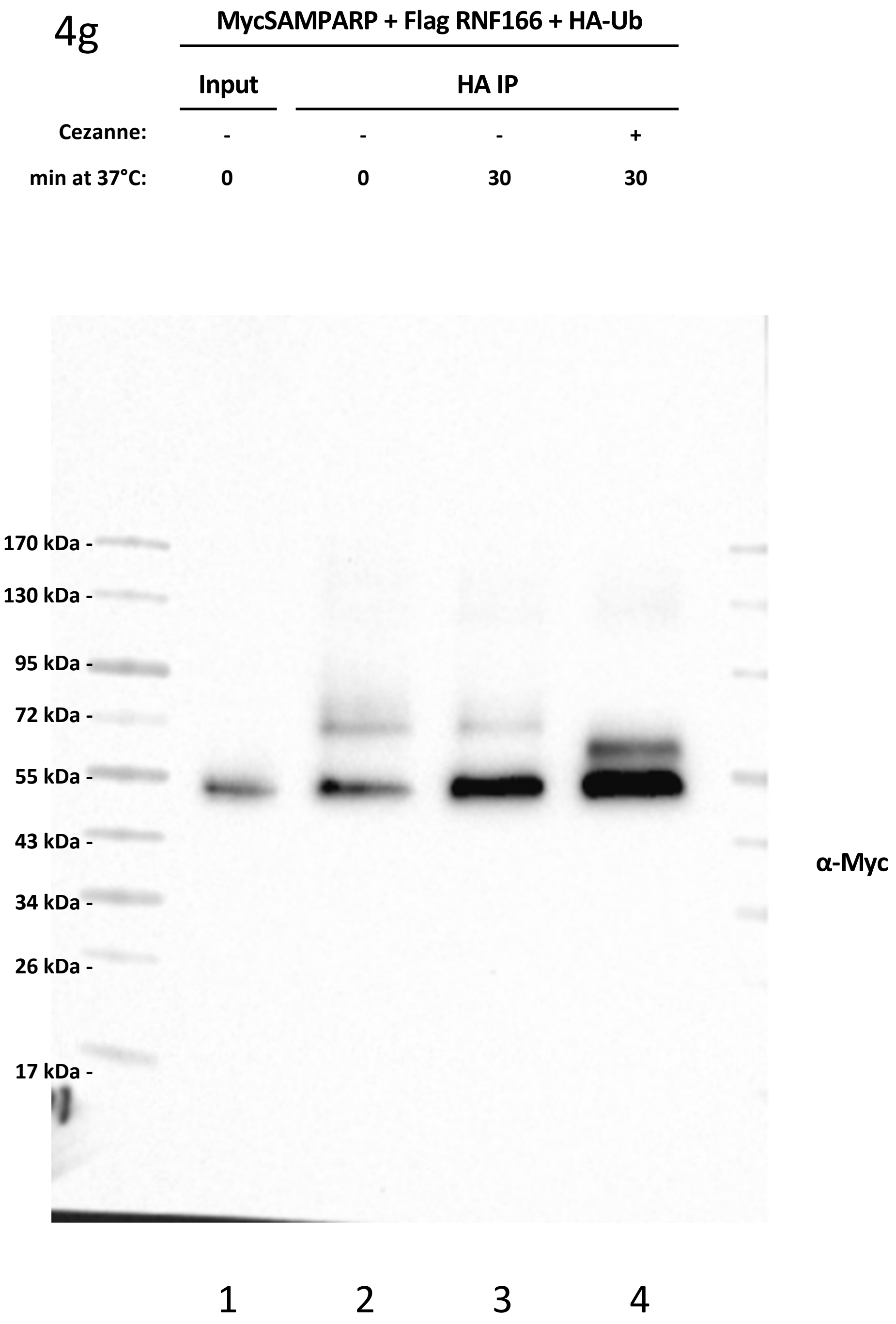
4g
MycSAMPARP + Flag RNF166 + HA-Ub
Input
HA IP
Cezanne: - - - +
min at 37°C: 0 0 30 30
170 kDa -
130 kDa -
95 kDa -
72 kDa -
55 kDa -
43 kDa -
34 kDa -
26 kDa -
17 kDa -
α-Myc
1 2 3 4

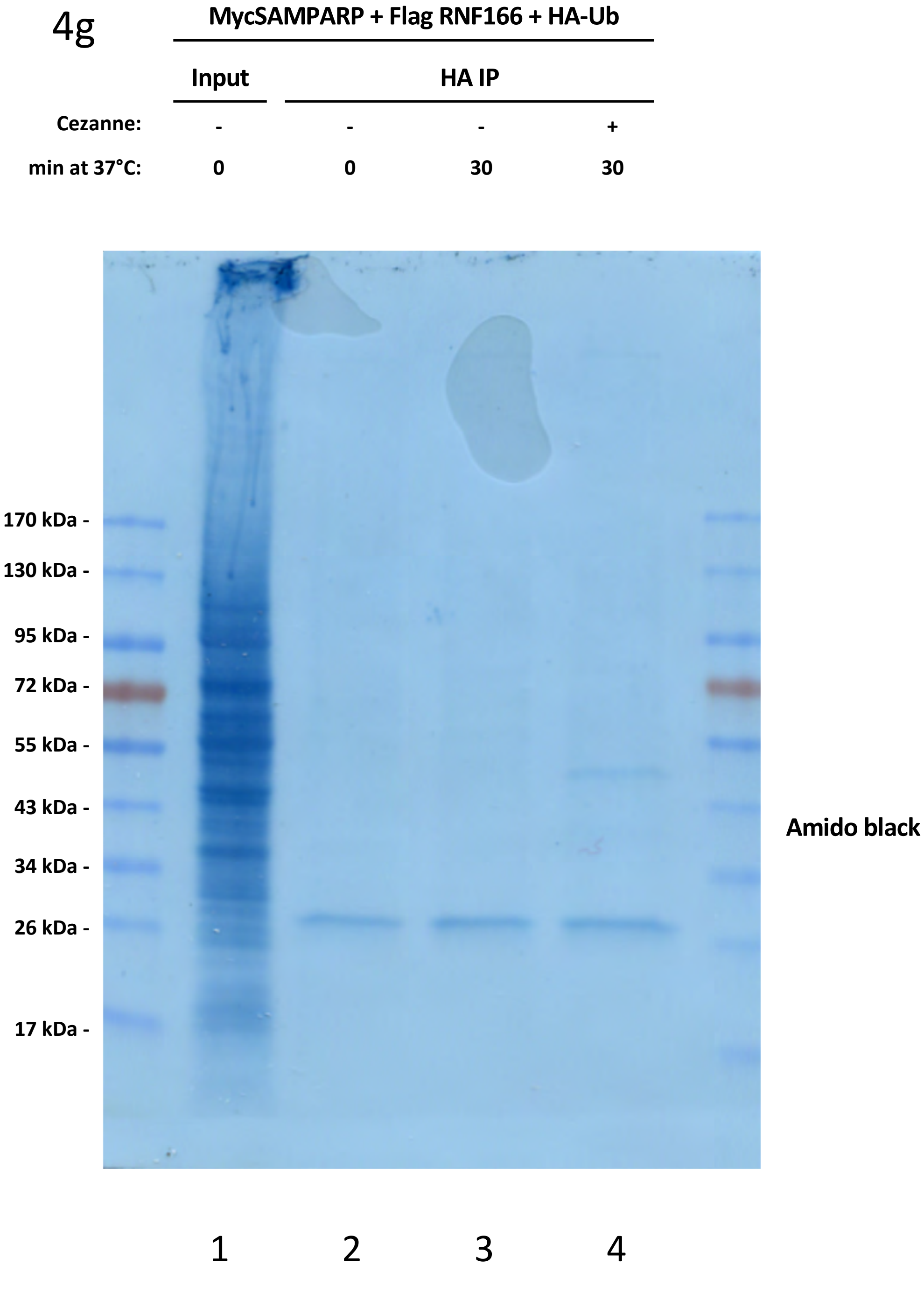
4g
MycSAMPARP + Flag RNF166 + HA-Ub
Input
HA IP
Cezanne: - - - +
min at 37°C: 0 0 30 30
170 kDa -
130 kDa -
95 kDa -
72 kDa -
55 kDa -
43 kDa -
34 kDa -
26 kDa -
17 kDa -
Amido black
1 2 3 4

4h

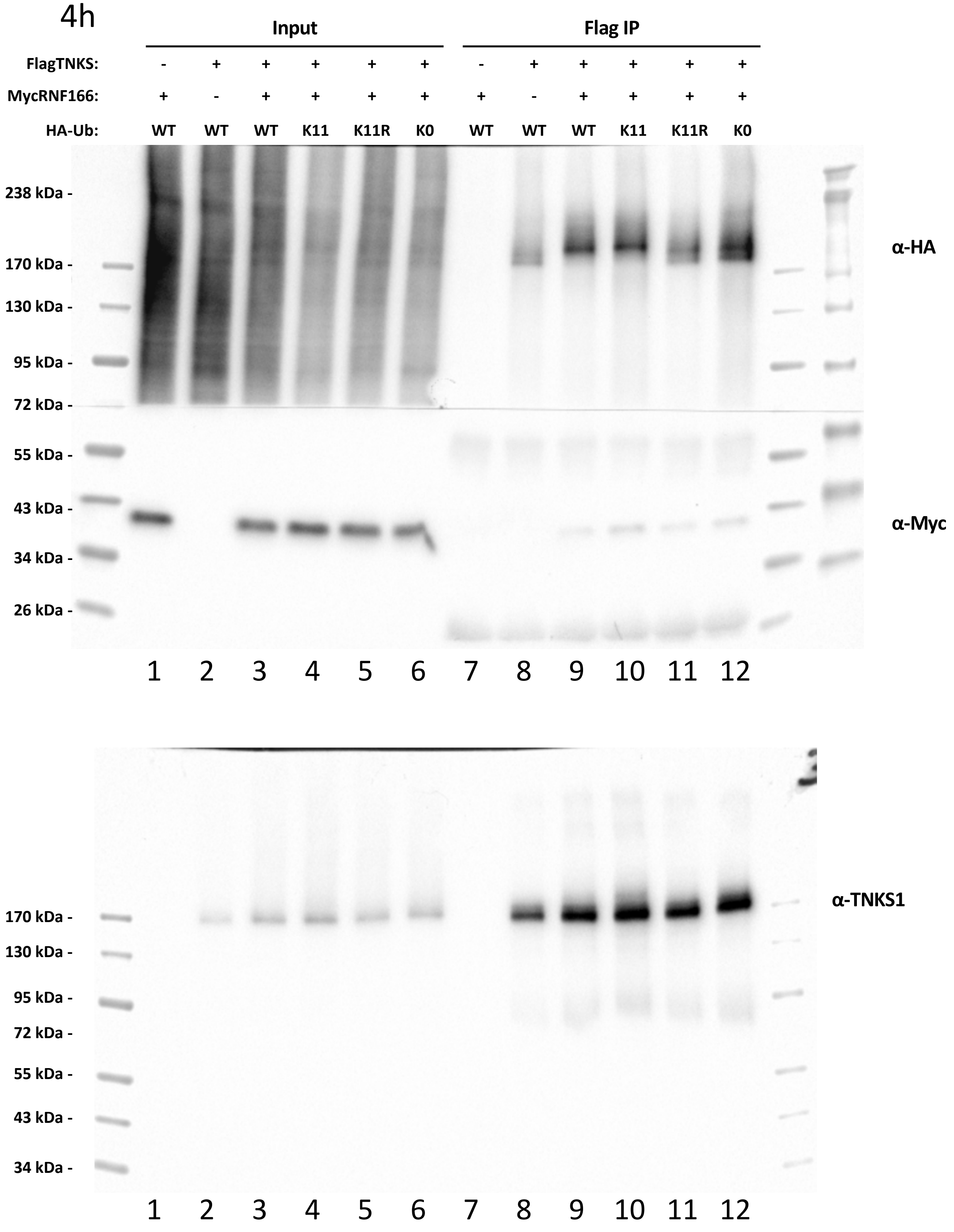
Input
Flag IP
FlagTNKS: - + + + + + - + + + + +
MycRNF166: + - + + + + + - + + + +
HA-Ub: WT WT WT K11 K11R K0 WT WT WT K11 K11R K0
238 kDa -
170 kDa -
130 kDa -
95 kDa -
72 kDa -
55 kDa -
43 kDa -
34 kDa -
26 kDa -
α-HA
α-Myc
1 2 3 4 5 6 7 8 9 10 11 12
170 kDa -
130 kDa -
95 kDa -
72 kDa -
55 kDa -
43 kDa -
34 kDa -
α-TNKS1
1 2 3 4 5 6 7 8 9 10 11 12

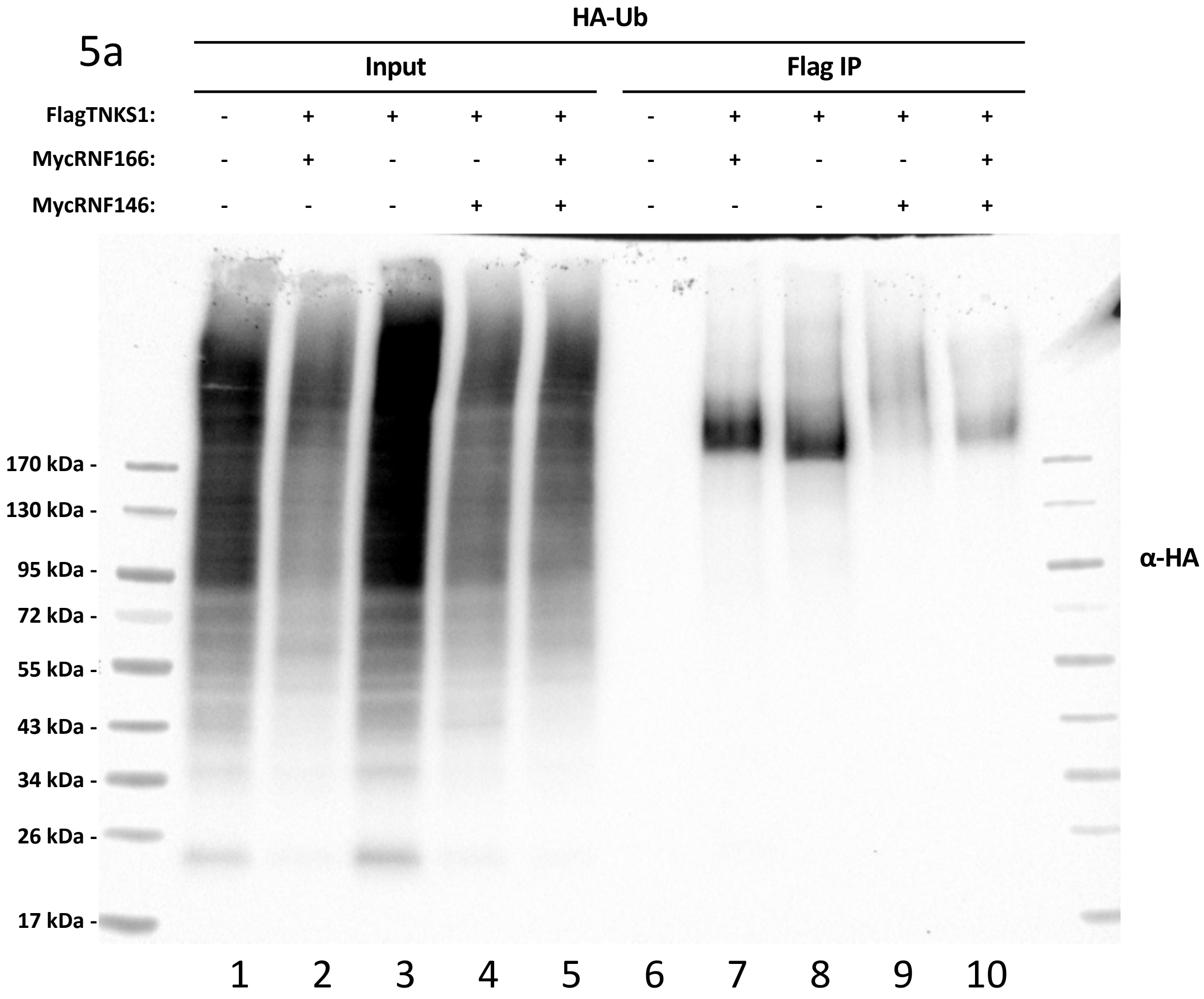
5a
HA-Ub
Input
Flag IP
FlagTNKS1: - + + + + - + + + +
MycRNF166: - + - - + - + - - +
MycRNF146: - - - + + - - - + +
170 kDa -
130 kDa -
95 kDa -
72 kDa -
55 kDa -
43 kDa -
34 kDa -
26 kDa -
17 kDa -
α-HA
1 2 3 4 5 6 7 8 9 10

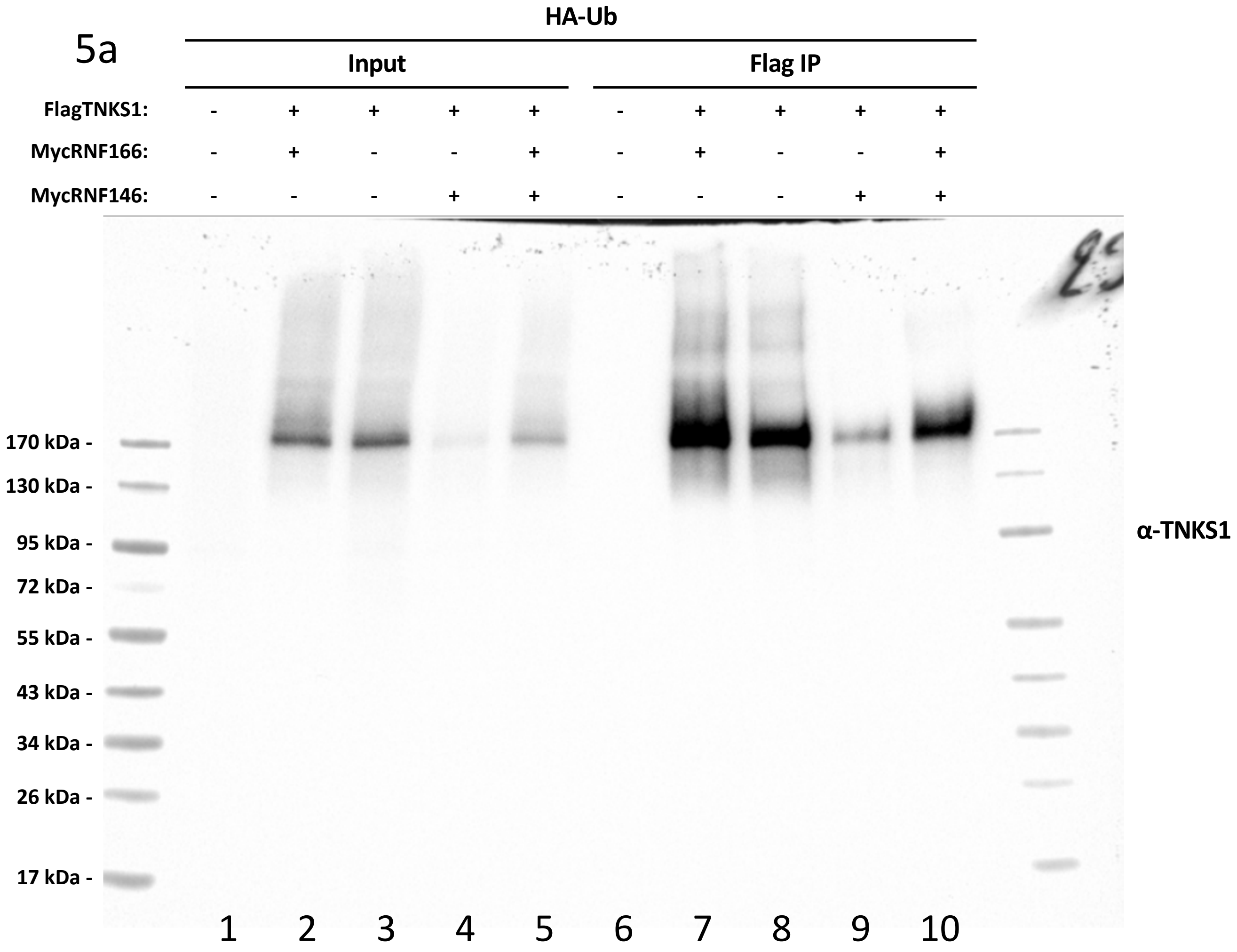
5a
HA-Ub
Input
Flag IP
FlagTNKS1: - + + + + - + + + +
MycRNF166: - + - - + - + - - +
MycRNF146: - - - + + - - - + +
170 kDa -
130 kDa -
95 kDa -
72 kDa -
55 kDa -
43 kDa -
34 kDa -
26 kDa -
17 kDa -
α-TNKS1
1 2 3 4 5 6 7 8 9 10

5a

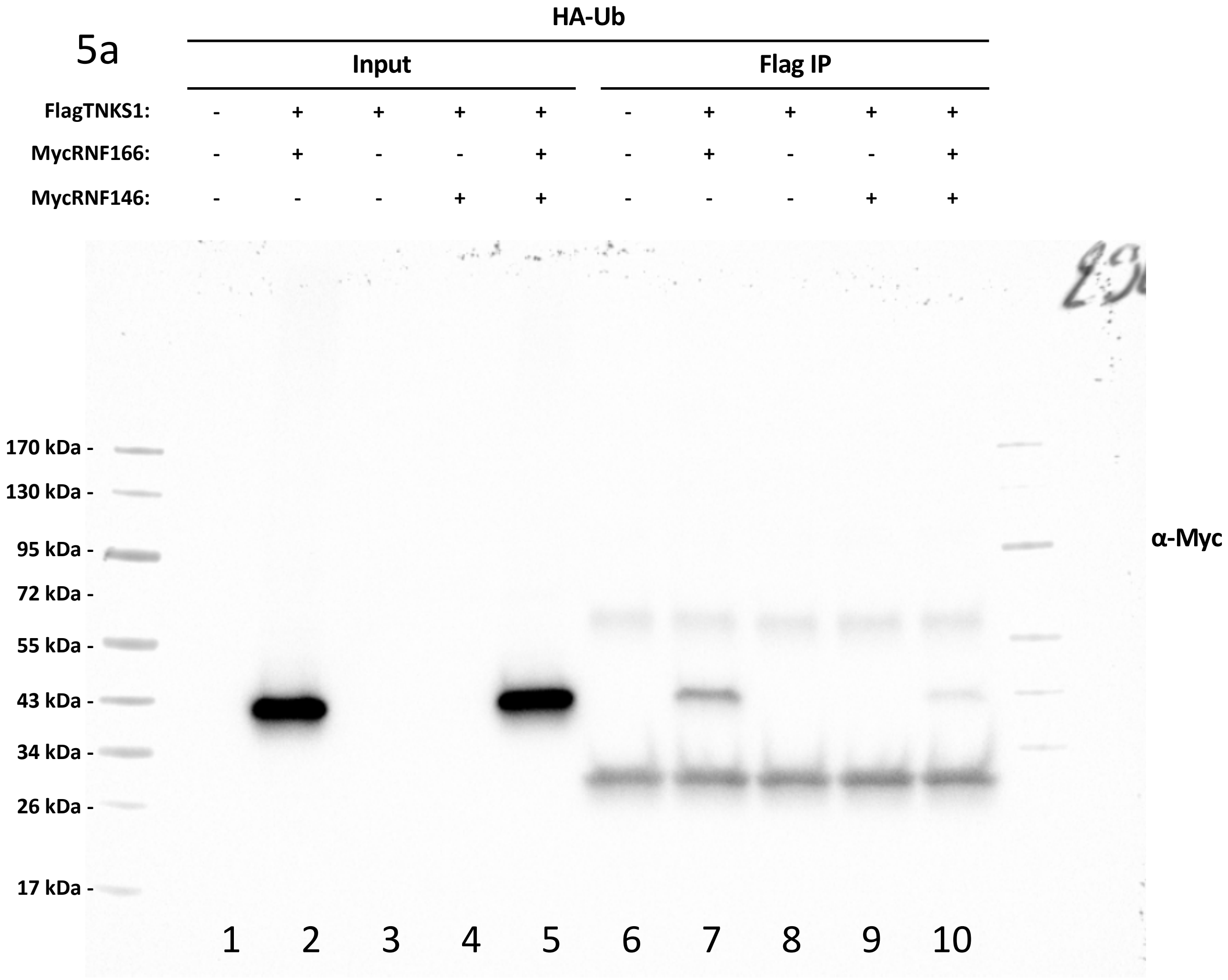
HA-Ub
Input
Flag IP
FlagTNKS1: - + + + + - + + + +
MycRNF166: - + - - + - + - - +
MycRNF146: - - - + + - - - + +
170 kDa -
130 kDa -
95 kDa -
72 kDa -
55 kDa -
43 kDa -
34 kDa -
26 kDa -
17 kDa -
α-Myc
1 2 3 4 5 6 7 8 9 10

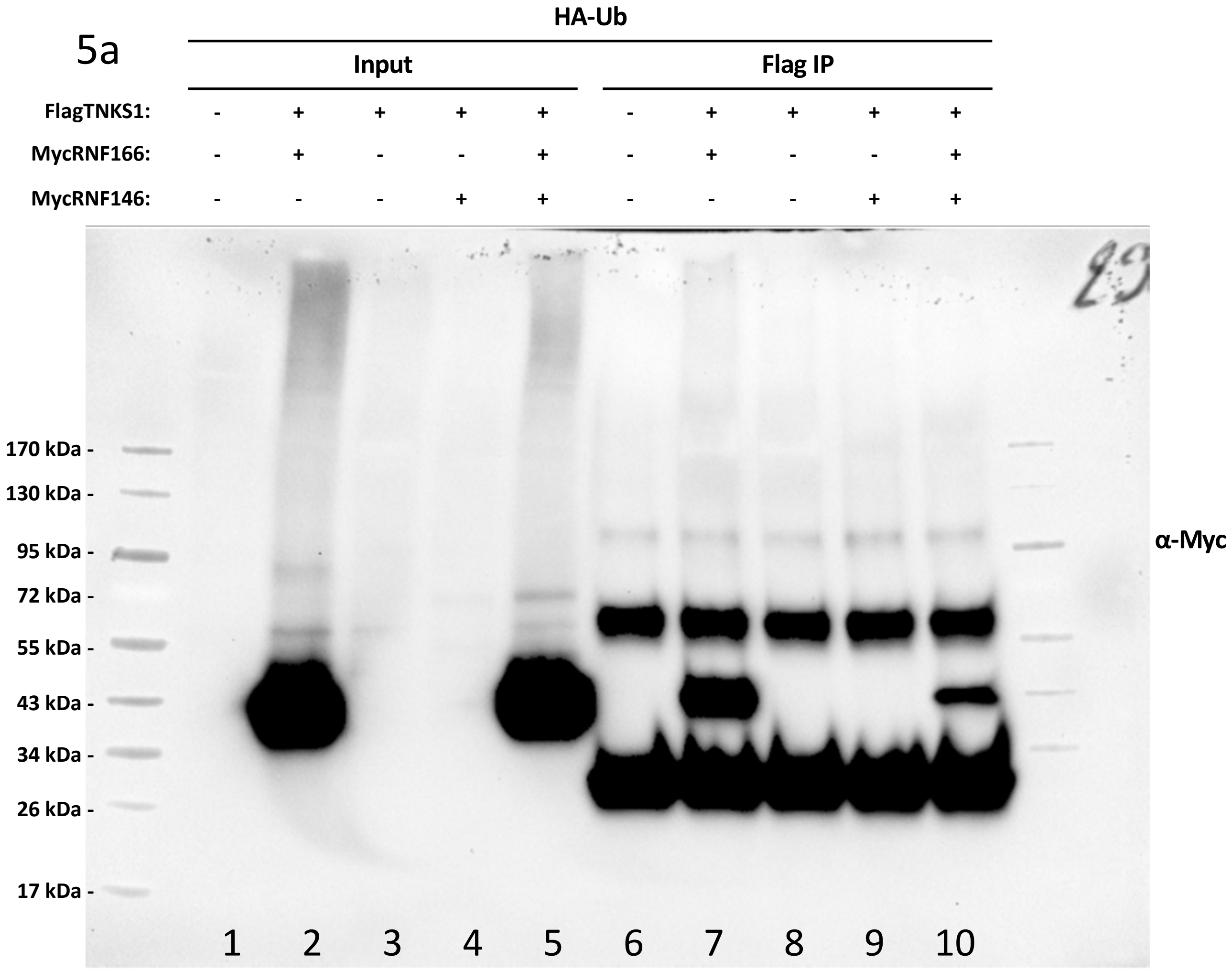
5a
HA-Ub
Input
Flag IP
FlagTNKS1: - + + + + - + + + +
MycRNF166: - + - - + - + - - +
MycRNF146: - - - + + - - - + +
170 kDa -
130 kDa -
95 kDa -
72 kDa -
55 kDa -
43 kDa -
34 kDa -
26 kDa -
17 kDa -
α-Myc
1 2 3 4 5 6 7 8 9 10

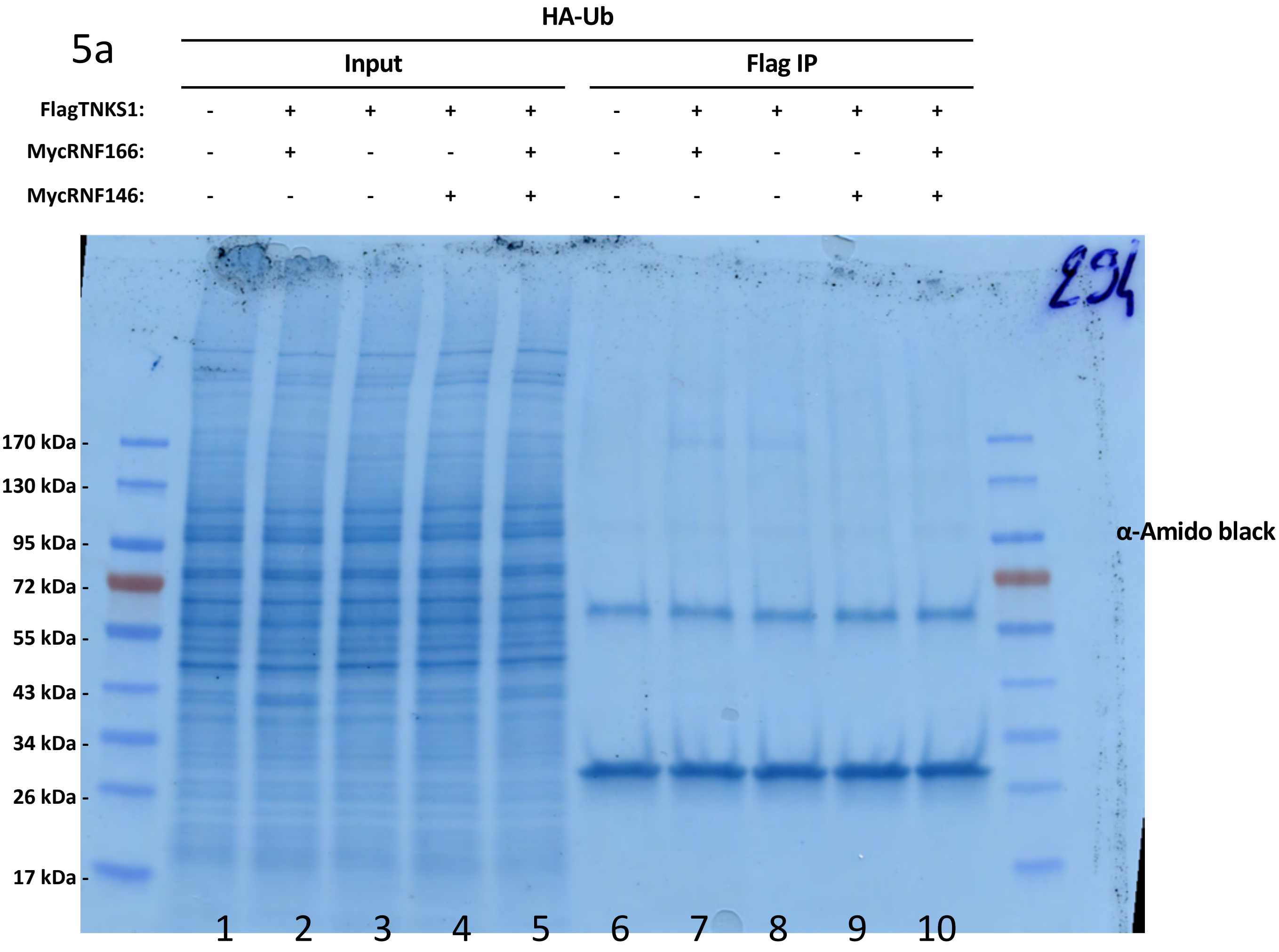
5a
HA-Ub
Input
Flag IP
FlagTNKS1: - + + + + - + + + +
MycRNF166: - + - - + - + - - +
MycRNF146: - - - + + - - - + +
170 kDa -
130 kDa -
95 kDa -
72 kDa -
55 kDa -
43 kDa -
34 kDa -
26 kDa -
17 kDa -
α-Amido black
1 2 3 4 5 6 7 8 9 10

5b

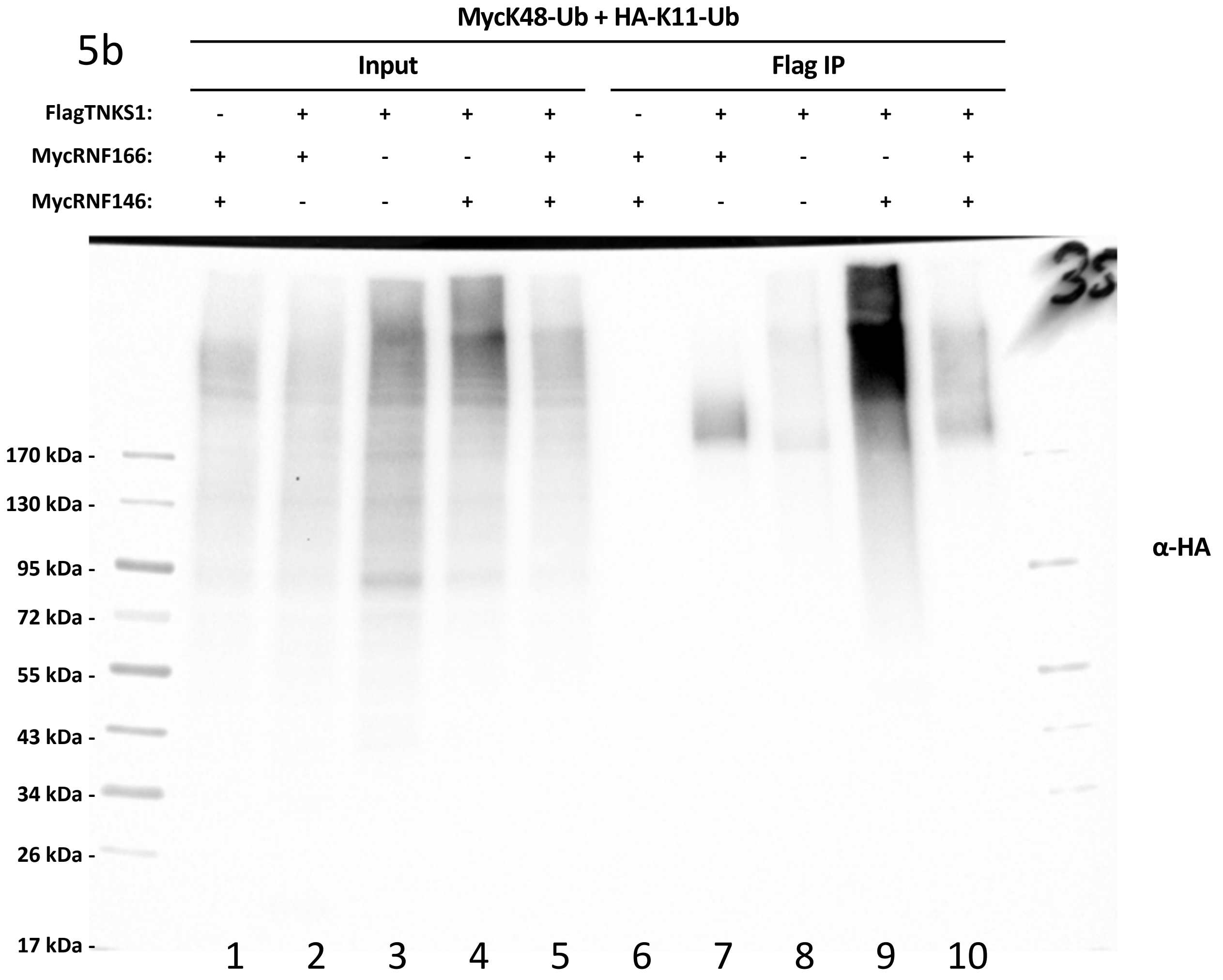
MycK48-Ub + HA-K11-Ub
Input
Flag IP
FlagTNKS1: - + + + + - + + + +
MycRNF166: + + - - + + + - - +
MycRNF146: + - - + + + - - + +
170 kDa -
130 kDa -
95 kDa -
72 kDa -
55 kDa -
43 kDa -
34 kDa -
26 kDa -
17 kDa -
α-HA
1 2 3 4 5 6 7 8 9 10

5b

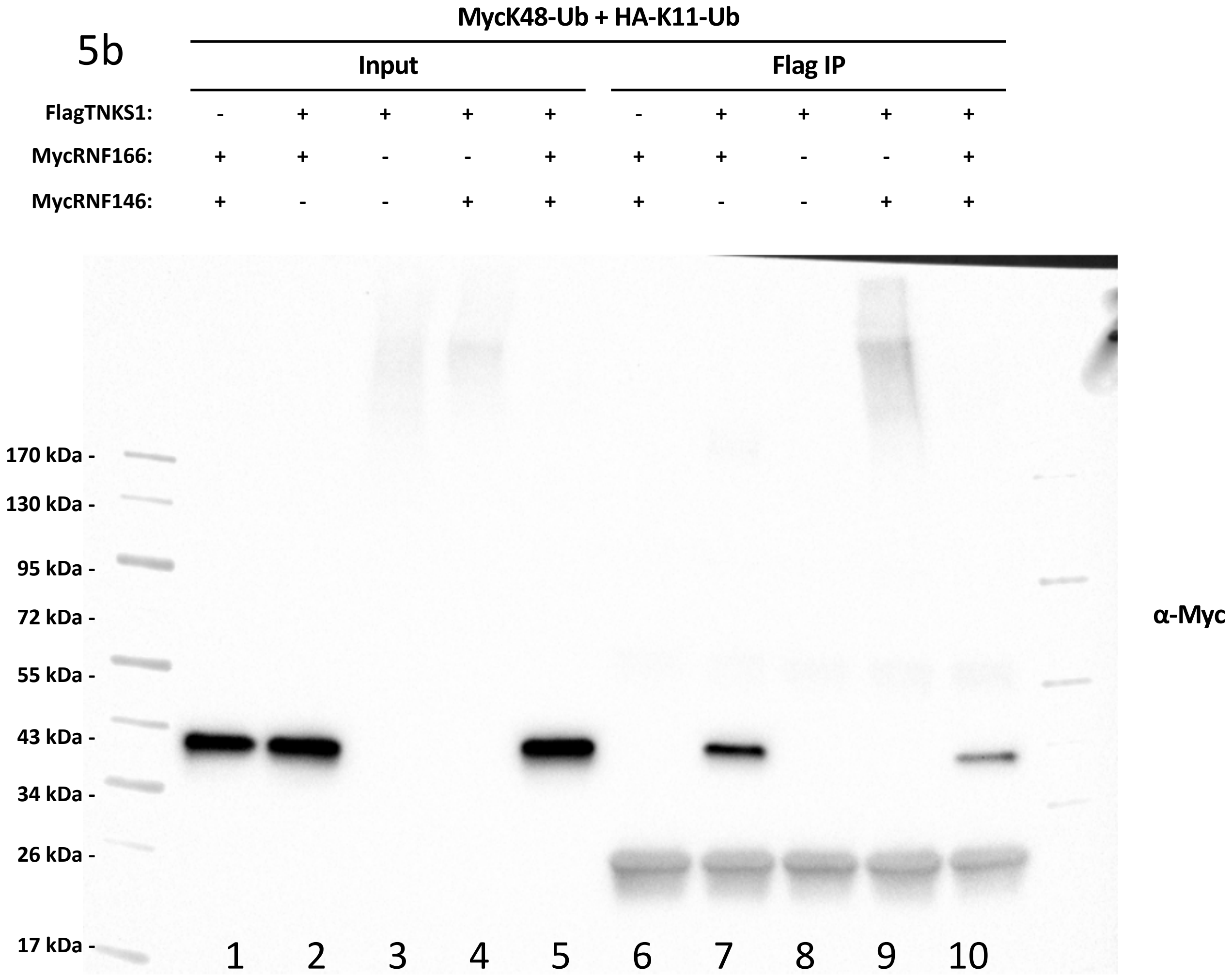
MycK48-Ub + HA-K11-Ub
Input
Flag IP
FlagTNKS1: - + + + + - + + + +
MycRNF166: + + - - + + + - - +
MycRNF146: + - - + + + - - + +
170 kDa -
130 kDa -
95 kDa -
72 kDa -
55 kDa -
43 kDa -
34 kDa -
26 kDa -
17 kDa -
α-Myc
1 2 3 4 5 6 7 8 9 10

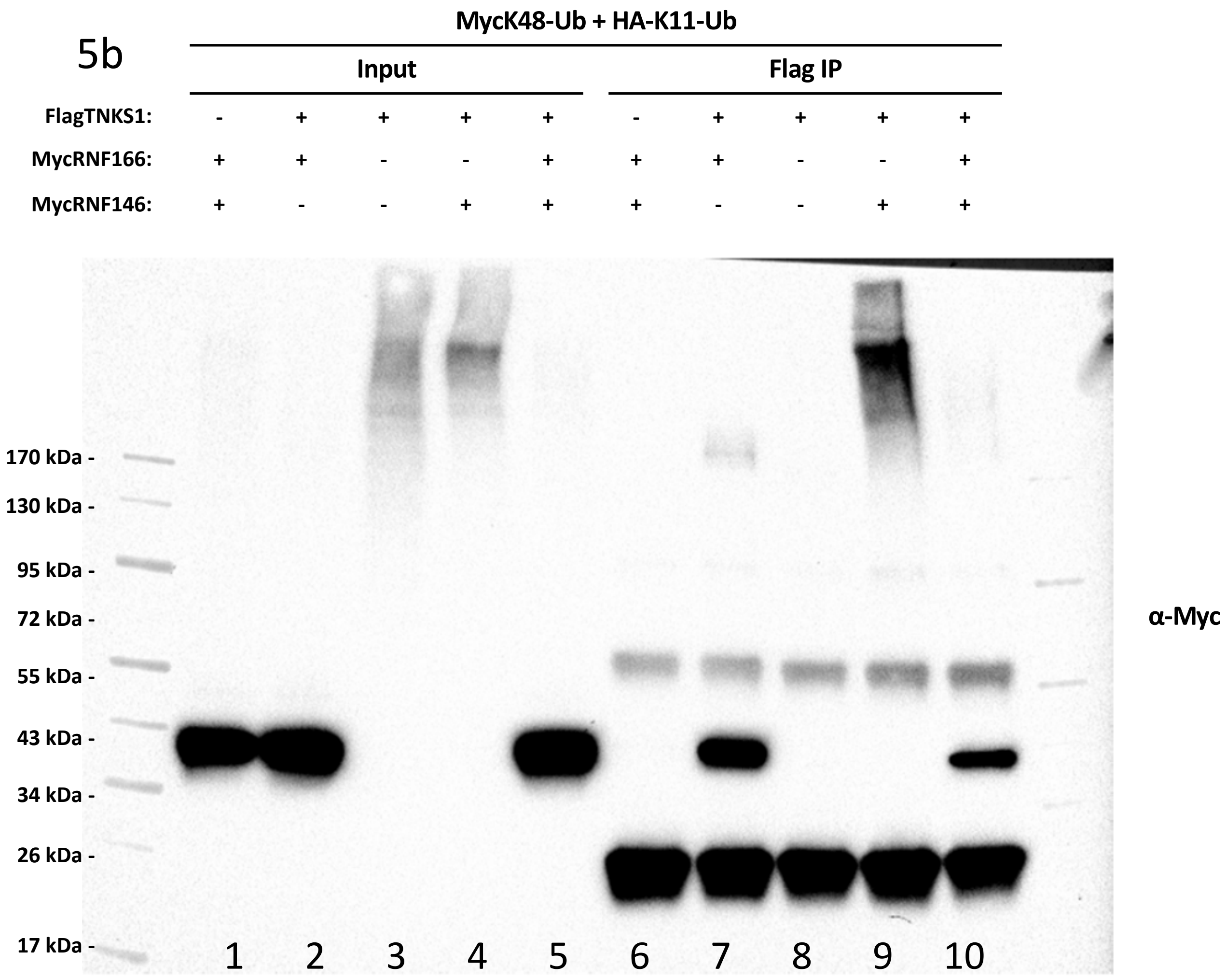
5b
MycK48-Ub + HA-K11-Ub
Input
Flag IP
FlagTNKS1: - + + + + - + + + +
MycRNF166: + + - - + + + - - +
MycRNF146: + - - + + + - - + +
170 kDa -
130 kDa -
95 kDa -
72 kDa -
55 kDa -
43 kDa -
34 kDa -
26 kDa -
17 kDa -
1 2 3 4 5 6 7 8 9 10
α-Myc

5b

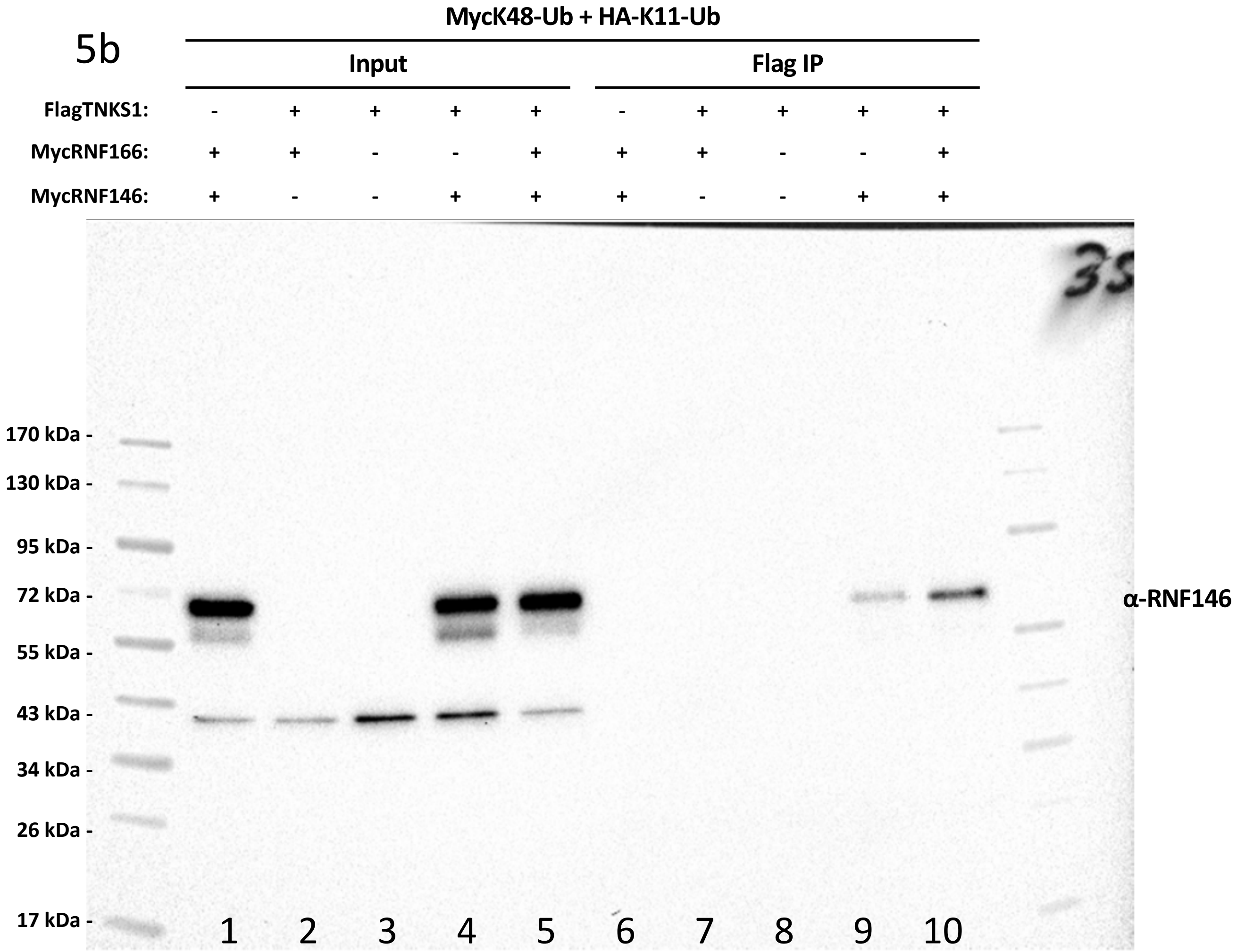
MycK48-Ub + HA-K11-Ub
Input
Flag IP
FlagTNKS1: - + + + + - + + + +
MycRNF166: + + - - + + + - - +
MycRNF146: + - - + + + - - + +
170 kDa -
130 kDa -
95 kDa -
72 kDa -
55 kDa -
43 kDa -
34 kDa -
26 kDa -
17 kDa -
1 2 3 4 5 6 7 8 9 10
α-RNF146

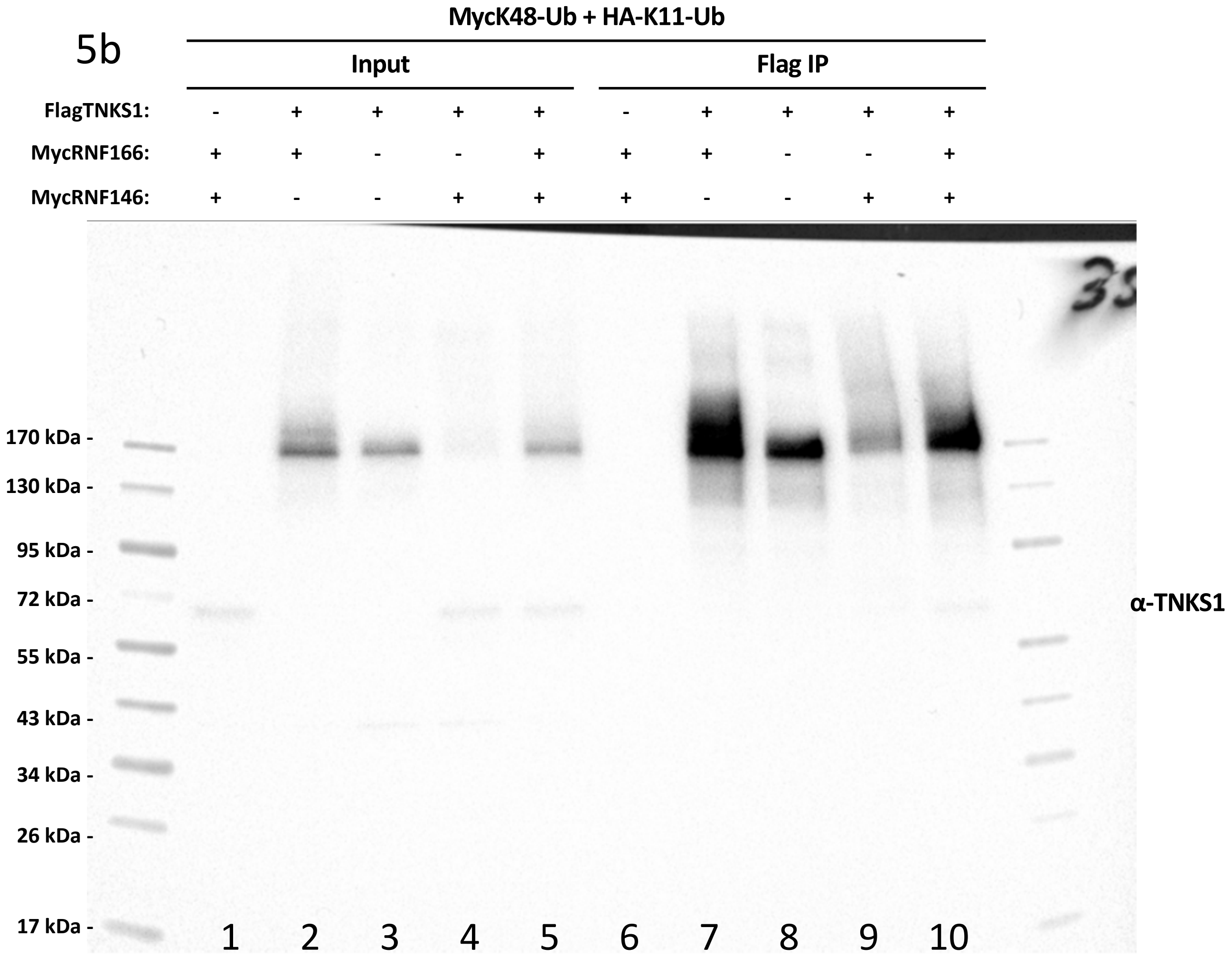
5b
MycK48-Ub + HA-K11-Ub
Input
Flag IP
FlagTNKS1: - + + + + - + + + +
MycRNF166: + + - - + + + - - +
MycRNF146: + - - + + + - - + +
170 kDa -
130 kDa -
95 kDa -
72 kDa -
55 kDa -
43 kDa -
34 kDa -
26 kDa -
17 kDa -
1 2 3 4 5 6 7 8 9 10
α-TNKS1

6b

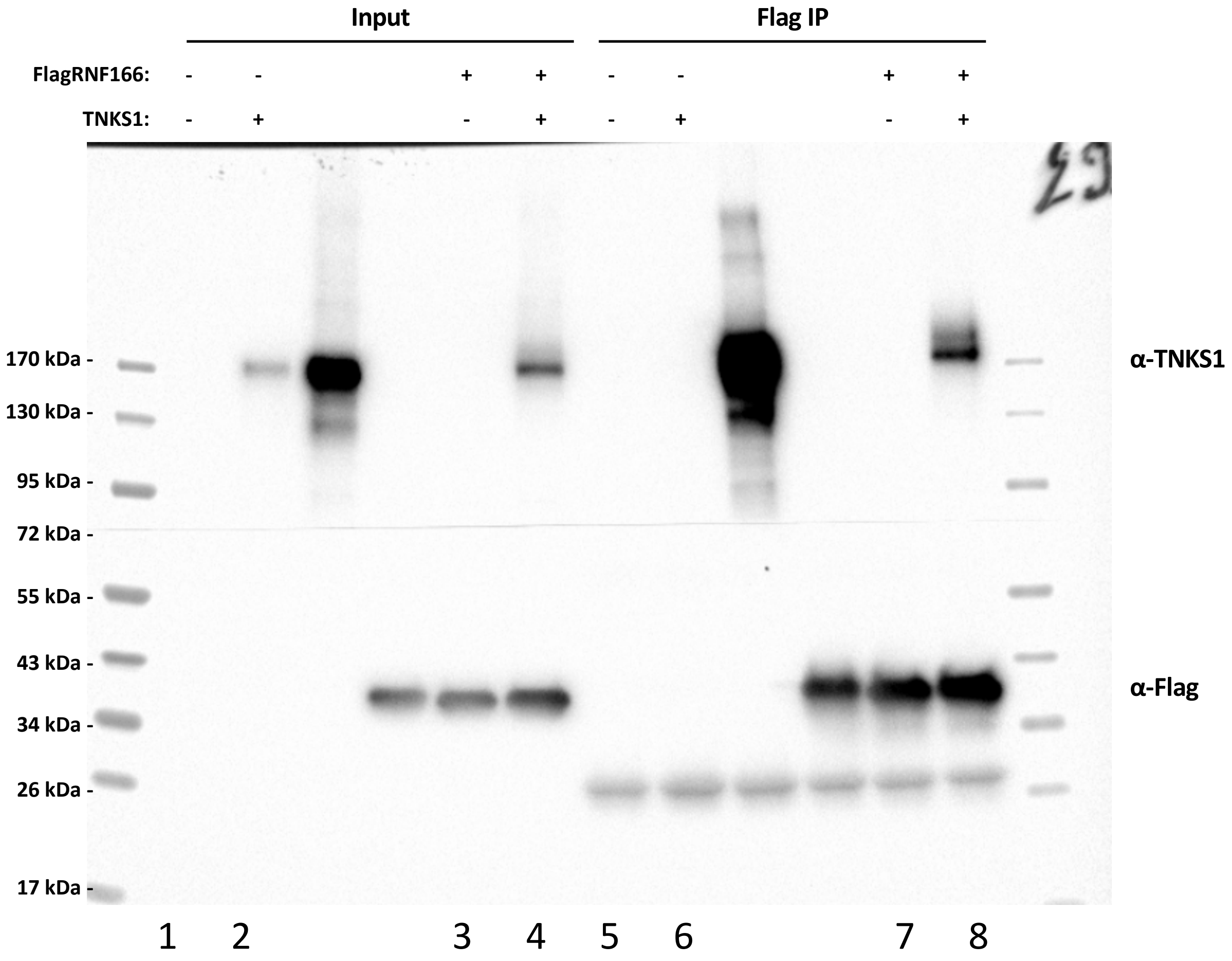

Input
Flag IP
FlagRNF166: - - + + - - + +
TNKS1: - + - + - + - +
170 kDa -
130 kDa -
95 kDa -
72 kDa -
55 kDa -
43 kDa -
34 kDa -
26 kDa -
17 kDa -
α-TNKS1
α-Flag
1 2 3 4 5 6 7 8

6b

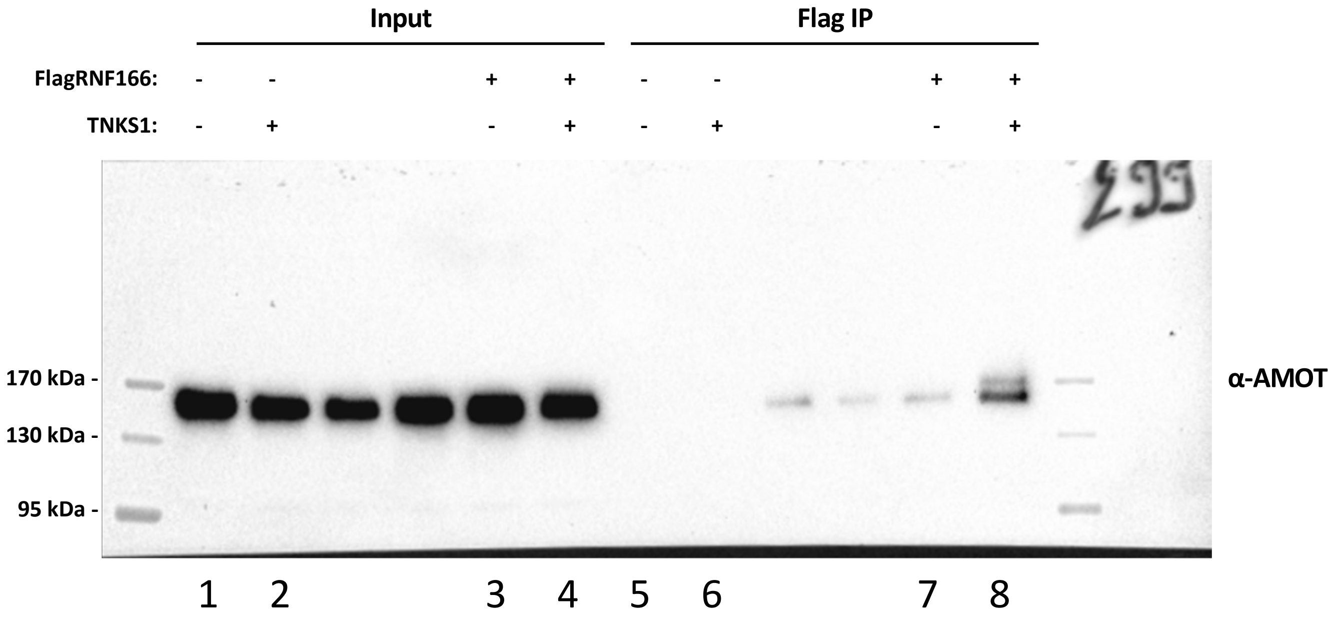

Input
Flag IP
FlagRNF166: - - + + - - + +
TNKS1: - + - + - + - +
170 kDa -
130 kDa -
95 kDa -
α-AMOT
1 2 3 4 5 6 7 8

6c

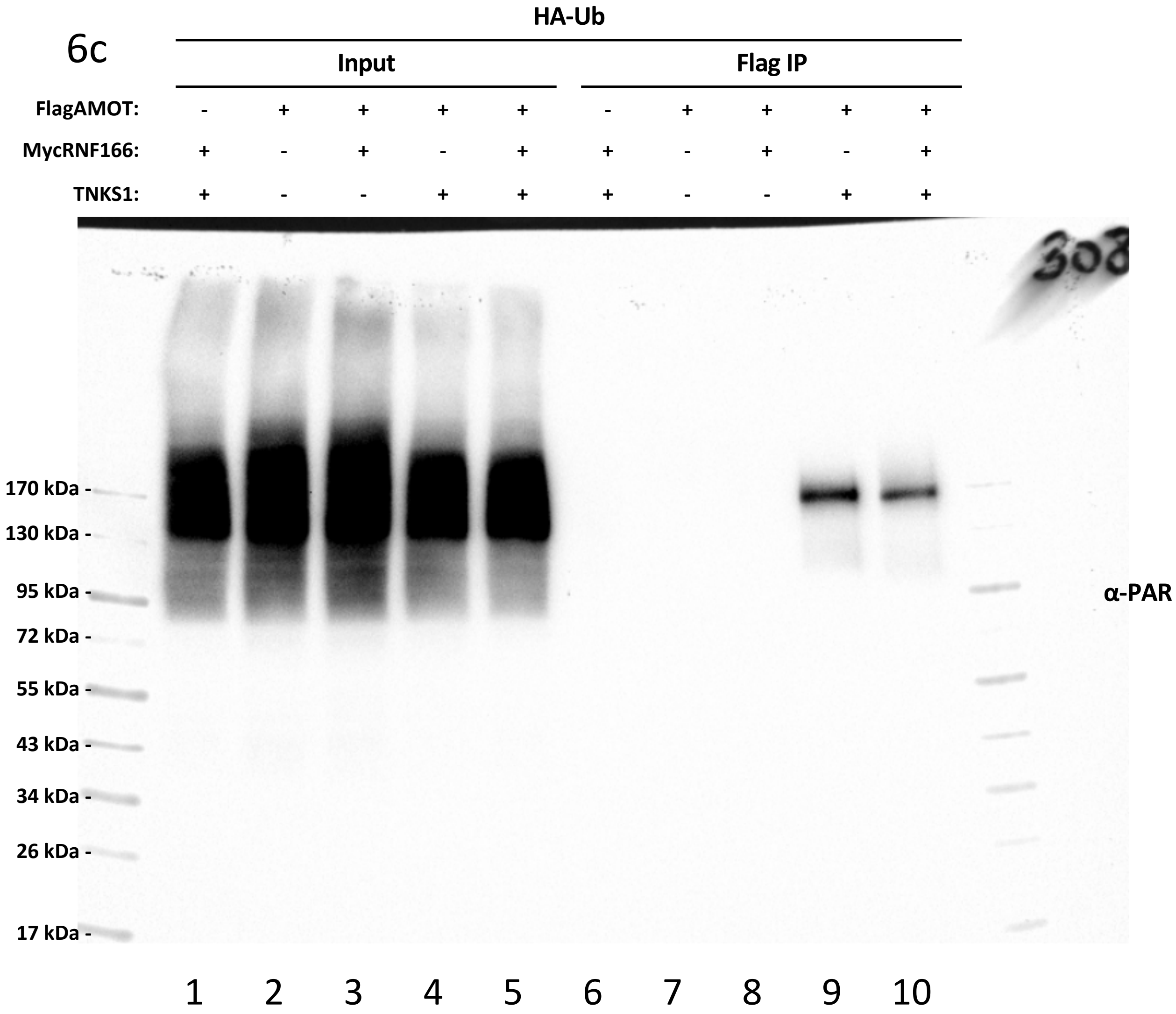

HA-Ub
Input
Flag IP
FlagAMOT: - + + + + - + + + +
MycRNF166: + - + - + + - + - +
TNKS1: + - - + + + - - + +
170 kDa -
130 kDa -
95 kDa -
72 kDa -
55 kDa -
43 kDa -
34 kDa -
26 kDa -
17 kDa -
α-PAR
1 2 3 4 5 6 7 8 9 10

6c

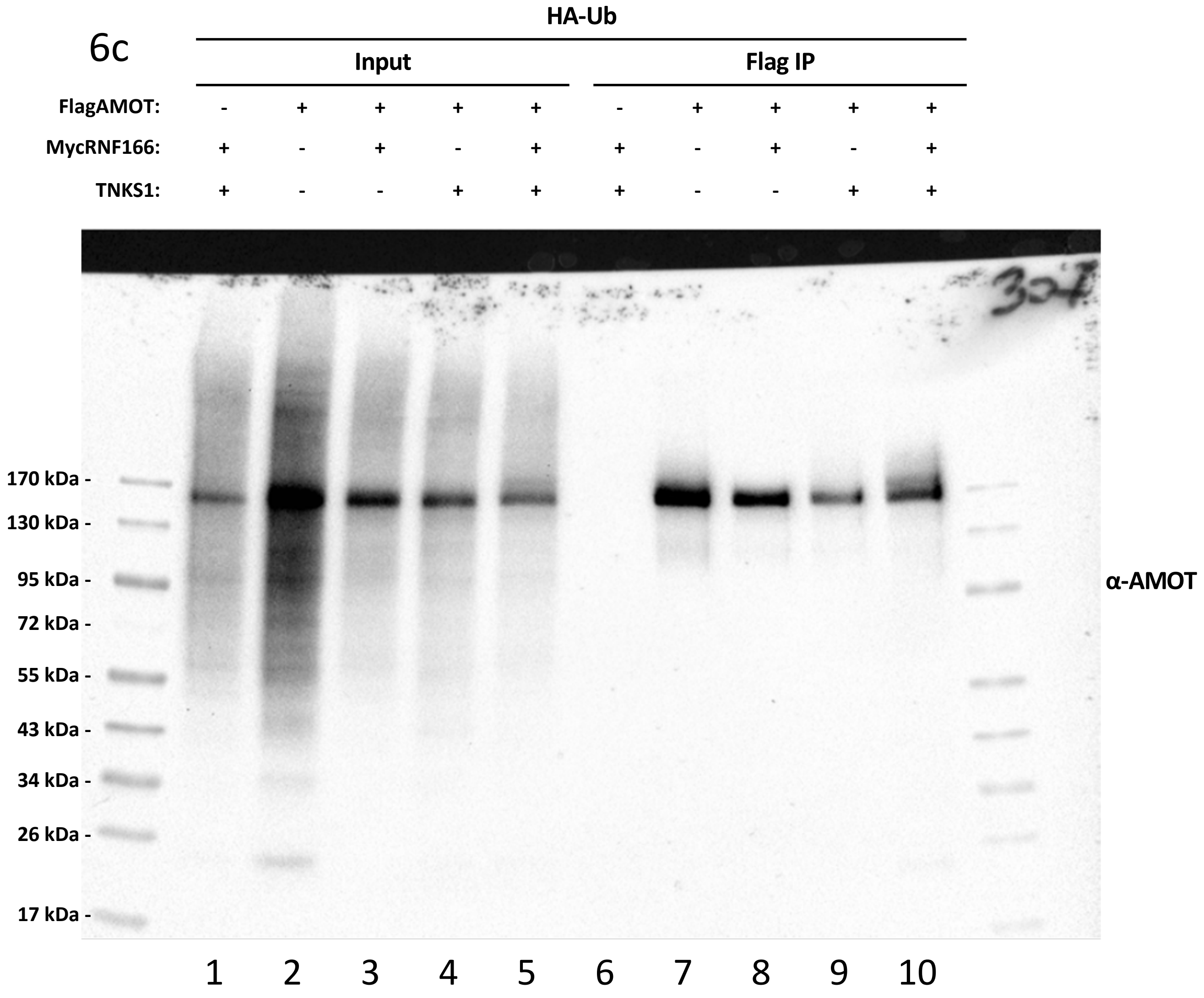
HA-Ub
Input
Flag IP
FlagAMOT: - + + + + - + + + +
MycRNF166: + - + - + + - + - +
TNKS1: + - - + + + - - + +
170 kDa -
130 kDa -
95 kDa -
72 kDa -
55 kDa -
43 kDa -
34 kDa -
26 kDa -
17 kDa -
α-AMOT
1 2 3 4 5 6 7 8 9 10

6c

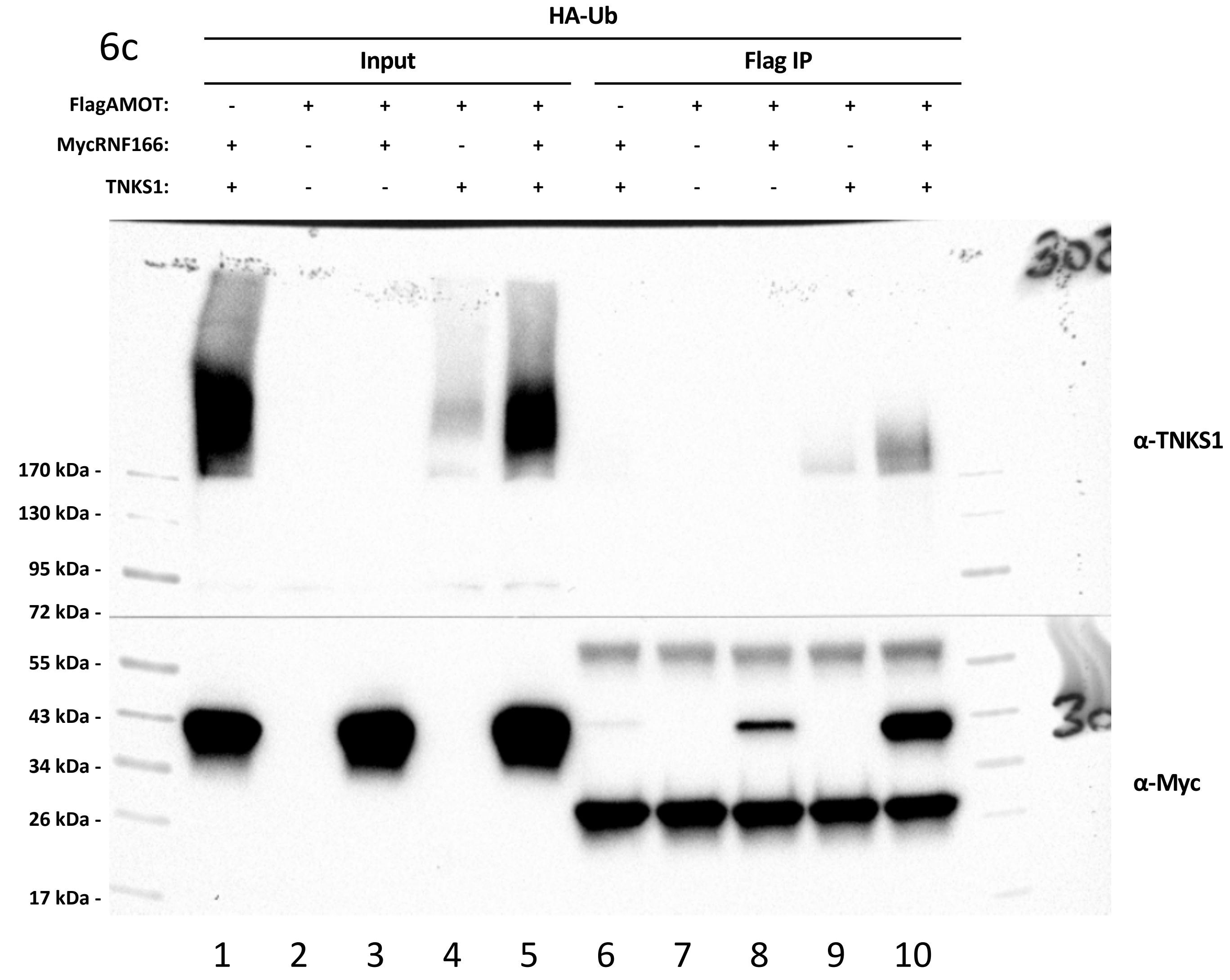
HA-Ub
Input
Flag IP
FlagAMOT: - + + + + - + + + +
MycRNF166: + - + - + + - + - +
TNKS1: + - - + + + - - + +
170 kDa -
130 kDa -
95 kDa -
72 kDa -
55 kDa -
43 kDa -
34 kDa -
26 kDa -
17 kDa -
α-TNKS1
α-Myc
1 2 3 4 5 6 7 8 9 10

6c

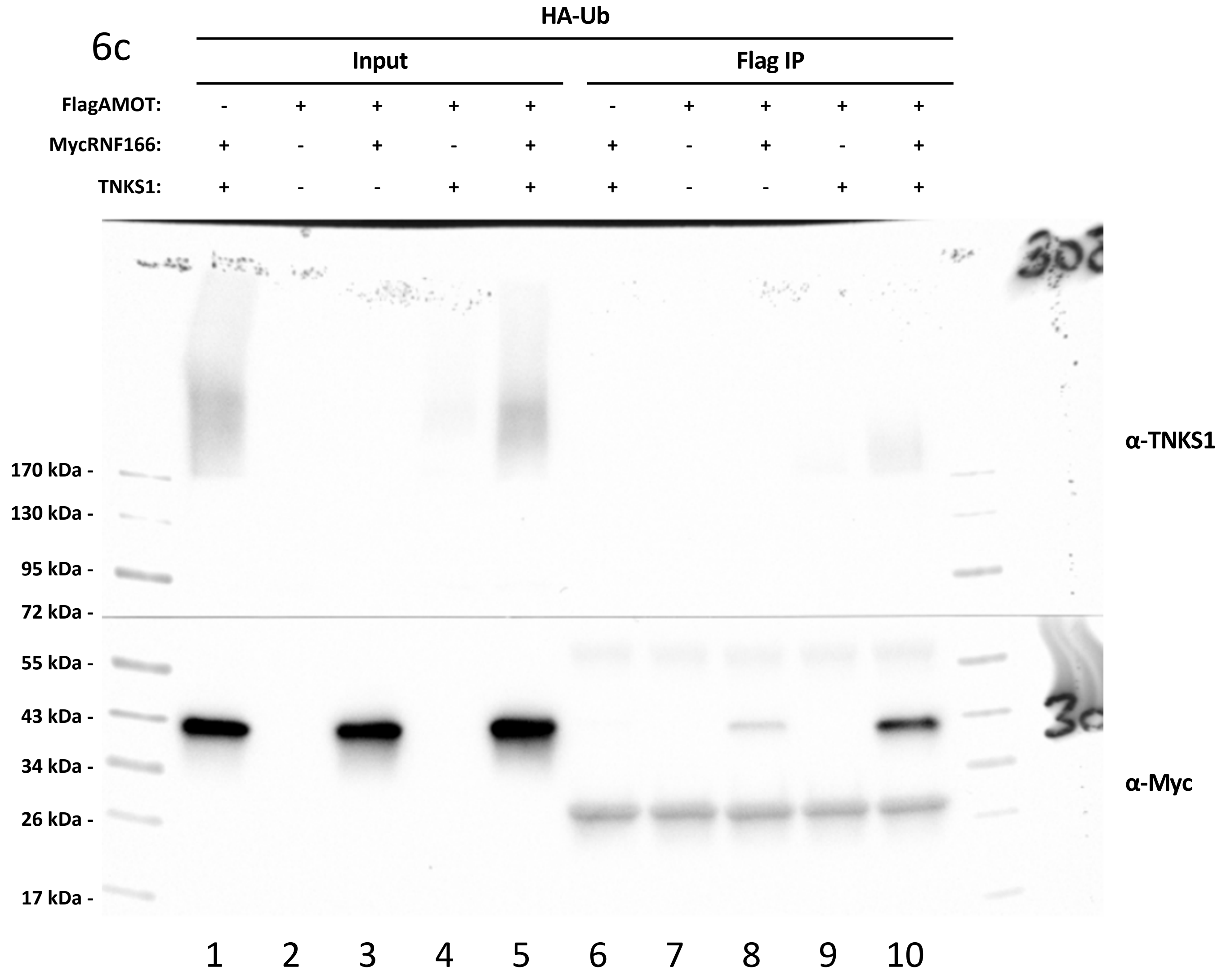

HA-Ub
Input
Flag IP
FlagAMOT: - + + + + - + + + +
MycRNF166: + - + - + + - + - +
TNKS1: + - - + + + - - + +
170 kDa -
130 kDa -
95 kDa -
72 kDa -
55 kDa -
43 kDa -
34 kDa -
26 kDa -
17 kDa -
α-TNKS1
α-Myc
1 2 3 4 5 6 7 8 9 10

6c

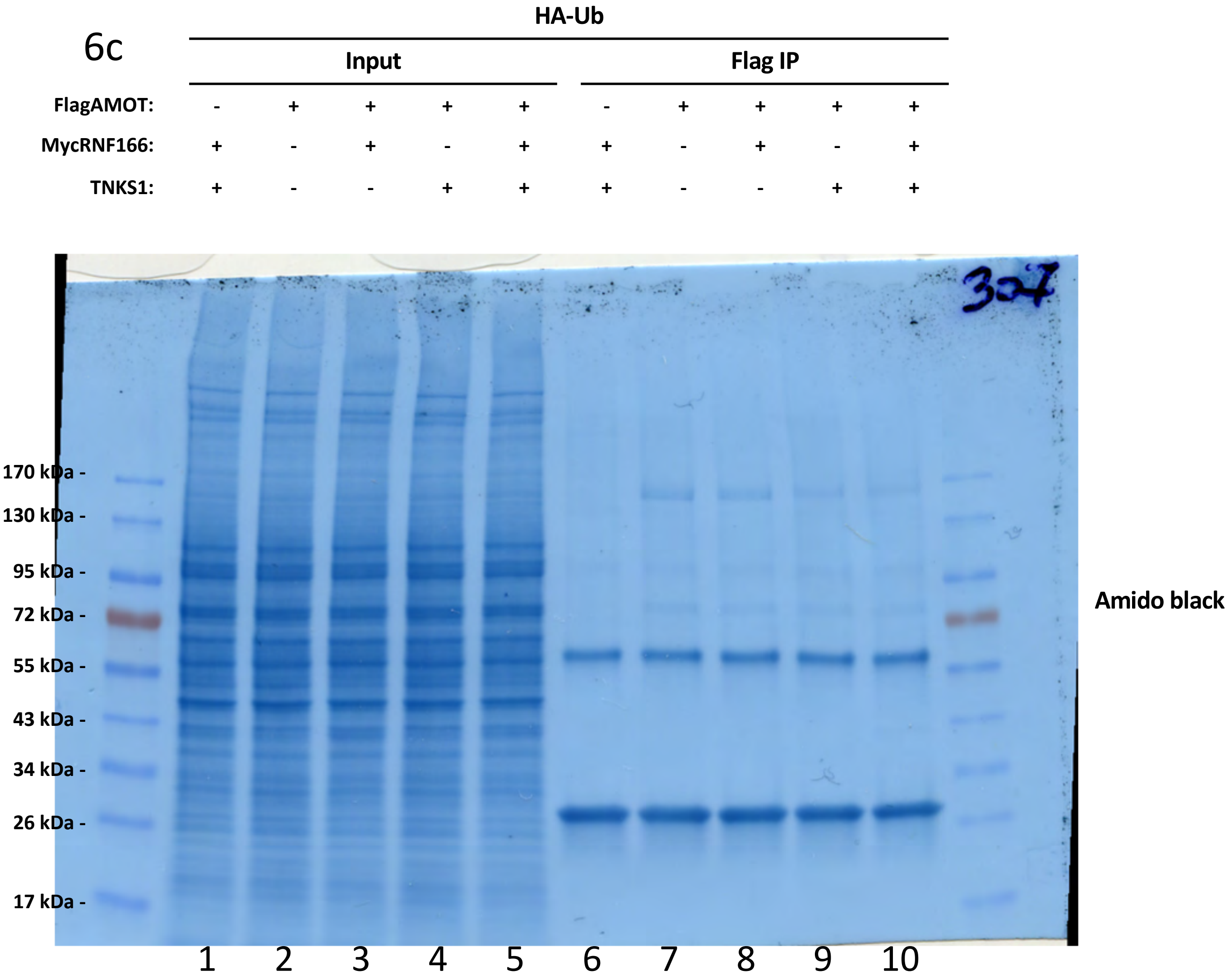
HA-Ub
Input
Flag IP
FlagAMOT: - + + + + - + + + +
MycRNF166: + - + - + + - + - +
TNKS1: + - - + + + - - + +
170 kDa -
130 kDa -
95 kDa -
72 kDa -
55 kDa -
43 kDa -
34 kDa -
26 kDa -
17 kDa -
Amido black
1 2 3 4 5 6 7 8 9 10

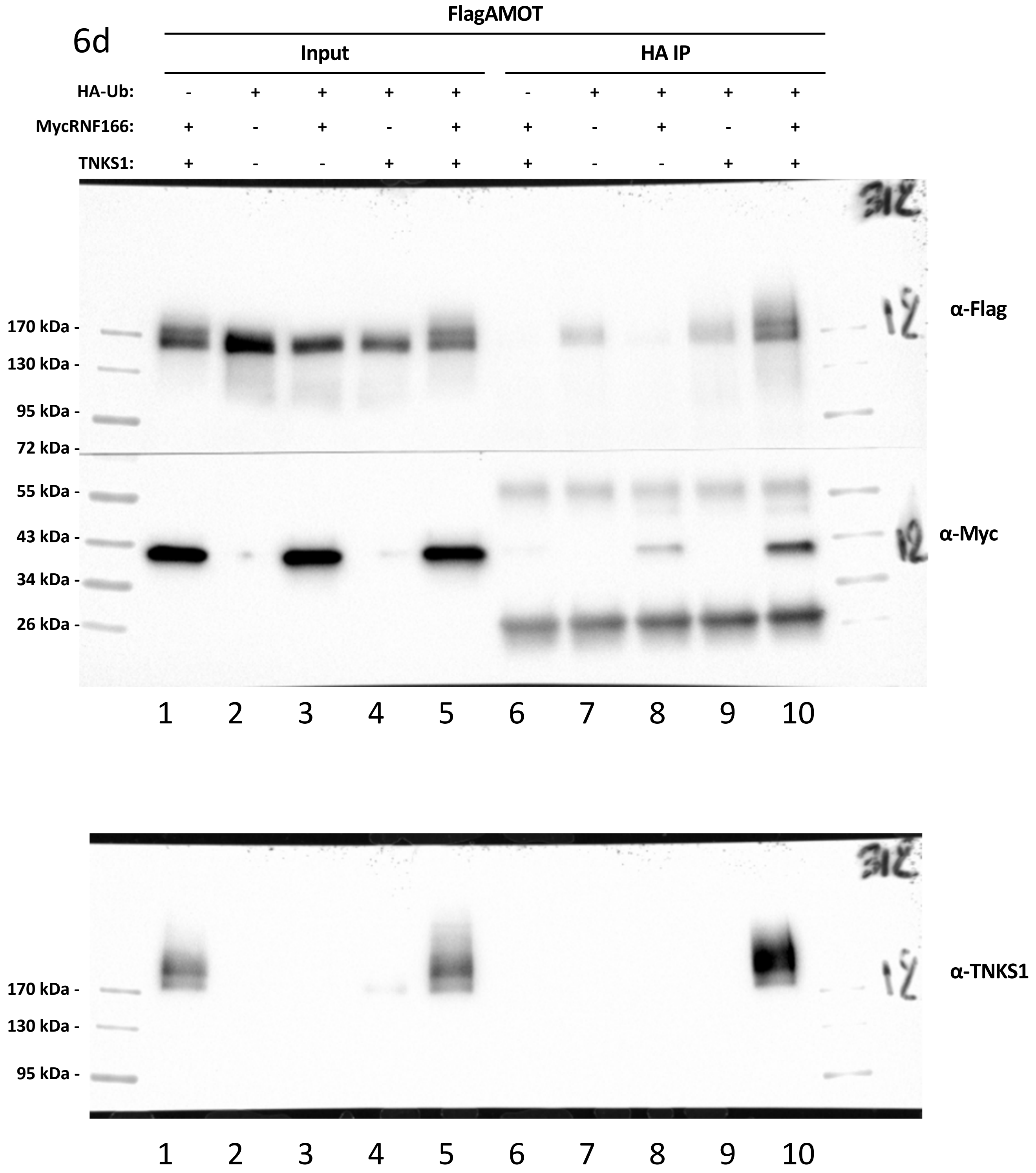
6d
FlagAMOT
Input
HA IP
HA-Ub: - + + + + - + + + +
MycRNF166: + - + - + + - + - +
TNKS1: + - - + + + - - + +
170 kDa -
130 kDa -
95 kDa -
72 kDa -
55 kDa -
43 kDa -
34 kDa -
26 kDa -
α-Flag
α-Myc
1 2 3 4 5 6 7 8 9 10
170 kDa -
130 kDa -
95 kDa -
α-TNKS1
1 2 3 4 5 6 7 8 9 10

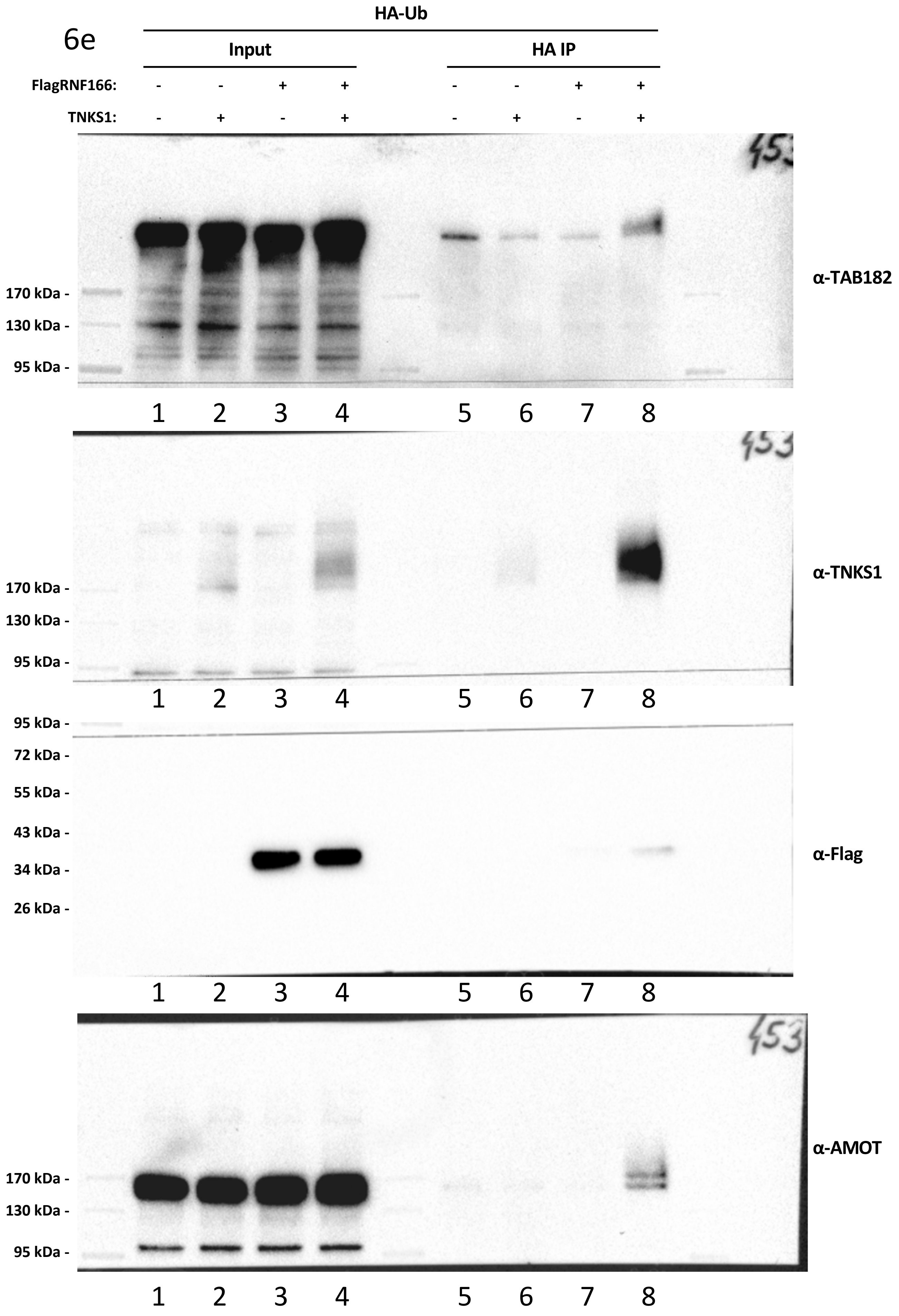
6e
HA-Ub
Input
HA IP
FlagRNF166: - - + + - - + +
TNKS1: - + - + - + - +
170 kDa -
130 kDa -
95 kDa -
α-TAB182
1 2 3 4 5 6 7 8
170 kDa -
130 kDa -
95 kDa -
α-TNKS1
1 2 3 4 5 6 7 8
95 kDa -
72 kDa -
55 kDa -
43 kDa -
34 kDa -
26 kDa -
α-Flag
1 2 3 4 5 6 7 8
170 kDa -
130 kDa -
95 kDa -
α-AMOT
1 2 3 4 5 6 7 8

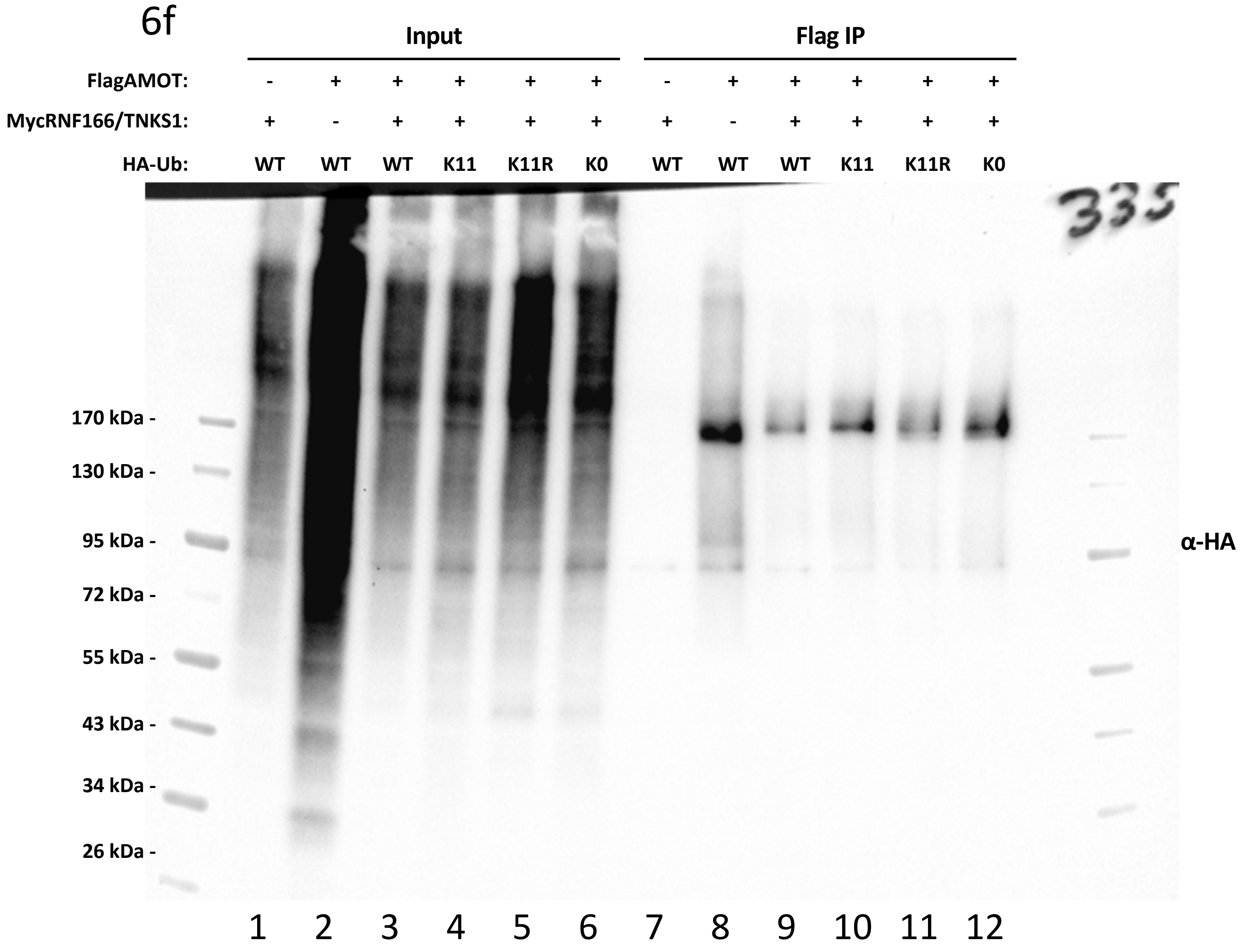
6f
Input
Flag IP
FlagAMOT: - + + + + + - + + + + +
MycRNF166/TNKS1: + - + + + + + - + + + +
HA-Ub: WT WT WT K11 K11R K0 WT WT WT K11 K11R K0
335
170 kDa -
130 kDa -
95 kDa -
72 kDa -
55 kDa -
43 kDa -
34 kDa -
26 kDa -
α-HA
1 2 3 4 5 6 7 8 9 10 11 12

6f

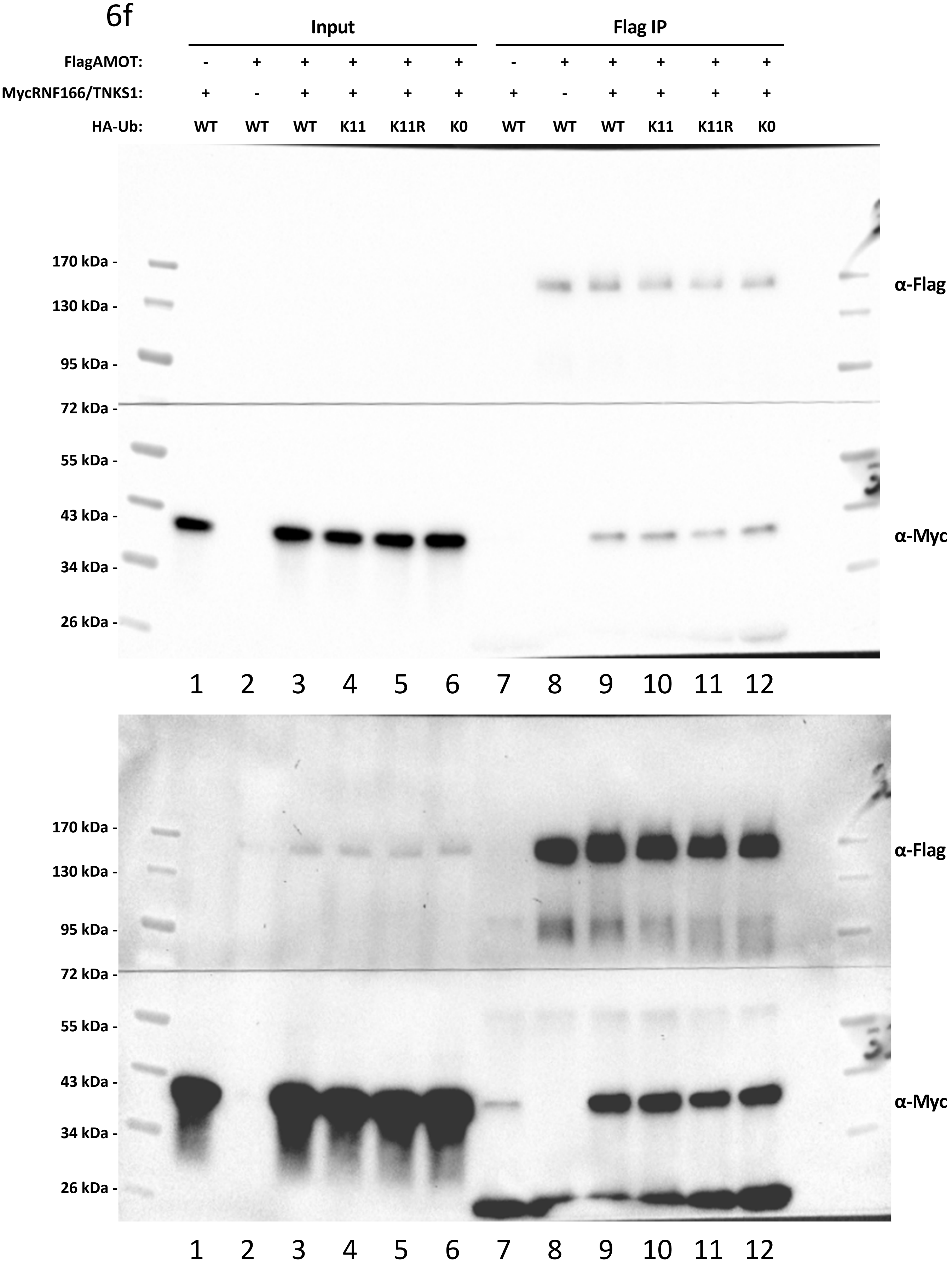

Input
Flag IP
FlagAMOT: - + + + + + - + + + + +
MycRNF166/TNKS1: + - + + + + + - + + + +
HA-Ub: WT WT WT K11 K11R K0 WT WT WT K11 K11R K0
170 kDa -
130 kDa -
95 kDa -
72 kDa -
55 kDa -
43 kDa -
34 kDa -
26 kDa -
α-Flag
α-Myc
1 2 3 4 5 6 7 8 9 10 11 12
170 kDa -
130 kDa -
95 kDa -
72 kDa -
55 kDa -
43 kDa -
34 kDa -
26 kDa -
α-Flag
α-Myc
1 2 3 4 5 6 7 8 9 10 11 12

6g

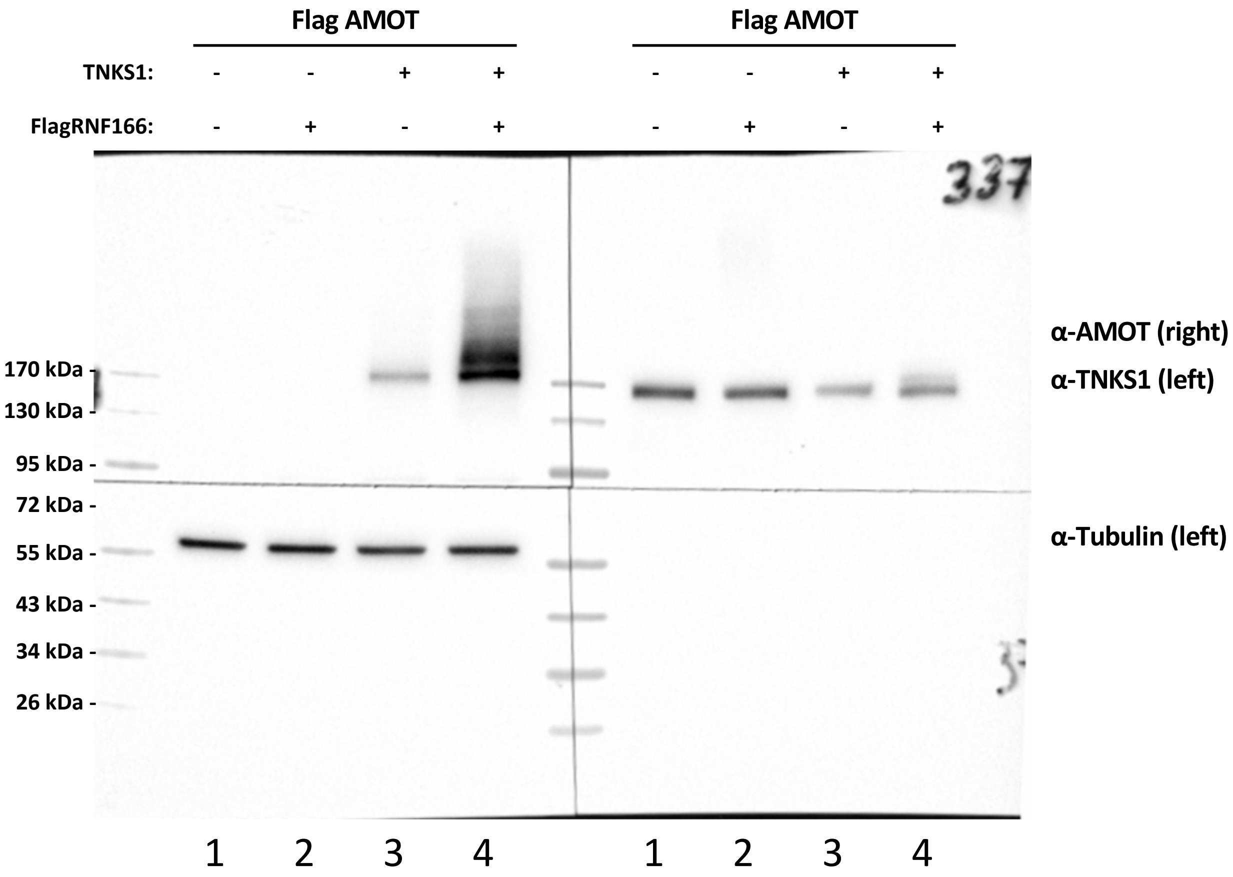
Flag AMOT
Flag AMOT
TNKS1: - - + + - - + +
FlagRNF166: - + - + - + - +
337
α-AMOT (right)
α-TNKS1 (left)
α-Tubulin (left)
170 kDa -
130 kDa -
95 kDa -
72 kDa -
55 kDa -
43 kDa -
34 kDa -
26 kDa -
1 2 3 4 1 2 3 4

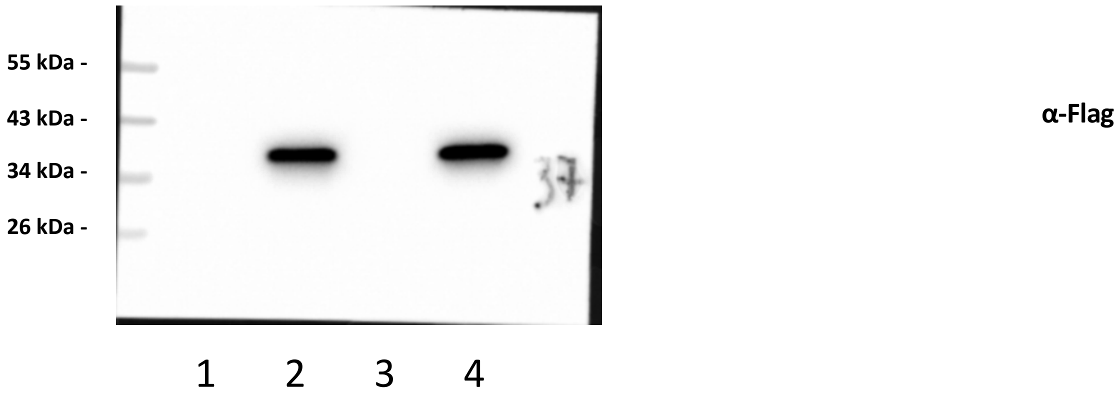
55 kDa -
43 kDa -
34 kDa -
26 kDa -
37
α-Flag
1 2 3 4

7a

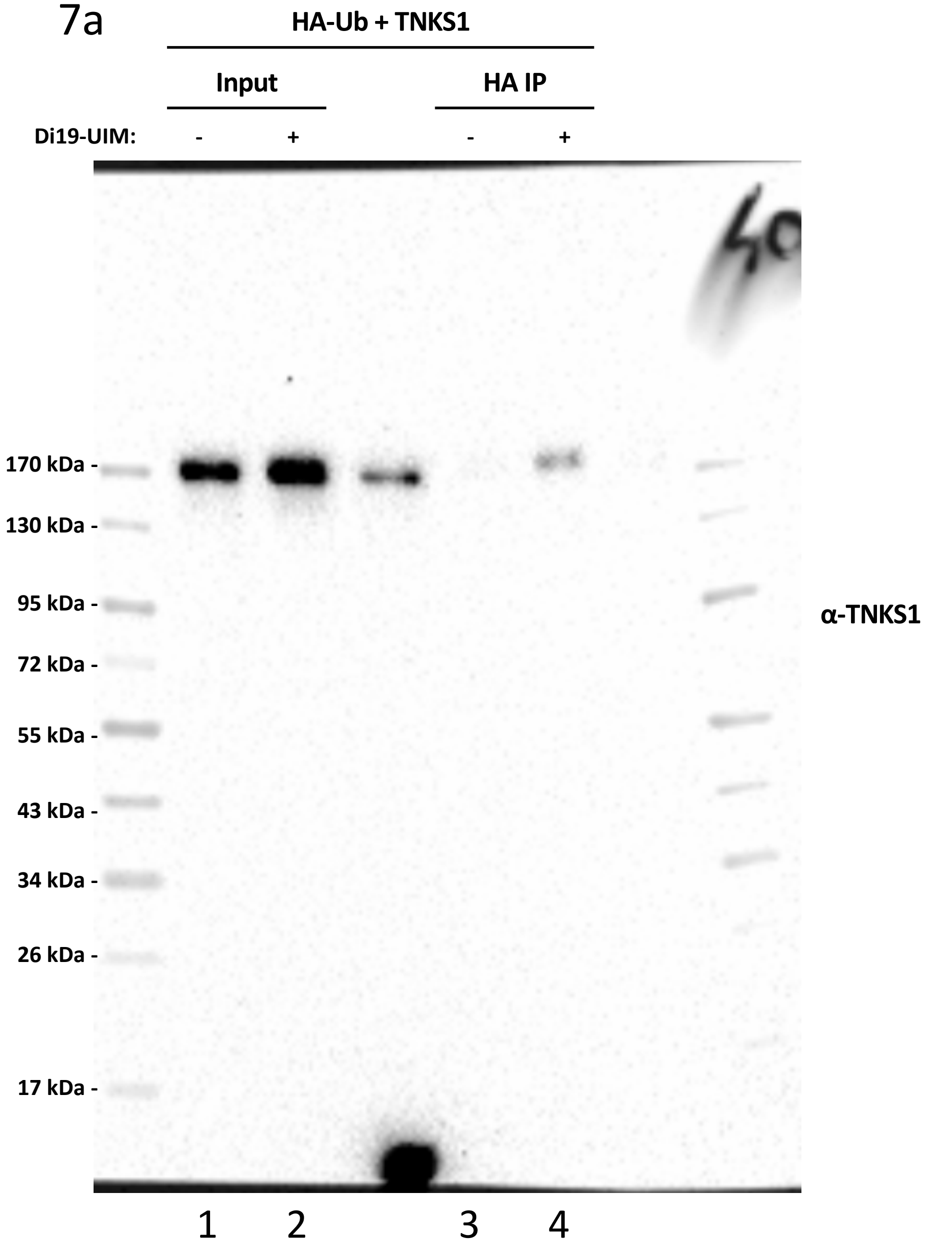

HA-Ub + TNKS1
Input
HA IP
Di19-UIM:
-
+
-
+
170 kDa -
130 kDa -
95 kDa -
72 kDa -
55 kDa -
43 kDa -
34 kDa -
26 kDa -
17 kDa -
α-TNKS1
1
2
3
4

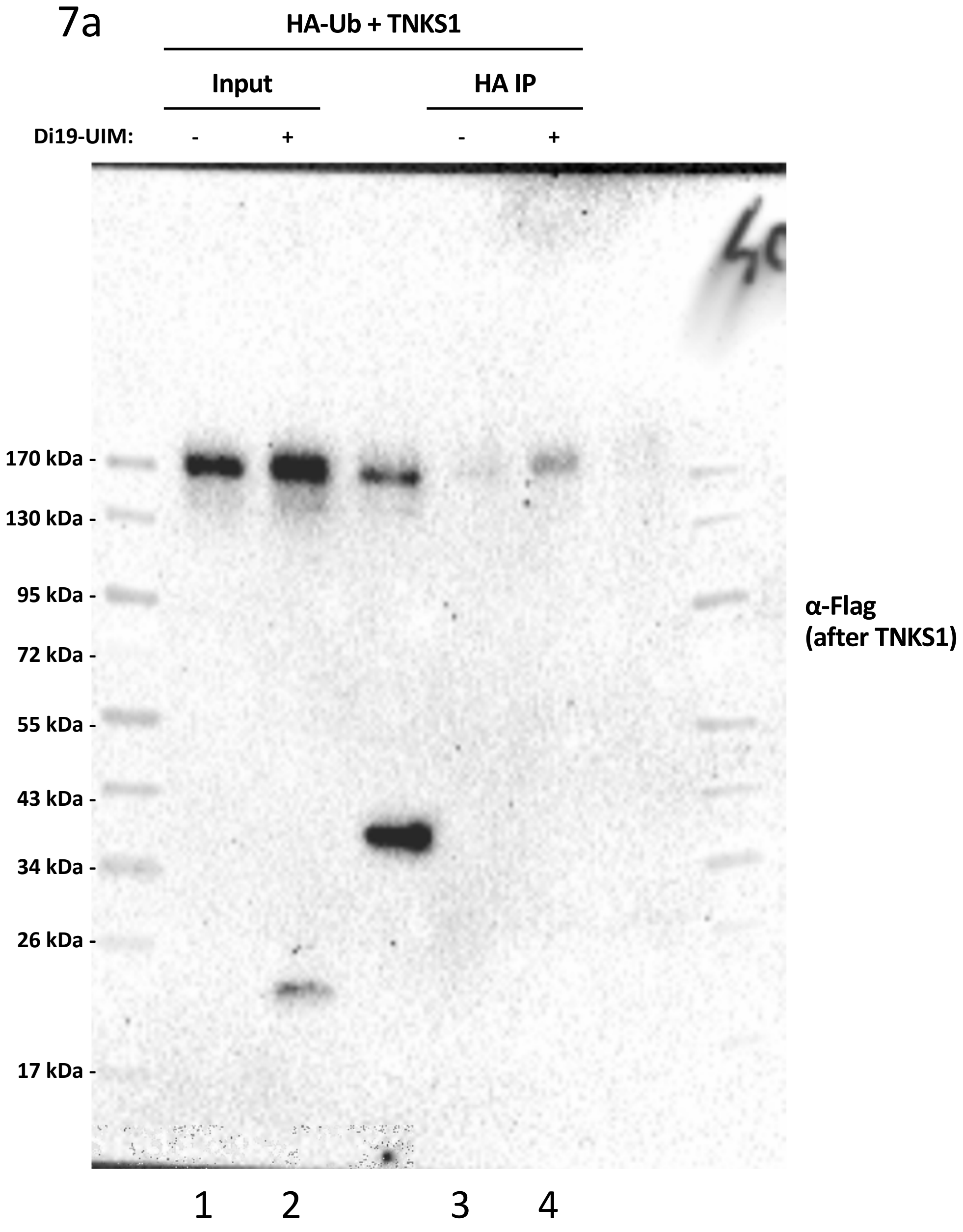
7a
HA-Ub + TNKS1
Input
HA IP
Di19-UIM:
-
+
-
+
170 kDa -
130 kDa -
95 kDa -
72 kDa -
55 kDa -
43 kDa -
34 kDa -
26 kDa -
17 kDa -
α-Flag
(after TNKS1)
1
2
3
4

7c

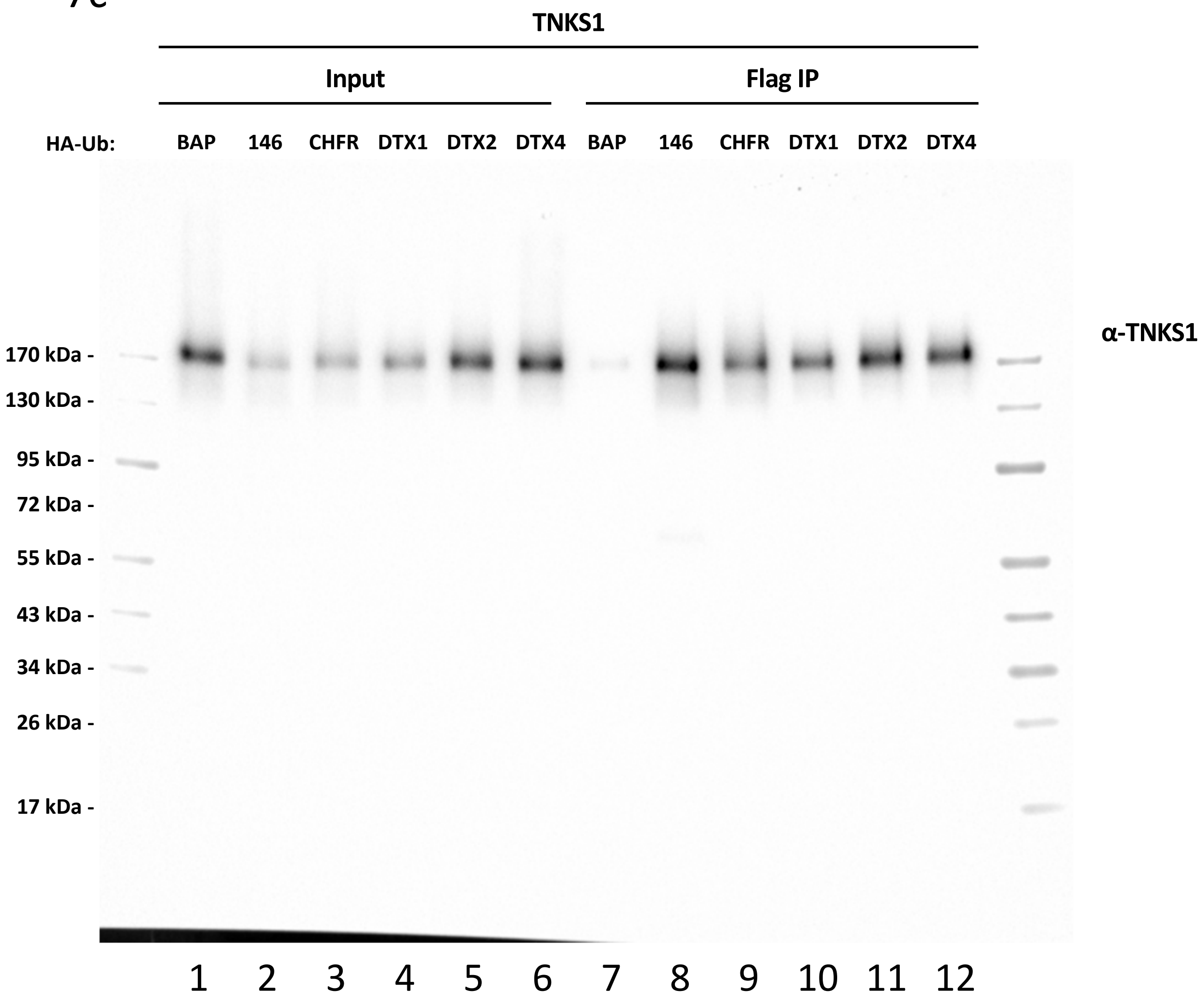
TNKS1
Input
Flag IP
HA-Ub:
BAP
146
CHFR
DTX1
DTX2
DTX4
BAP
146
CHFR
DTX1
DTX2
DTX4
α-TNKS1
170 kDa -
130 kDa -
95 kDa -
72 kDa -
55 kDa -
43 kDa -
34 kDa -
26 kDa -
17 kDa -
1
2
3
4
5
6
7
8
9
10
11
12

7c

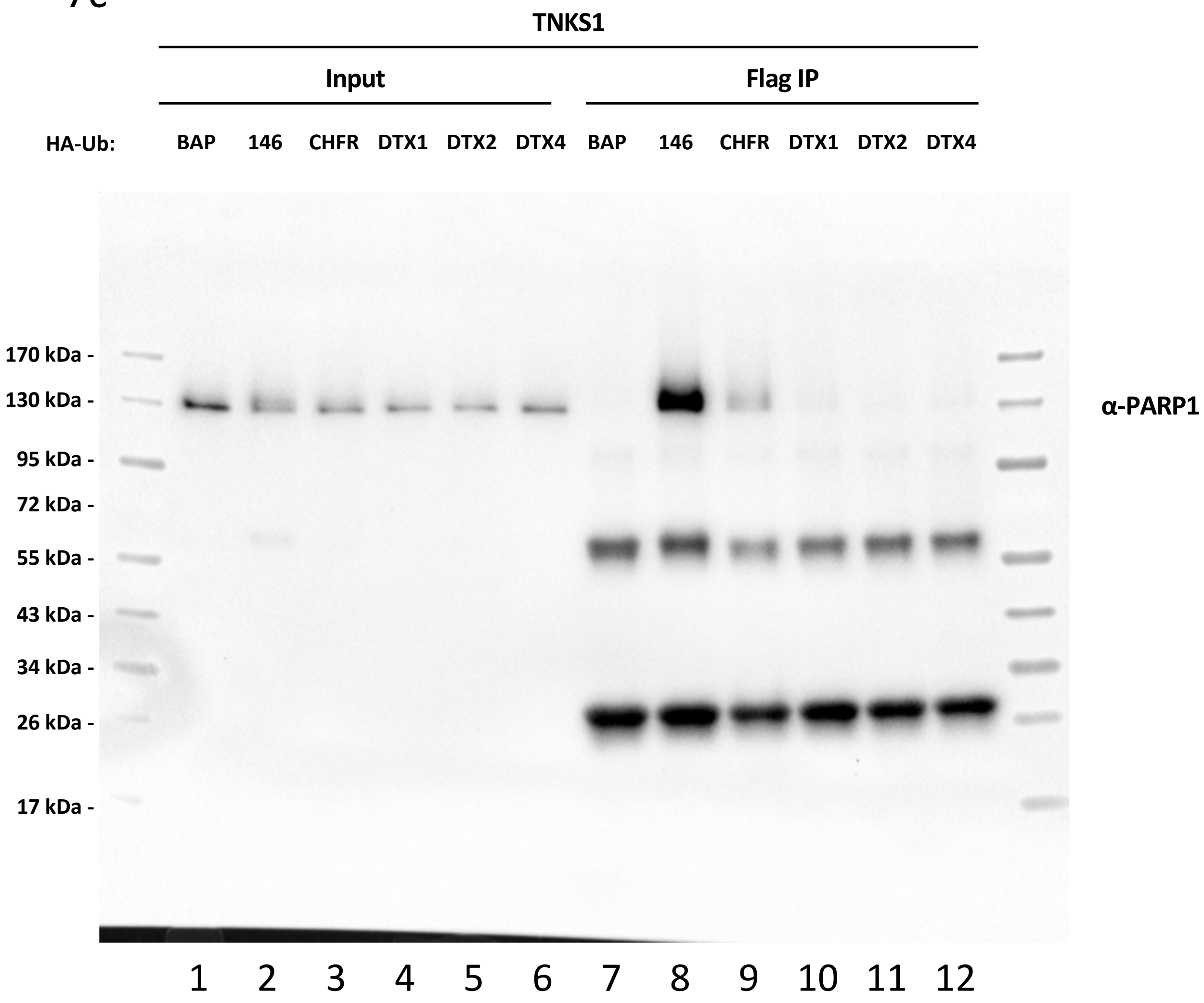

TNKS1
Input
Flag IP
HA-Ub:
BAP
146
CHFR
DTX1
DTX2
DTX4
BAP
146
CHFR
DTX1
DTX2
DTX4
170 kDa -
130 kDa -
95 kDa -
72 kDa -
55 kDa -
43 kDa -
34 kDa -
26 kDa -
17 kDa -
α-PARP1
1
2
3
4
5
6
7
8
9
10
11
12

7c

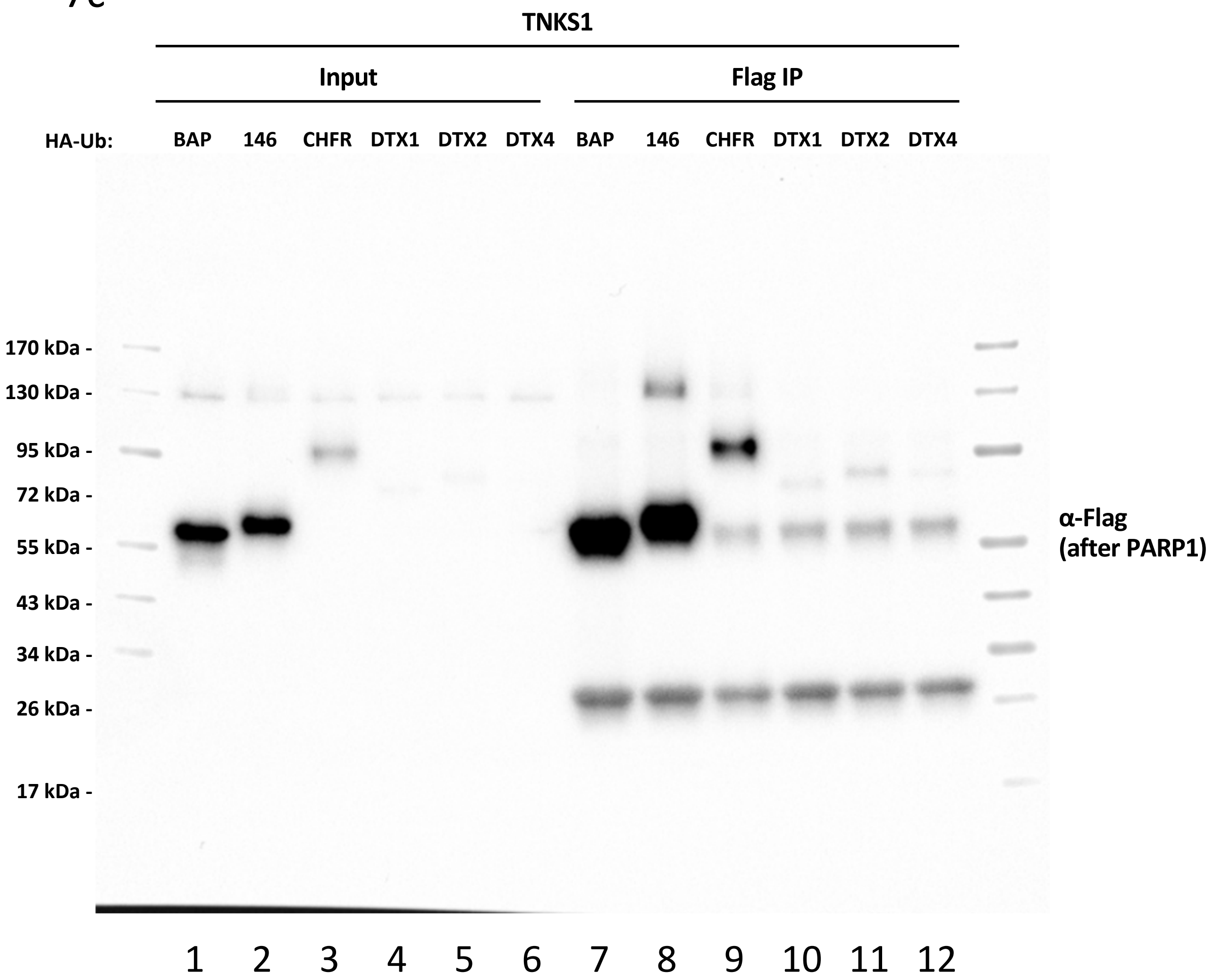
TNKS1
Input
Flag IP
HA-Ub:
BAP
146
CHFR
DTX1
DTX2
DTX4
BAP
146
CHFR
DTX1
DTX2
DTX4
170 kDa -
130 kDa -
95 kDa -
72 kDa -
55 kDa -
43 kDa -
34 kDa -
26 kDa -
17 kDa -
α-Flag
(after PARP1)
1
2
3
4
5
6
7
8
9
10
11
12

7d

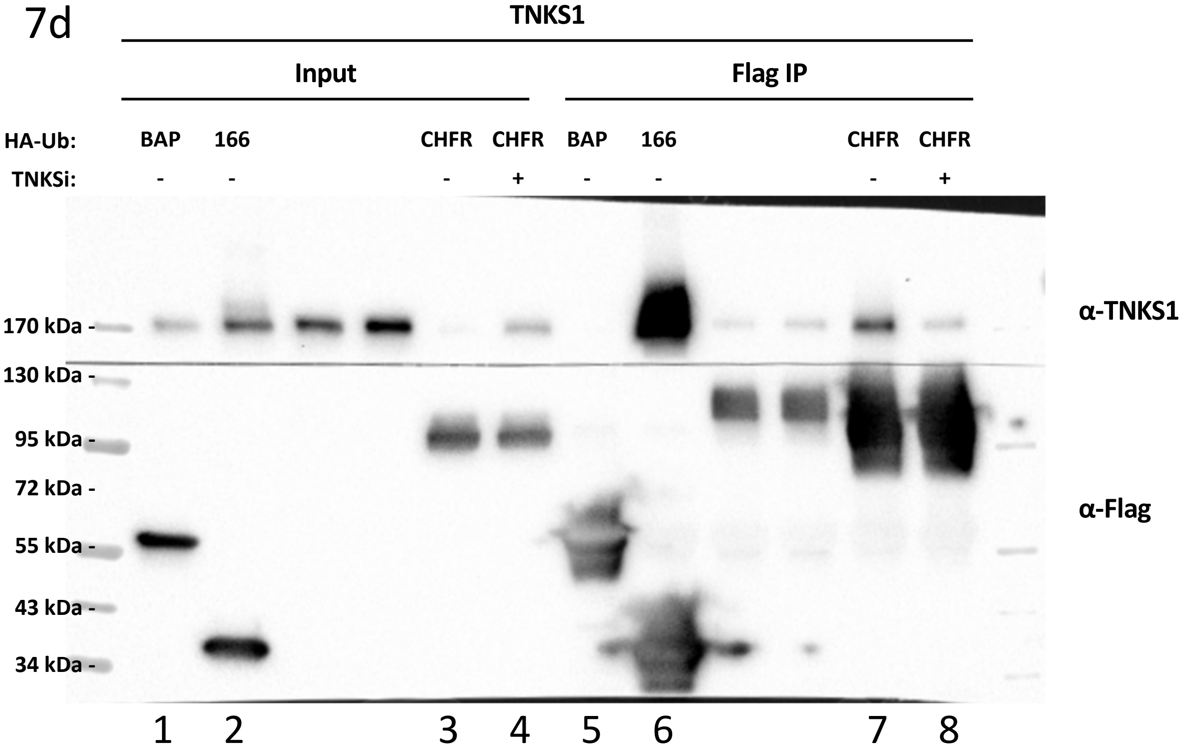
TNKS1
Input
Flag IP
HA-Ub:
BAP
166
CHFR
CHFR
BAP
166
CHFR
CHFR
TNKSi:
-
-
-
+
-
-
-
+
170 kDa -
130 kDa -
95 kDa -
72 kDa -
55 kDa -
43 kDa -
34 kDa -
α-TNKS1
α-Flag
1
2
3
4
5
6
7
8

7e

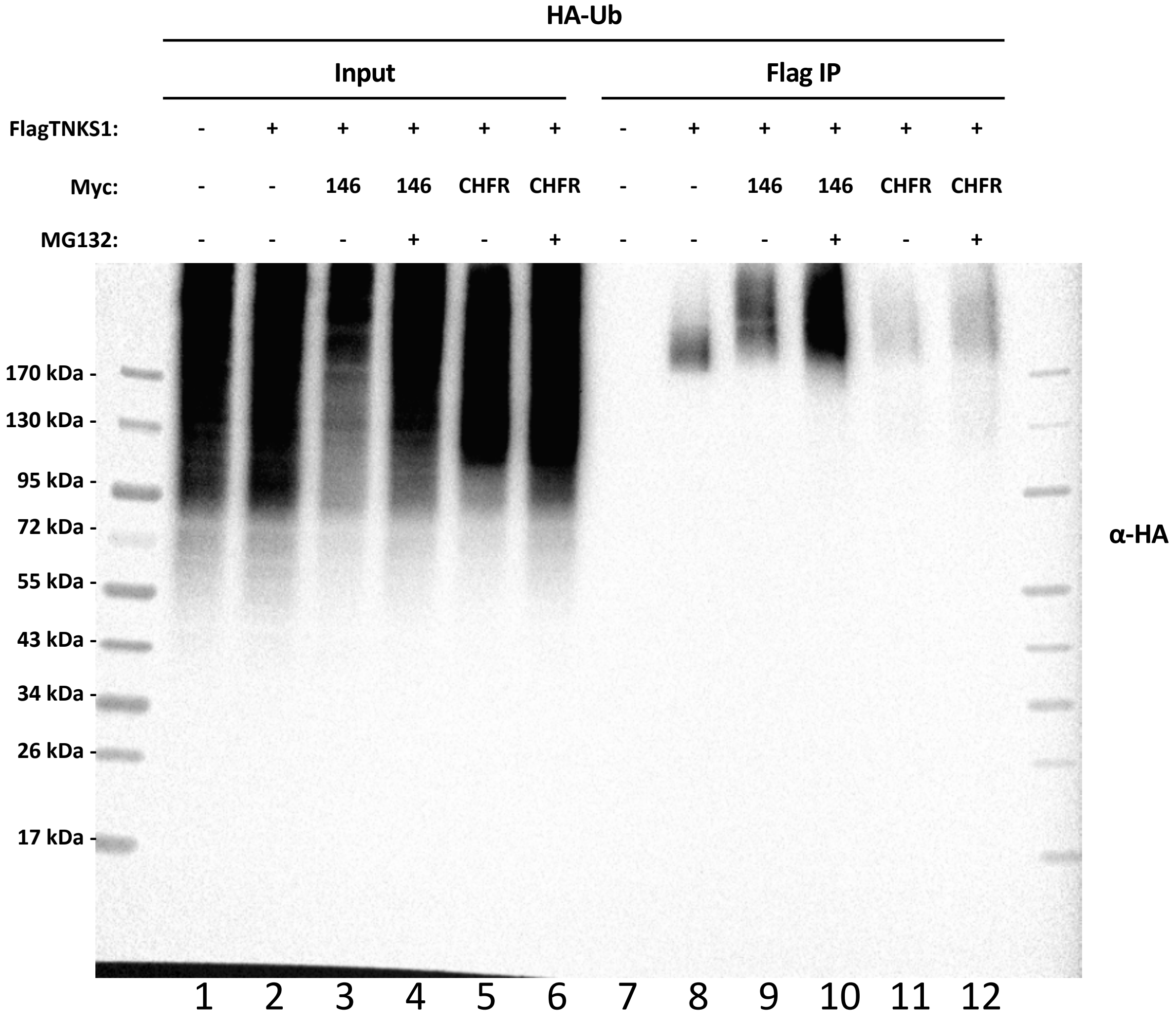
HA-Ub
Input
Flag IP
FlagTNKS1: - + + + + + - + + + + +
Myc: - - 146 146 CHFR CHFR - - 146 146 CHFR CHFR
MG132: - - - + - + - - - + - +
170 kDa -
130 kDa -
95 kDa -
72 kDa -
55 kDa -
43 kDa -
34 kDa -
26 kDa -
17 kDa -
α-HA
1 2 3 4 5 6 7 8 9 10 11 12

7e

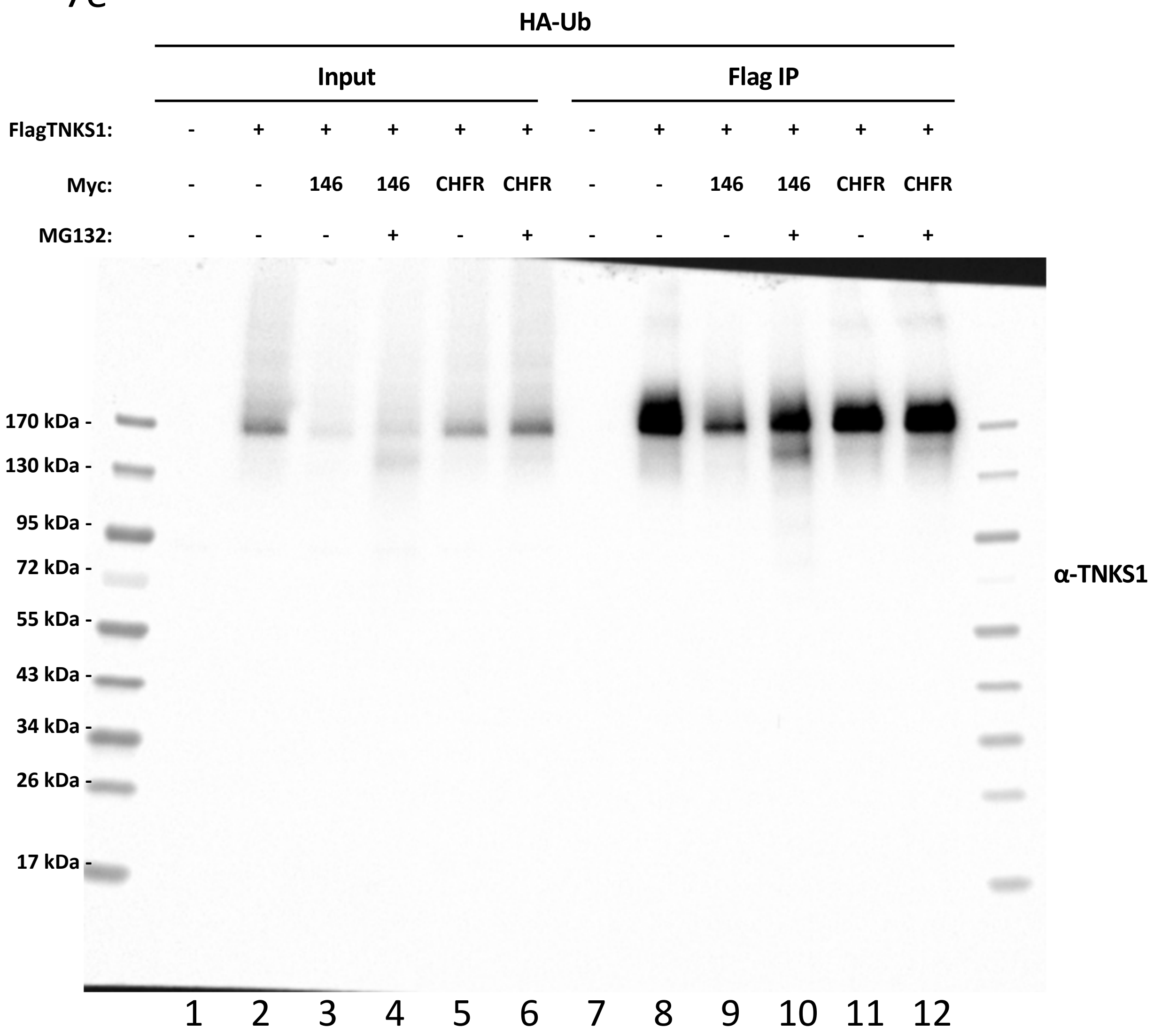
HA-Ub
Input
Flag IP
FlagTNKS1: - + + + + + - + + + + +
Myc: - - 146 146 CHFR CHFR - - 146 146 CHFR CHFR
MG132: - - - + - + - - - + - +
170 kDa -
130 kDa -
95 kDa -
72 kDa -
55 kDa -
43 kDa -
34 kDa -
26 kDa -
17 kDa -
α-TNKS1
1 2 3 4 5 6 7 8 9 10 11 12

7e

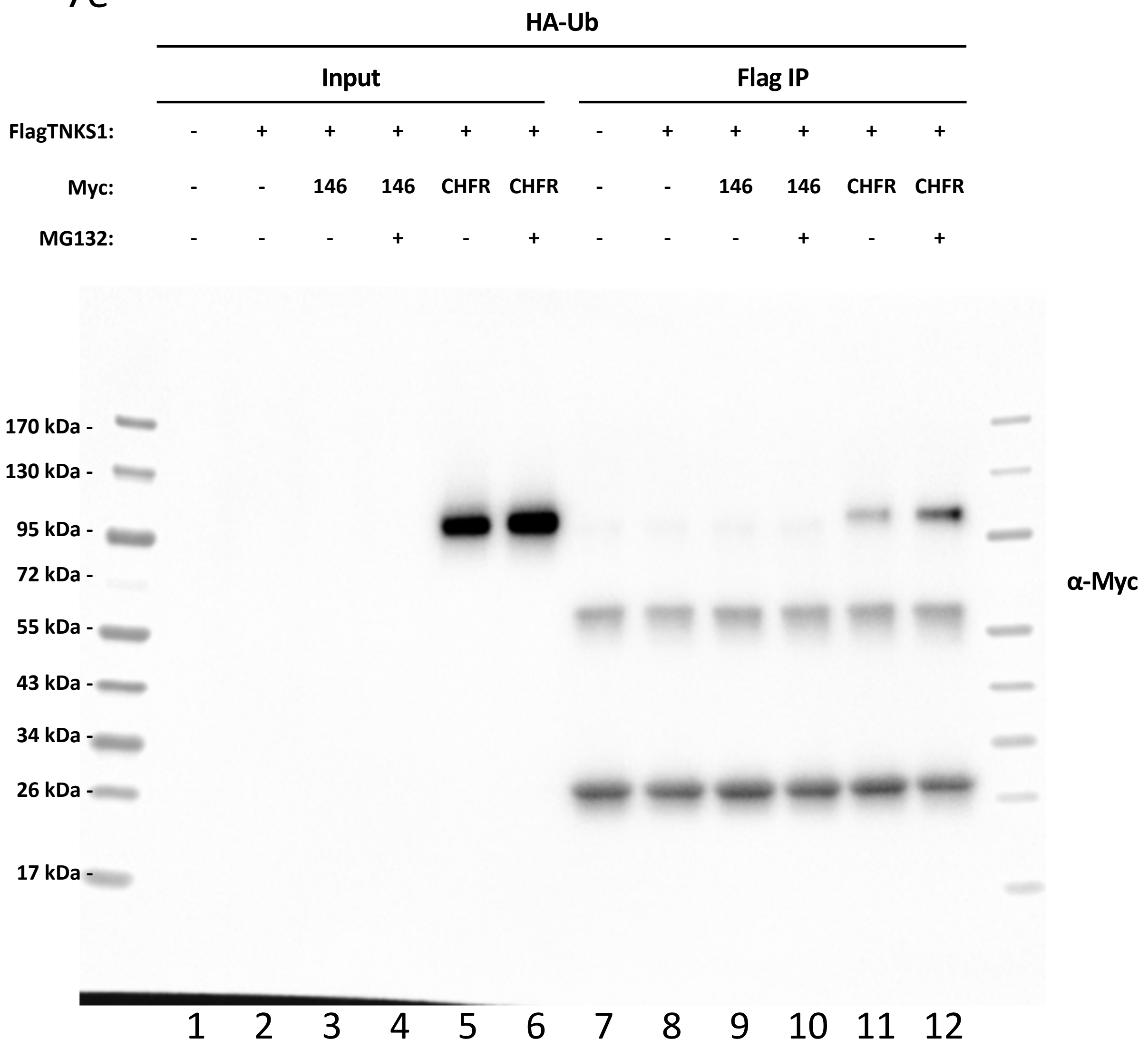
HA-Ub
Input
Flag IP
FlagTNKS1: - + + + + + - + + + + +
Myc: - - 146 146 CHFR CHFR - - 146 146 CHFR CHFR
MG132: - - - + - + - - - + - +
170 kDa -
130 kDa -
95 kDa -
72 kDa -
55 kDa -
43 kDa -
34 kDa -
26 kDa -
17 kDa -
α-Myc
1 2 3 4 5 6 7 8 9 10 11 12

7f

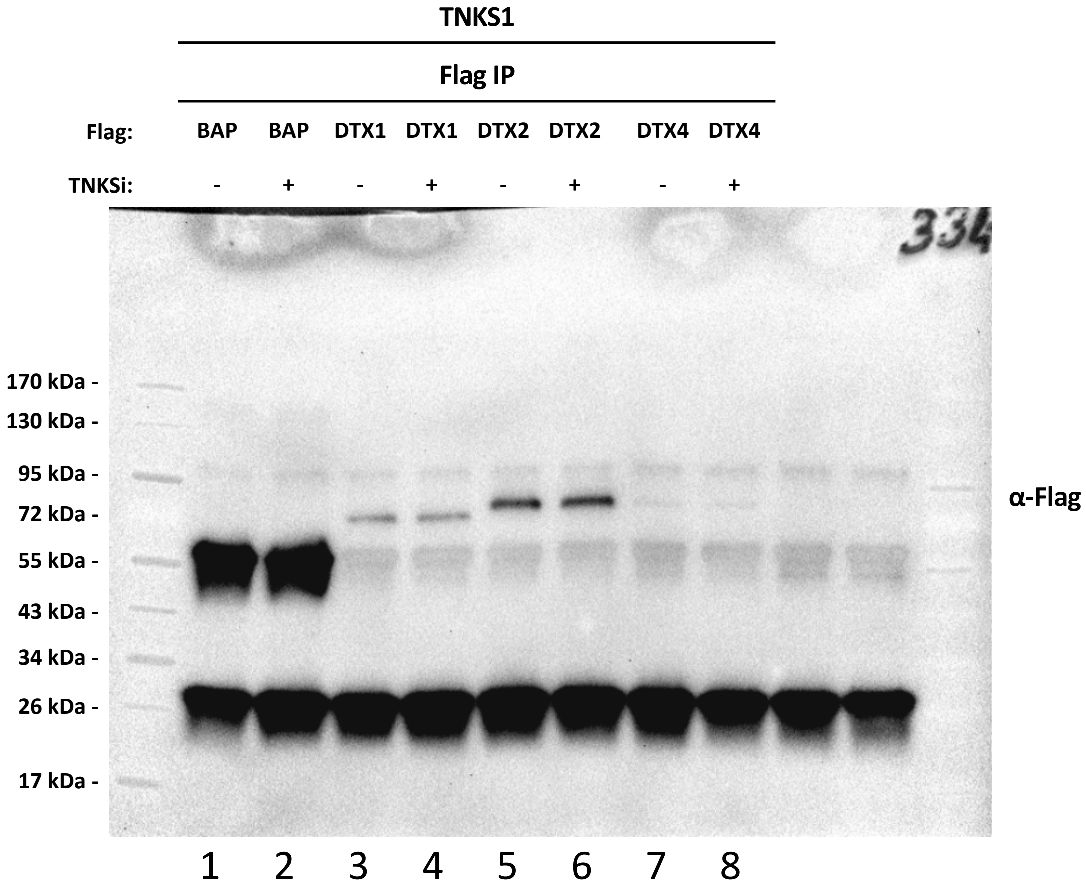
TNKS1
Flag IP
Flag: BAP BAP DTX1 DTX1 DTX2 DTX2 DTX4 DTX4
TNKSi: - + - + - + - +
170 kDa -
130 kDa -
95 kDa -
72 kDa -
55 kDa -
43 kDa -
34 kDa -
26 kDa -
17 kDa -
α-Flag
334
1 2 3 4 5 6 7 8

7f

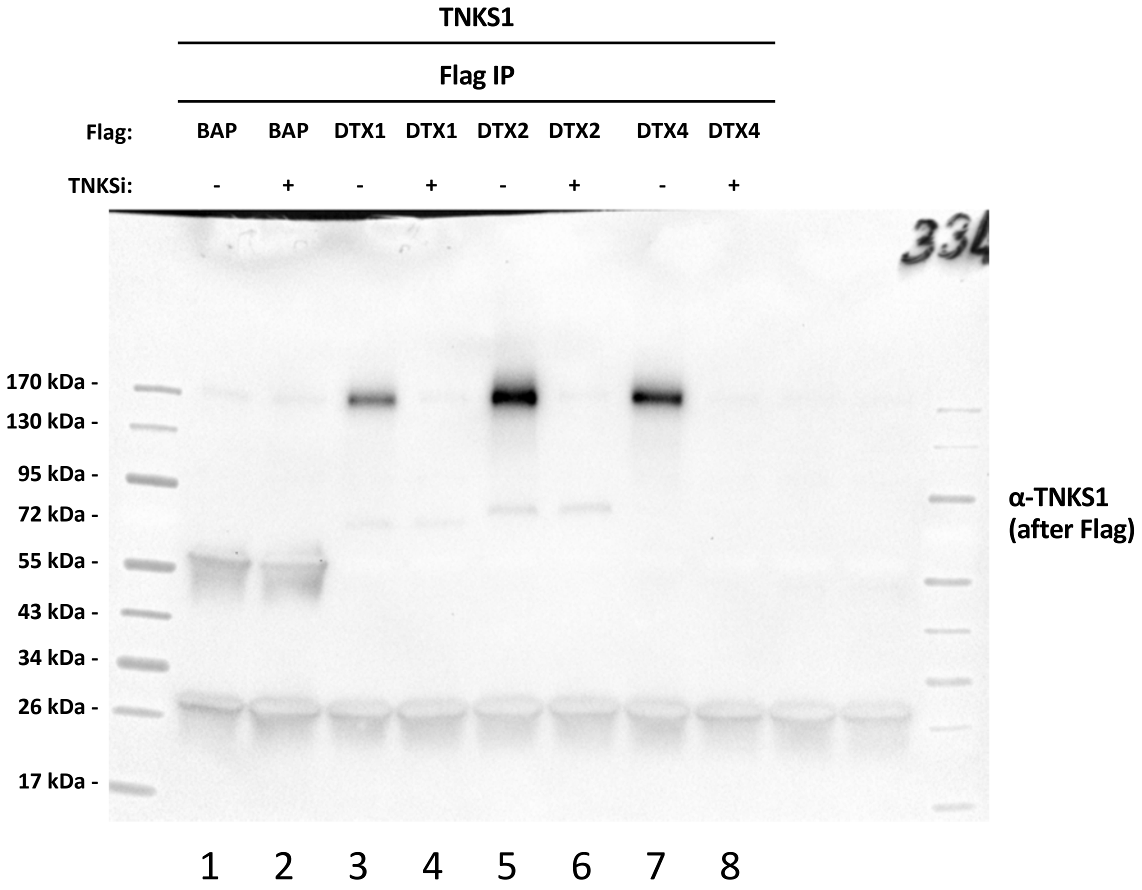
TNKS1
Flag IP
Flag: BAP BAP DTX1 DTX1 DTX2 DTX2 DTX4 DTX4
TNKSi: - + - + - + - +
170 kDa -
130 kDa -
95 kDa -
72 kDa -
55 kDa -
43 kDa -
34 kDa -
26 kDa -
17 kDa -
α-TNKS1
(after Flag)
1 2 3 4 5 6 7 8

7f

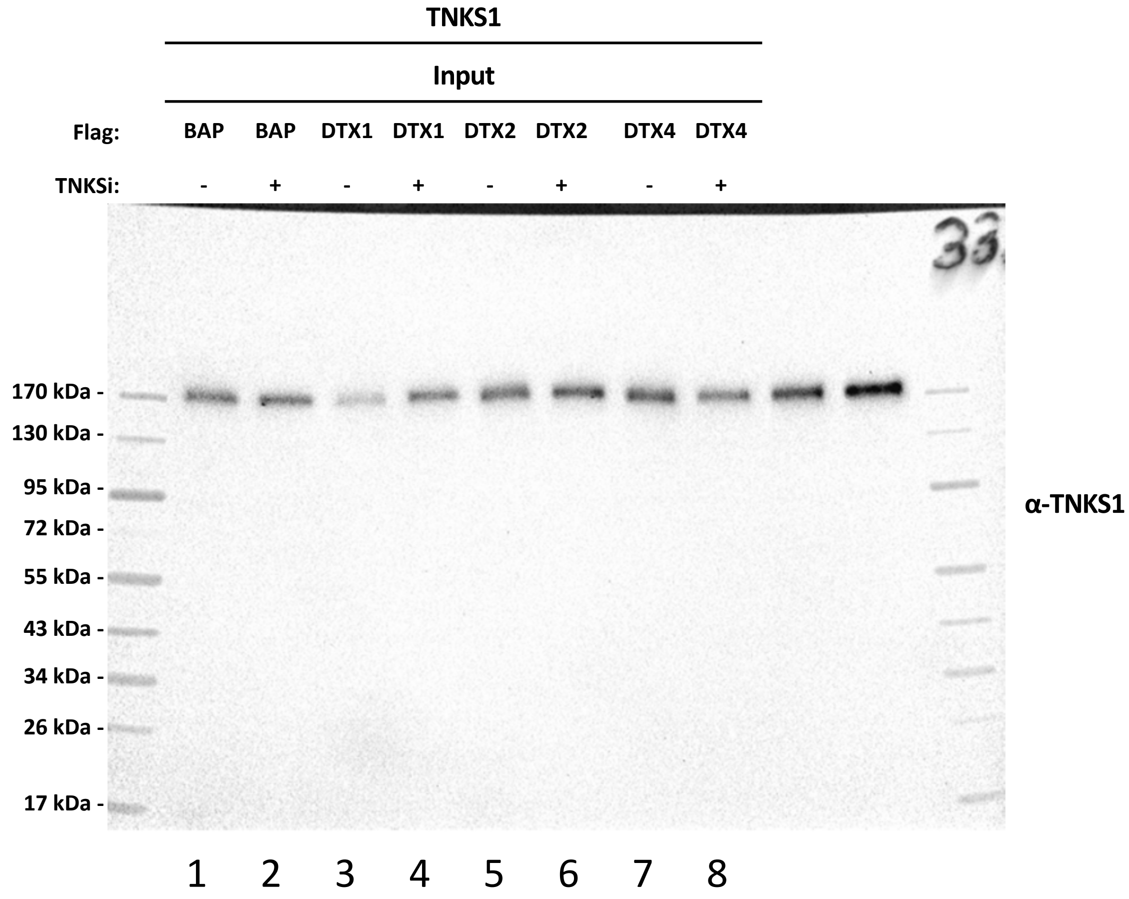

TNKS1
Input
Flag: BAP BAP DTX1 DTX1 DTX2 DTX2 DTX4 DTX4
TNKSi: - + - + - + - +
170 kDa -
130 kDa -
95 kDa -
72 kDa -
55 kDa -
43 kDa -
34 kDa -
26 kDa -
17 kDa -
α-TNKS1
1 2 3 4 5 6 7 8

7f

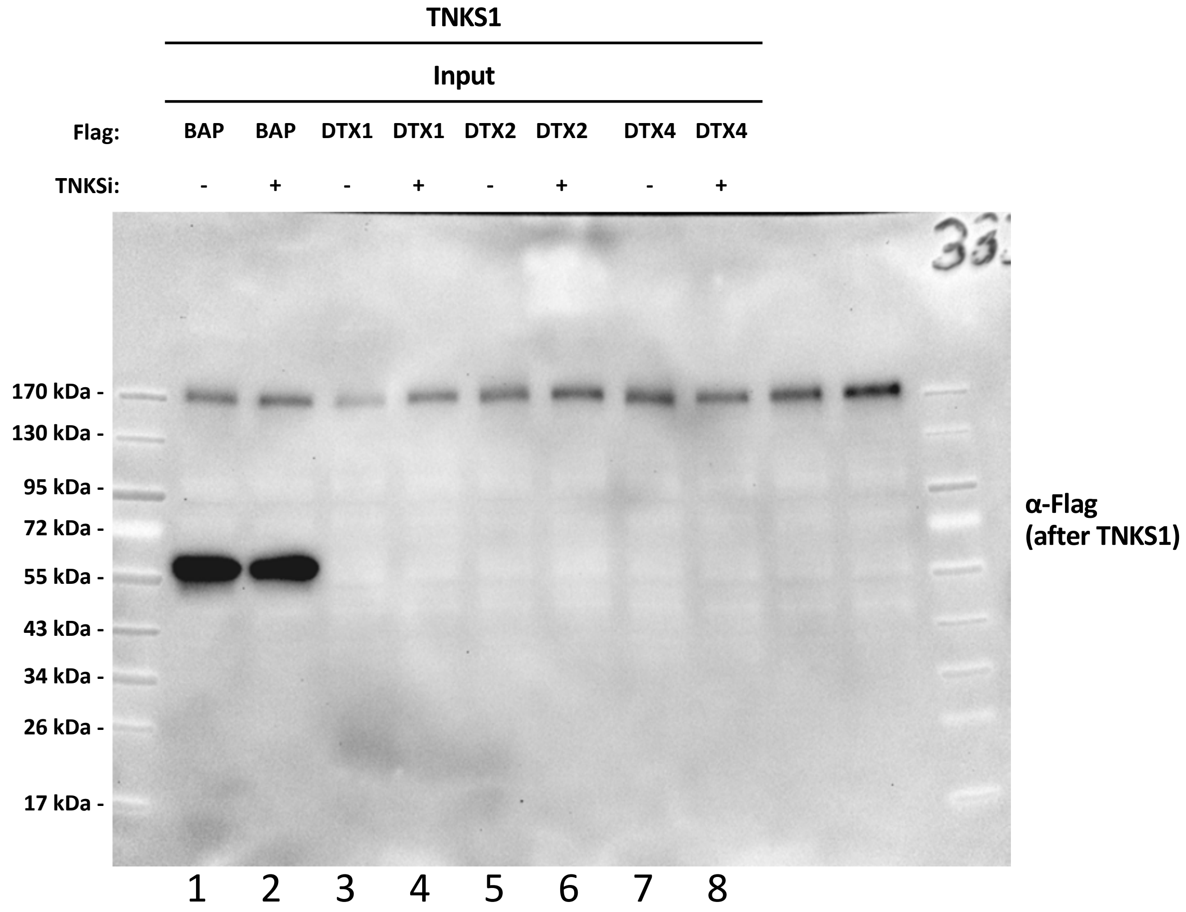
TNKS1
Input
Flag: BAP BAP DTX1 DTX1 DTX2 DTX2 DTX4 DTX4
TNKSi: - + - + - + - +
170 kDa -
130 kDa -
95 kDa -
72 kDa -
55 kDa -
43 kDa -
34 kDa -
26 kDa -
17 kDa -
α-Flag
(after TNKS1)
1 2 3 4 5 6 7 8

7g

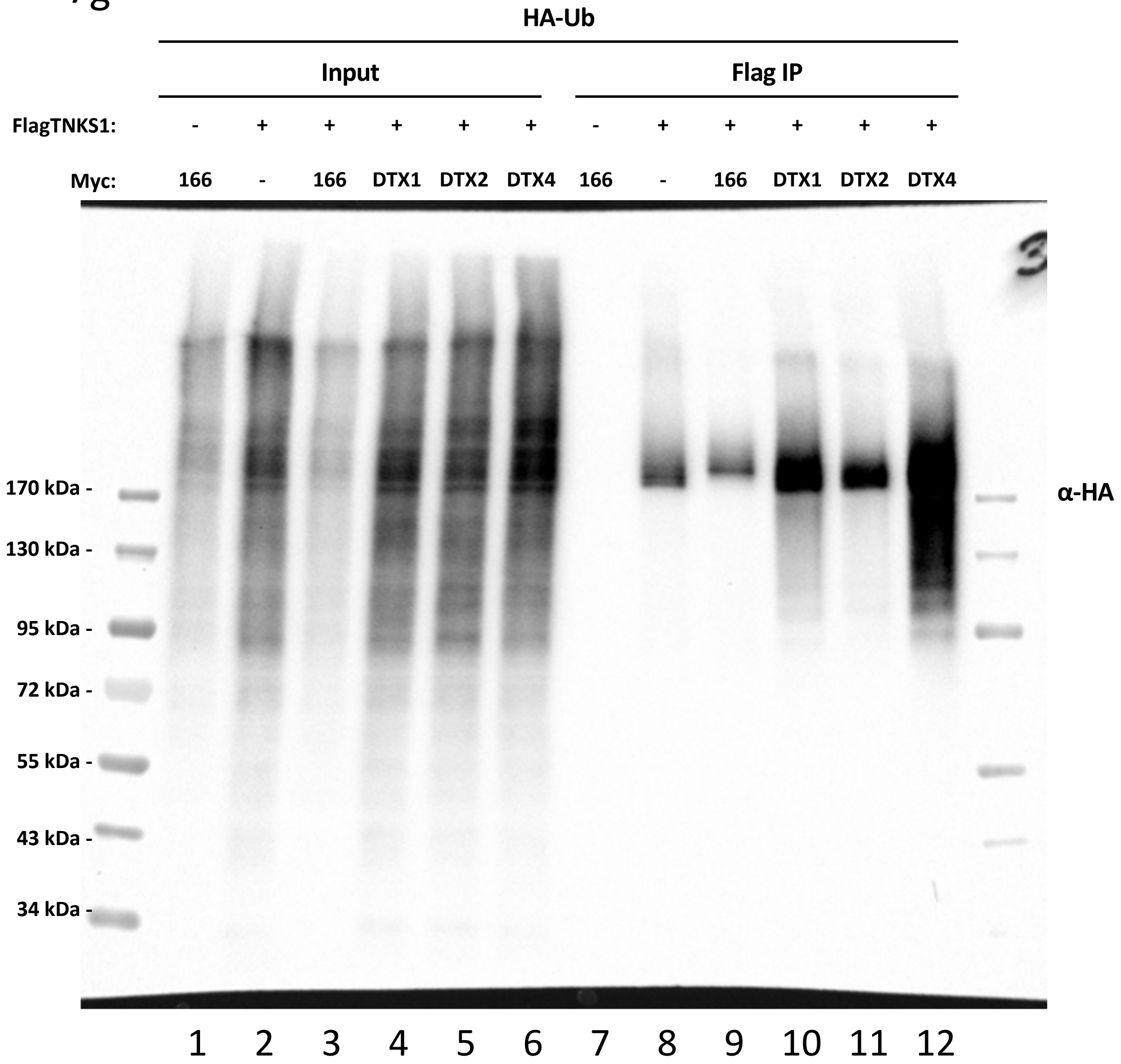
HA-Ub
Input
Flag IP
FlagTNKS1: - + + + + + - + + + + +
Myc: 166 - 166 DTX1 DTX2 DTX4 166 - 166 DTX1 DTX2 DTX4
170 kDa -
130 kDa -
95 kDa -
72 kDa -
55 kDa -
43 kDa -
34 kDa -
α-HA
1 2 3 4 5 6 7 8 9 10 11 12

7g

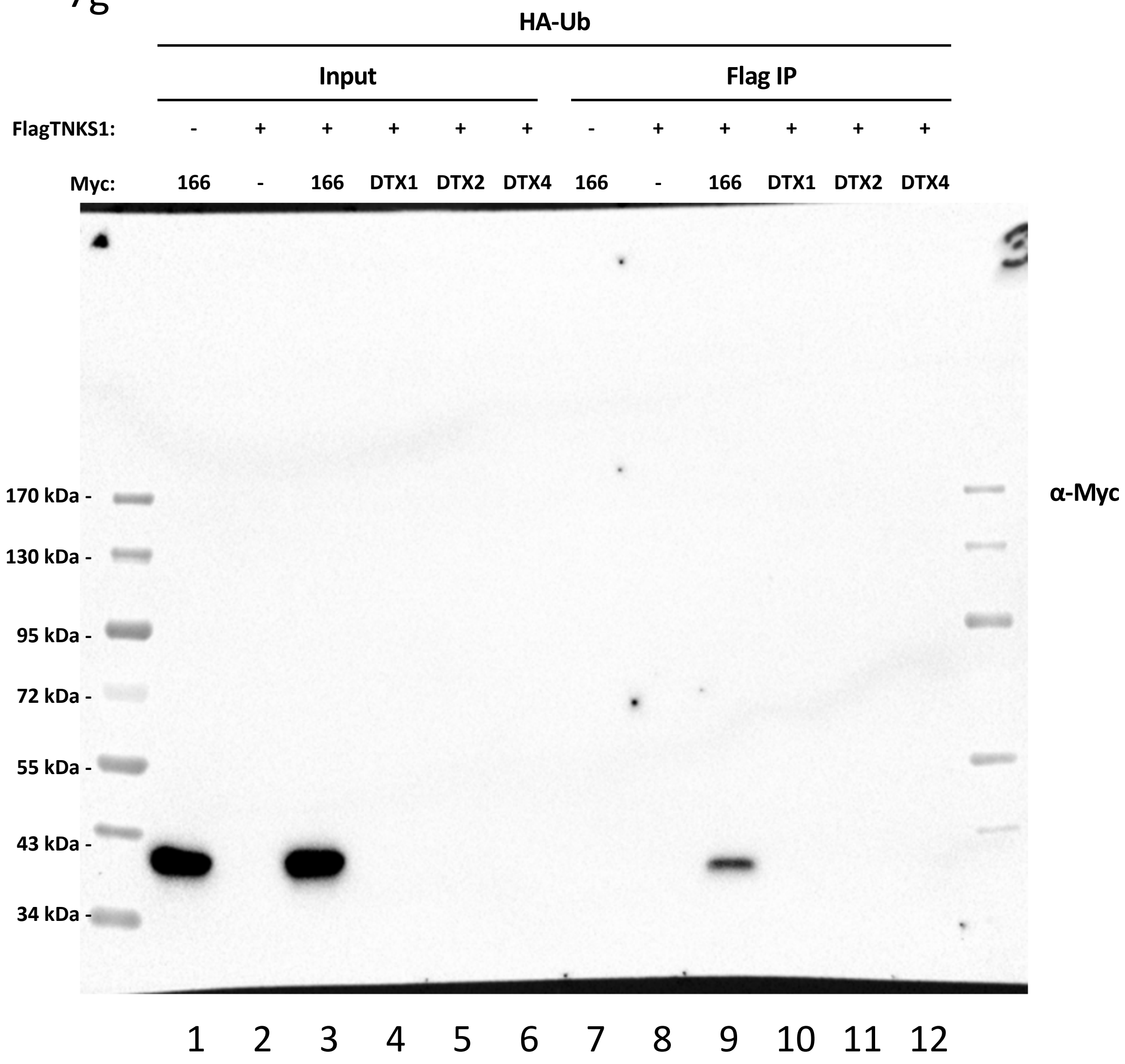
HA-Ub
Input
Flag IP
FlagTNKS1: - + + + + + - + + + + +
Myc: 166 - 166 DTX1 DTX2 DTX4 166 - 166 DTX1 DTX2 DTX4
170 kDa -
130 kDa -
95 kDa -
72 kDa -
55 kDa -
43 kDa -
34 kDa -
α-Myc
1 2 3 4 5 6 7 8 9 10 11 12

7g

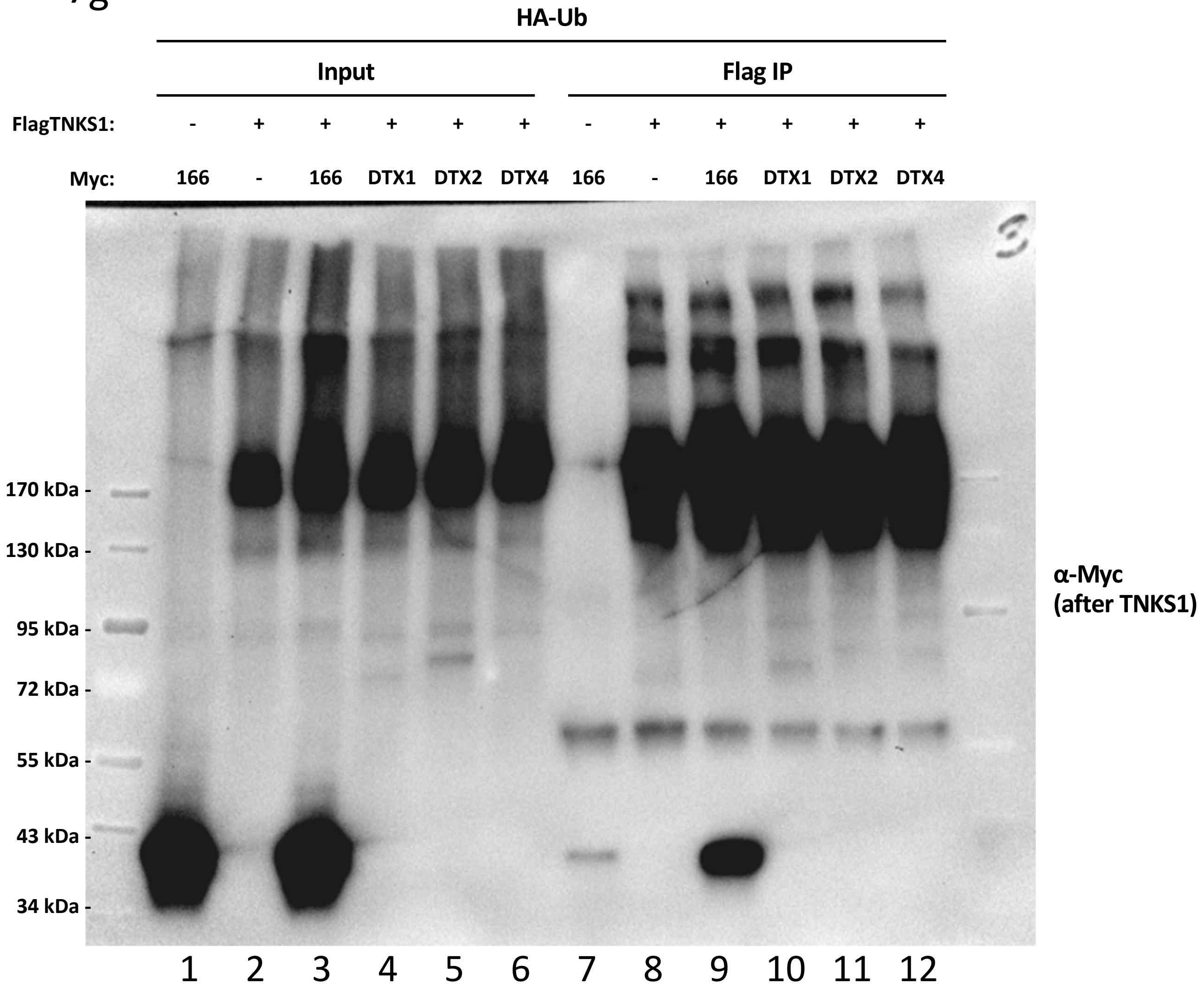
HA-Ub
Input
Flag IP
FlagTNKS1: - + + + + + - + + + + +
Myc: 166 - 166 DTX1 DTX2 DTX4 166 - 166 DTX1 DTX2 DTX4
170 kDa -
130 kDa -
95 kDa -
72 kDa -
55 kDa -
43 kDa -
34 kDa -
α-Myc
(after TNKS1)
1 2 3 4 5 6 7 8 9 10 11 12

7g

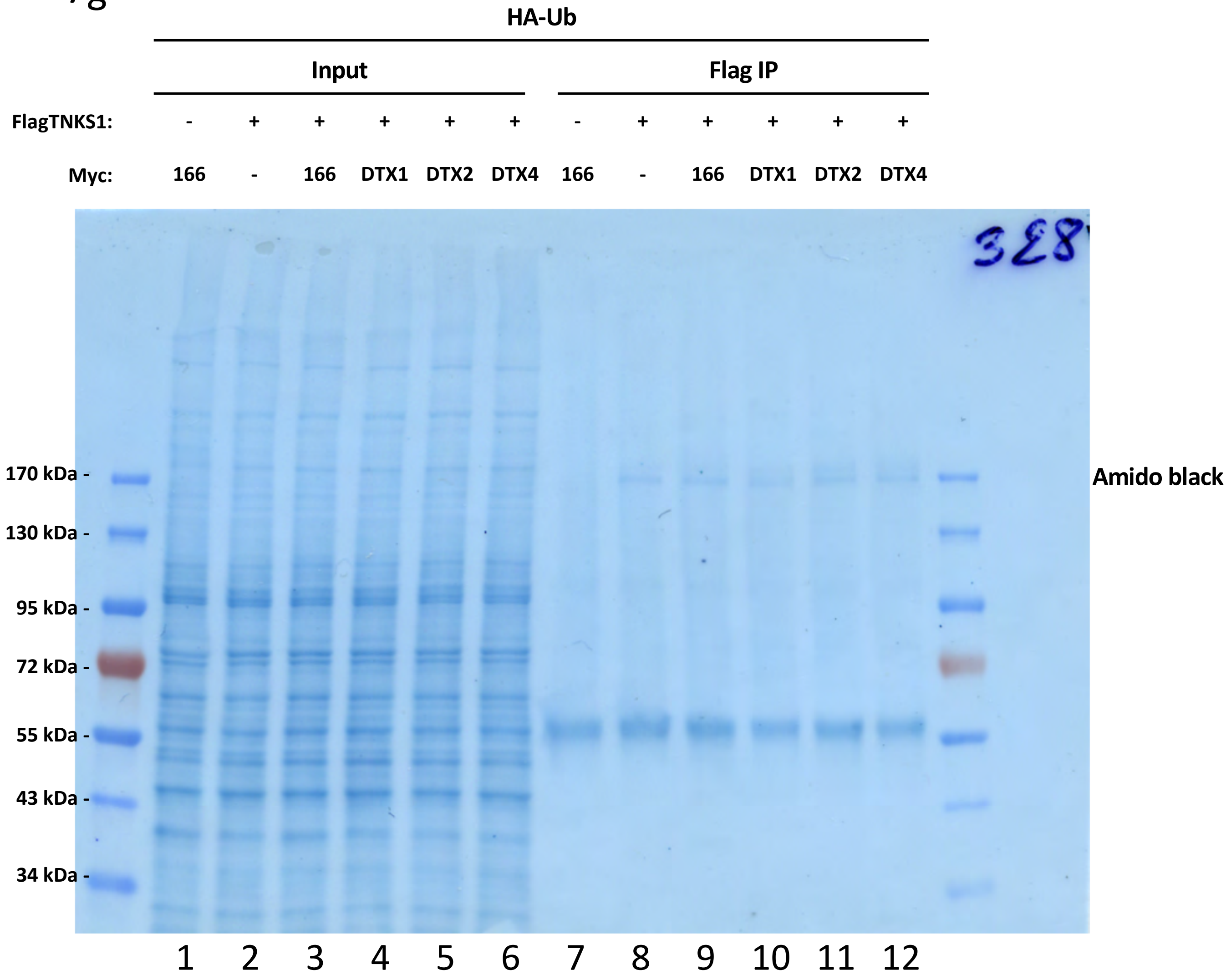
HA-Ub
Input
Flag IP
FlagTNKS1:
- + + + + + - + + + + +
Myc:
166 - 166 DTX1 DTX2 DTX4 166 - 166 DTX1 DTX2 DTX4
328
170 kDa -
130 kDa -
95 kDa -
72 kDa -
55 kDa -
43 kDa -
34 kDa -
Amido black
1 2 3 4 5 6 7 8 9 10 11 12

7h

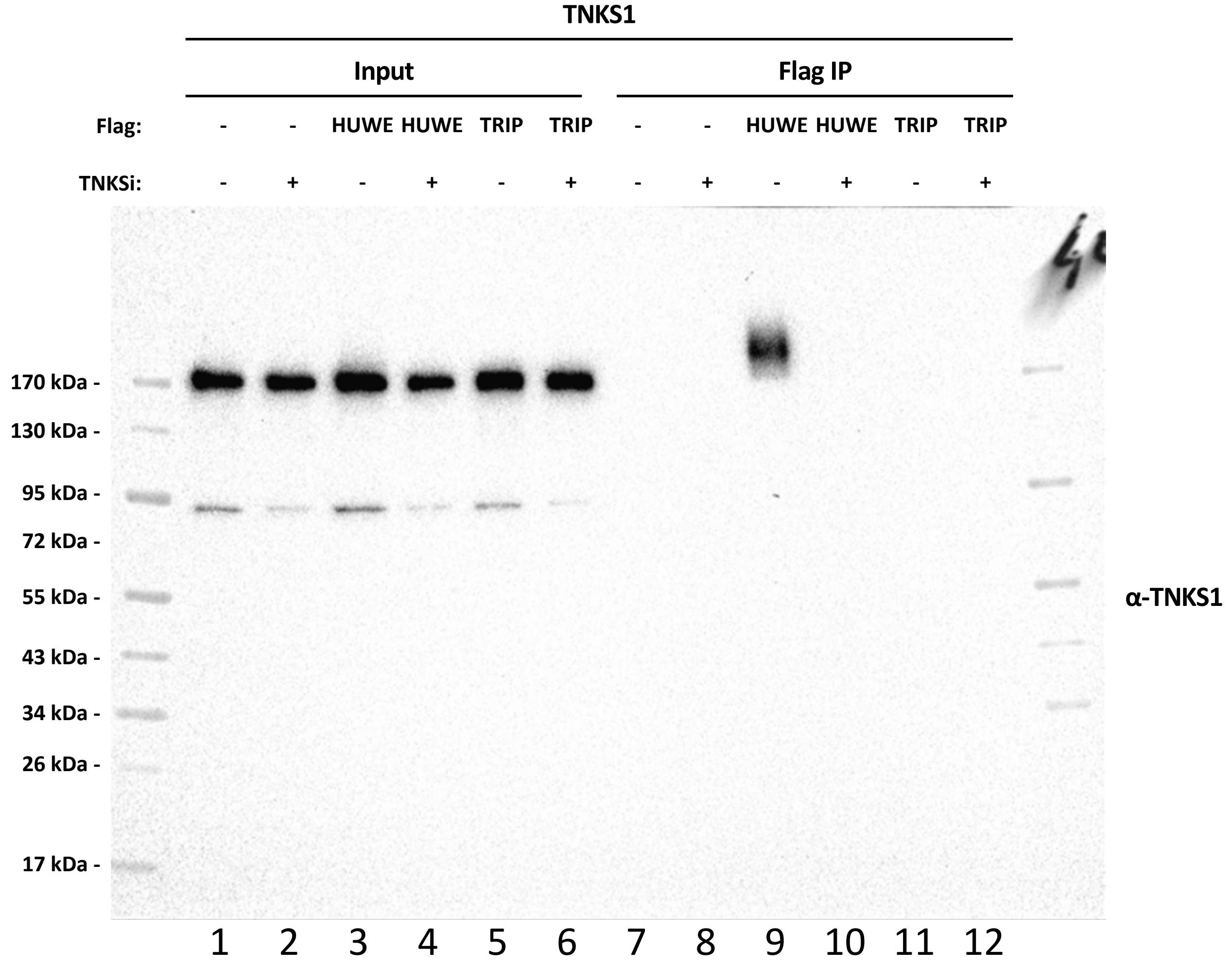
TNKS1
Input
Flag IP
Flag: - - HUWE HUWE TRIP TRIP - - HUWE HUWE TRIP TRIP
TNKSi: - + - + - + - + - + - +
170 kDa -
130 kDa -
95 kDa -
72 kDa -
55 kDa -
43 kDa -
34 kDa -
26 kDa -
17 kDa -
α-TNKS1
1 2 3 4 5 6 7 8 9 10 11 12

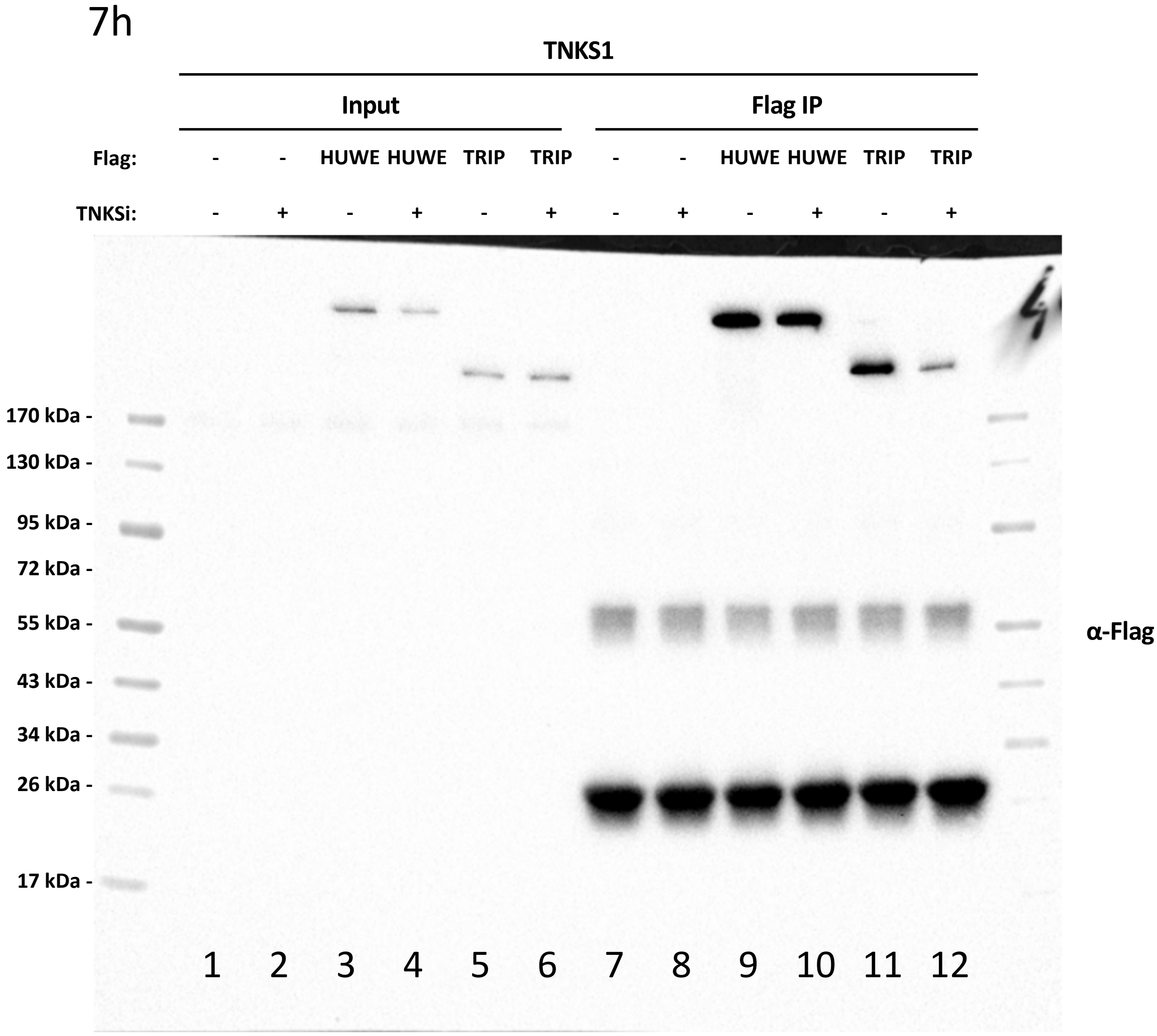
7h
TNKS1
Input
Flag IP
Flag:
-
-
HUWE
HUWE
TRIP
TRIP
-
-
HUWE
HUWE
TRIP
TRIP
TNKSi:
- + - + - + - + - + - +
170 kDa -
130 kDa -
95 kDa -
72 kDa -
55 kDa -
43 kDa -
34 kDa -
26 kDa -
17 kDa -
α-Flag
1 2 3 4 5 6 7 8 9 10 11 12

7i

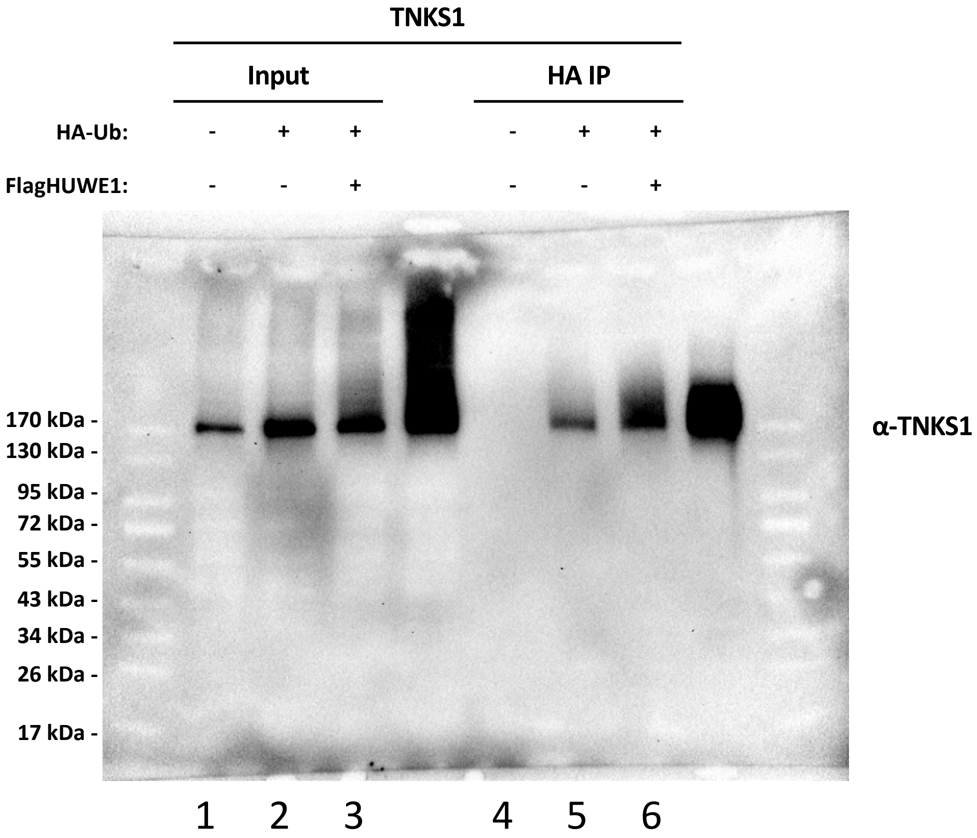
TNKS1
Input
HA IP
HA-Ub: - + + - + +
FlagHUWE1: - - + - - +
170 kDa -
130 kDa -
95 kDa -
72 kDa -
55 kDa -
43 kDa -
34 kDa -
26 kDa -
17 kDa -
α-TNKS1
1 2 3 4 5 6

7i

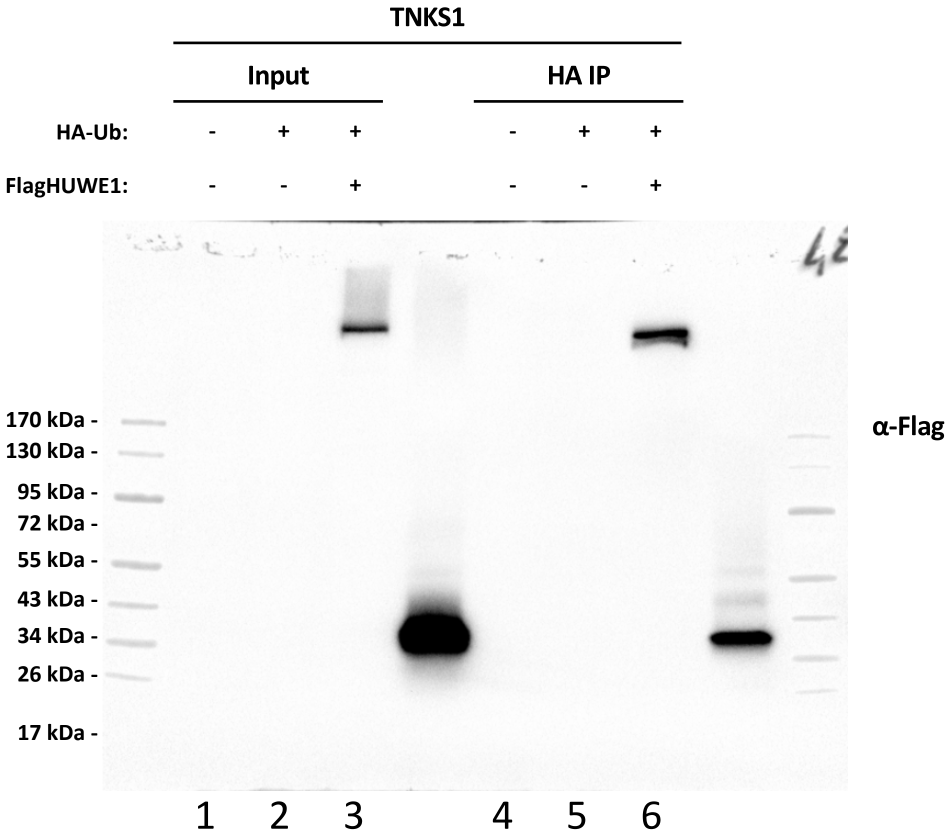
TNKS1
Input
HA IP
HA-Ub:
- + + - + +
FlagHUWE1:
- - + - - +
170 kDa -
130 kDa -
95 kDa -
72 kDa -
55 kDa -
43 kDa -
34 kDa -
26 kDa -
17 kDa -
α-Flag
1 2 3 4 5 6

7i

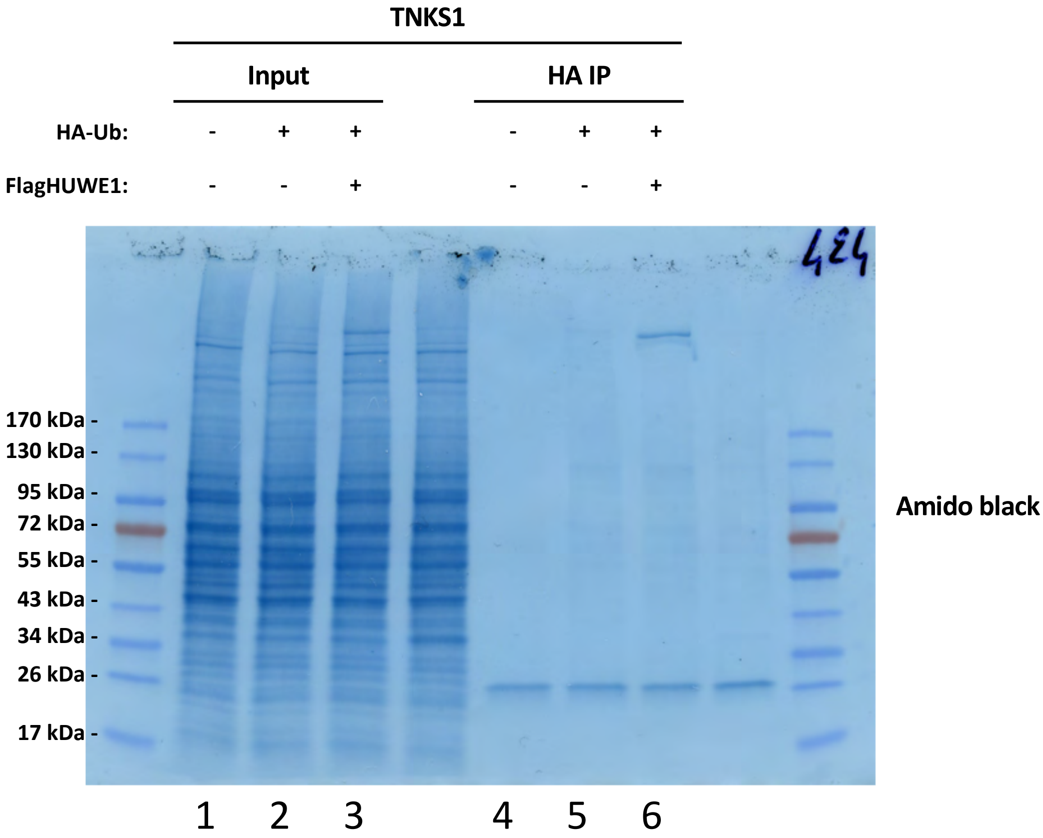

TNKS1
Input
HA IP
HA-Ub: - + + - + +
FlagHUWE1: - - + - - +
170 kDa -
130 kDa -
95 kDa -
72 kDa -
55 kDa -
43 kDa -
34 kDa -
26 kDa -
17 kDa -
424
Amido black
1 2 3 4 5 6

S1a

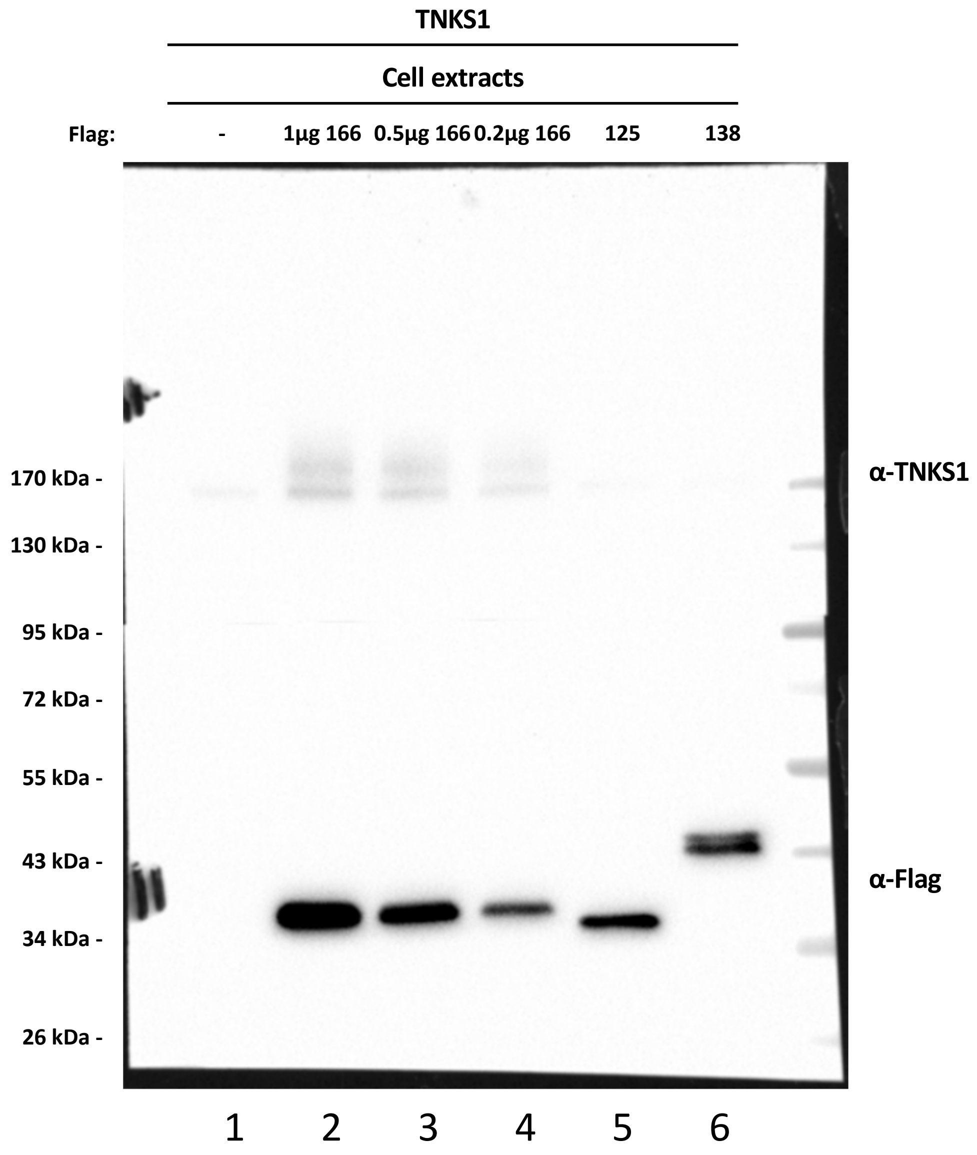
TNKS1
Cell extracts
Flag:
-
1µg 166
0.5µg 166
0.2µg 166
125
138
170 kDa -
130 kDa -
95 kDa -
72 kDa -
55 kDa -
43 kDa -
34 kDa -
26 kDa -
α-TNKS1
α-Flag
1
2
3
4
5
6

S1a

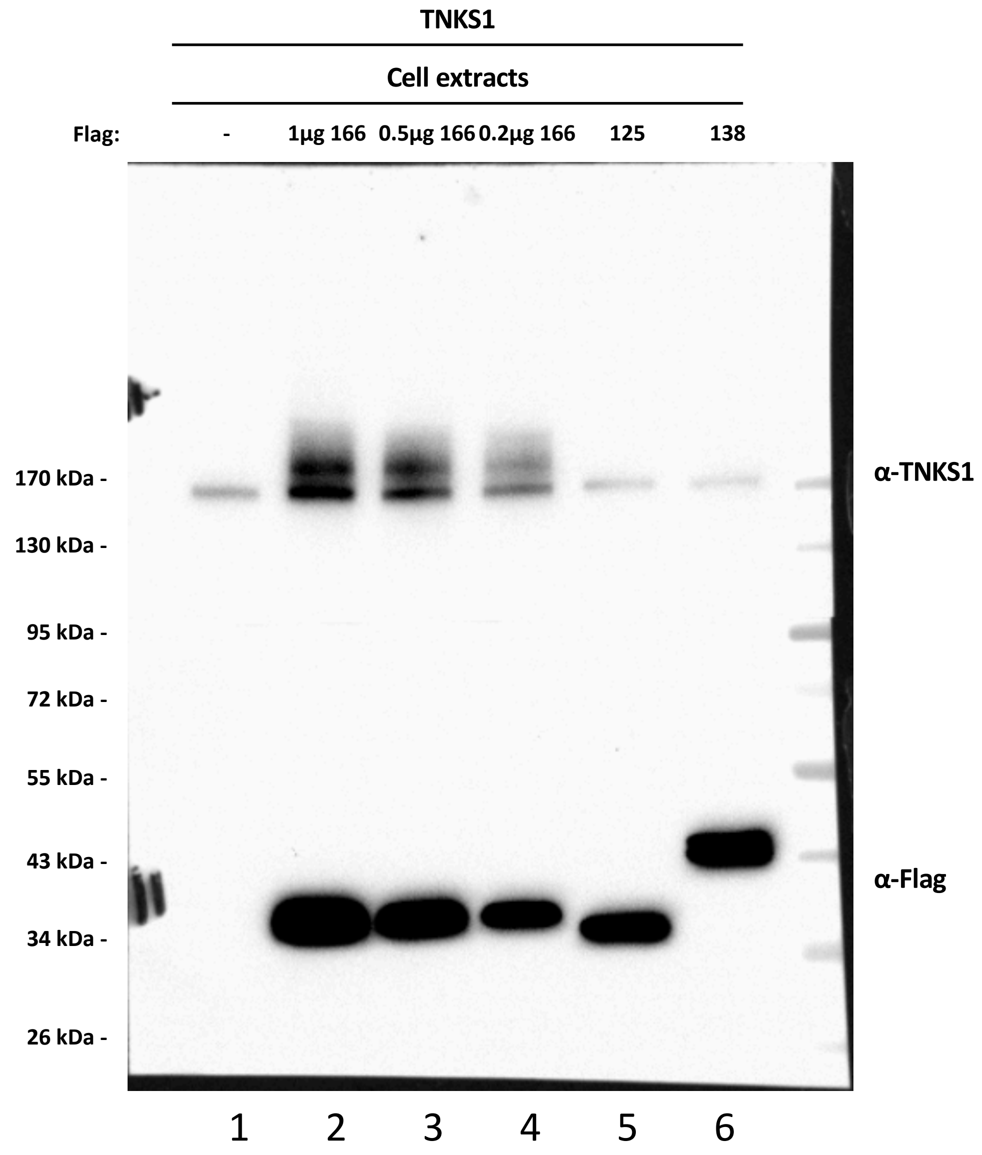

TNKS1
Cell extracts
Flag:
-
1µg 166
0.5µg 166
0.2µg 166
125
138
170 kDa -
130 kDa -
95 kDa -
72 kDa -
55 kDa -
43 kDa -
34 kDa -
26 kDa -
α-TNKS1
α-Flag
1
2
3
4
5
6

S1a

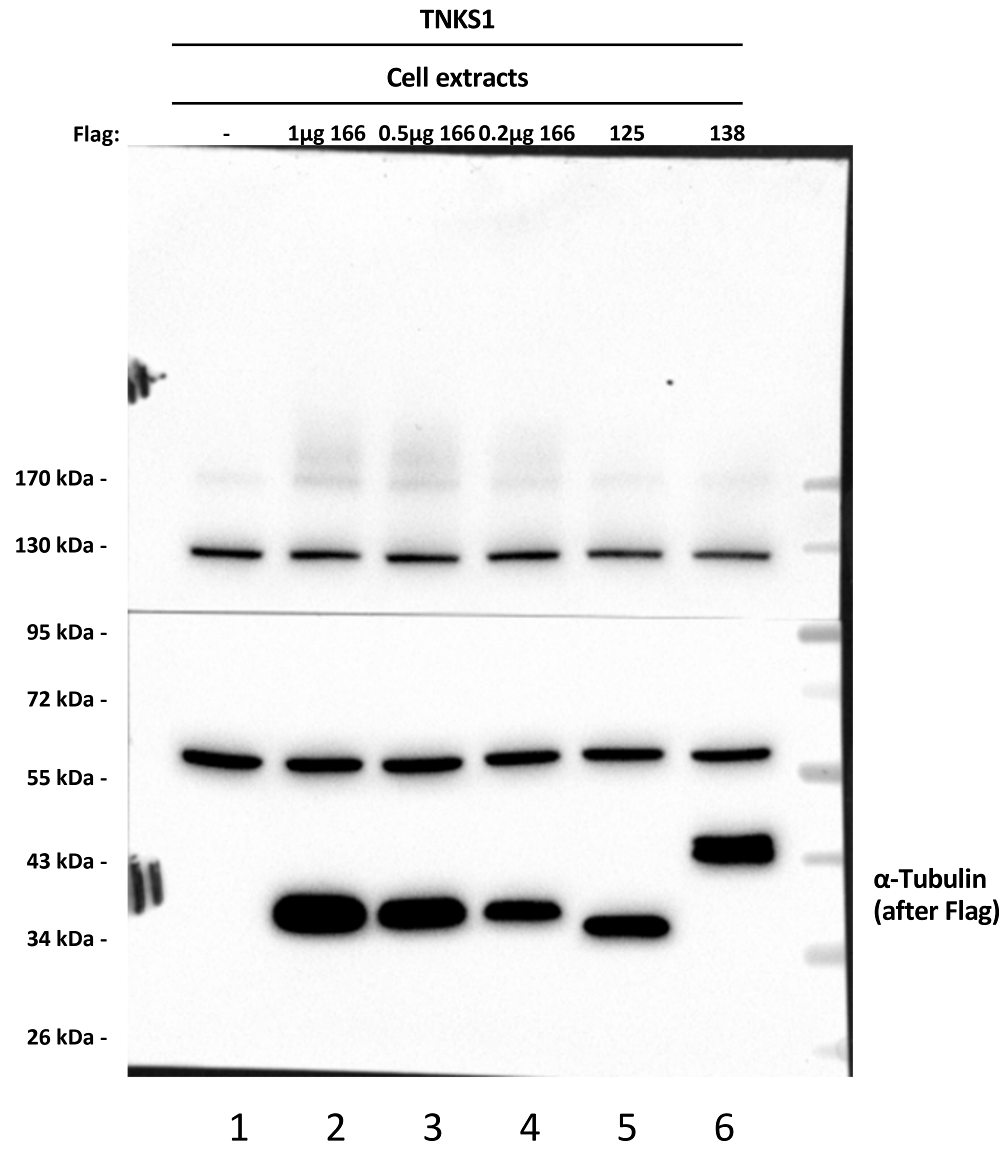
TNKS1
Cell extracts
Flag:
-
1µg 166
0.5µg 166
0.2µg 166
125
138
170 kDa -
130 kDa -
95 kDa -
72 kDa -
55 kDa -
43 kDa -
34 kDa -
26 kDa -
α-Tubulin
(after Flag)
1
2
3
4
5
6

S1b

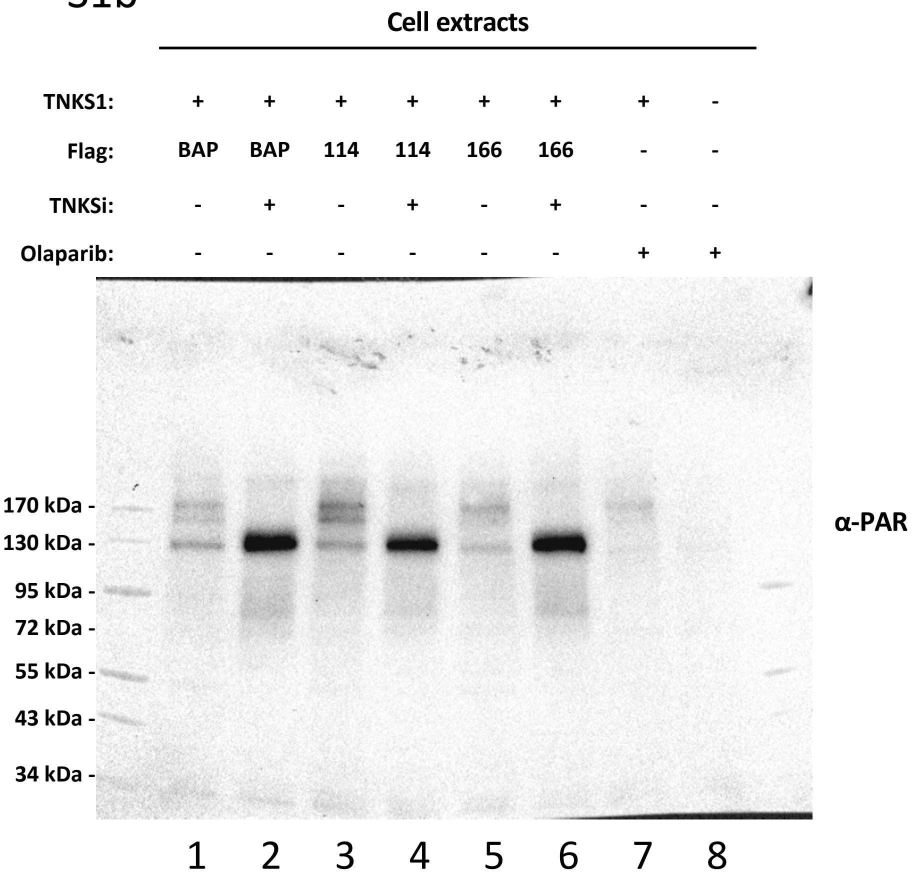
Cell extracts
TNKS1: + + + + + + + -
Flag: BAP BAP 114 114 166 166 - -
TNKSi: - + - + - + - -
Olaparib: - - - - - - + +
170 kDa -
130 kDa -
95 kDa -
72 kDa -
55 kDa -
43 kDa -
34 kDa -
α-PAR
1 2 3 4 5 6 7 8

S1b

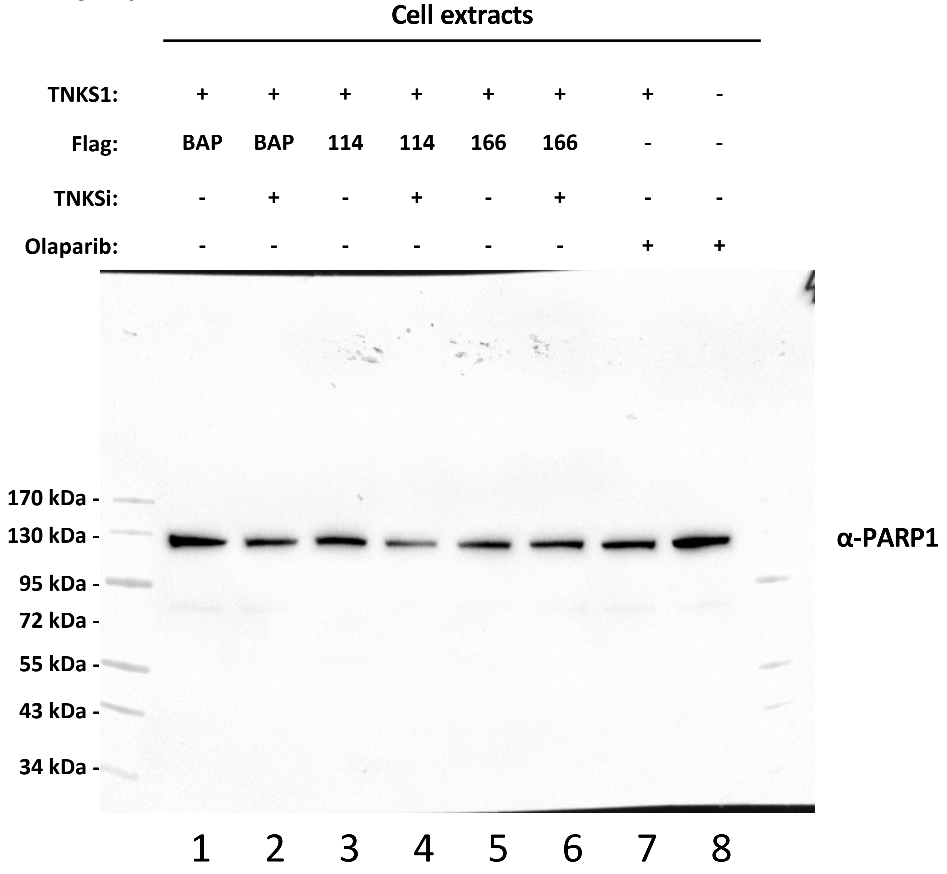
Cell extracts
TNKS1: + + + + + + + -
Flag: BAP BAP 114 114 166 166 - -
TNKSi: - + - + - + - -
Olaparib: - - - - - - + +
170 kDa -
130 kDa -
95 kDa -
72 kDa -
55 kDa -
43 kDa -
34 kDa -
α-PARP1
1 2 3 4 5 6 7 8

S1b

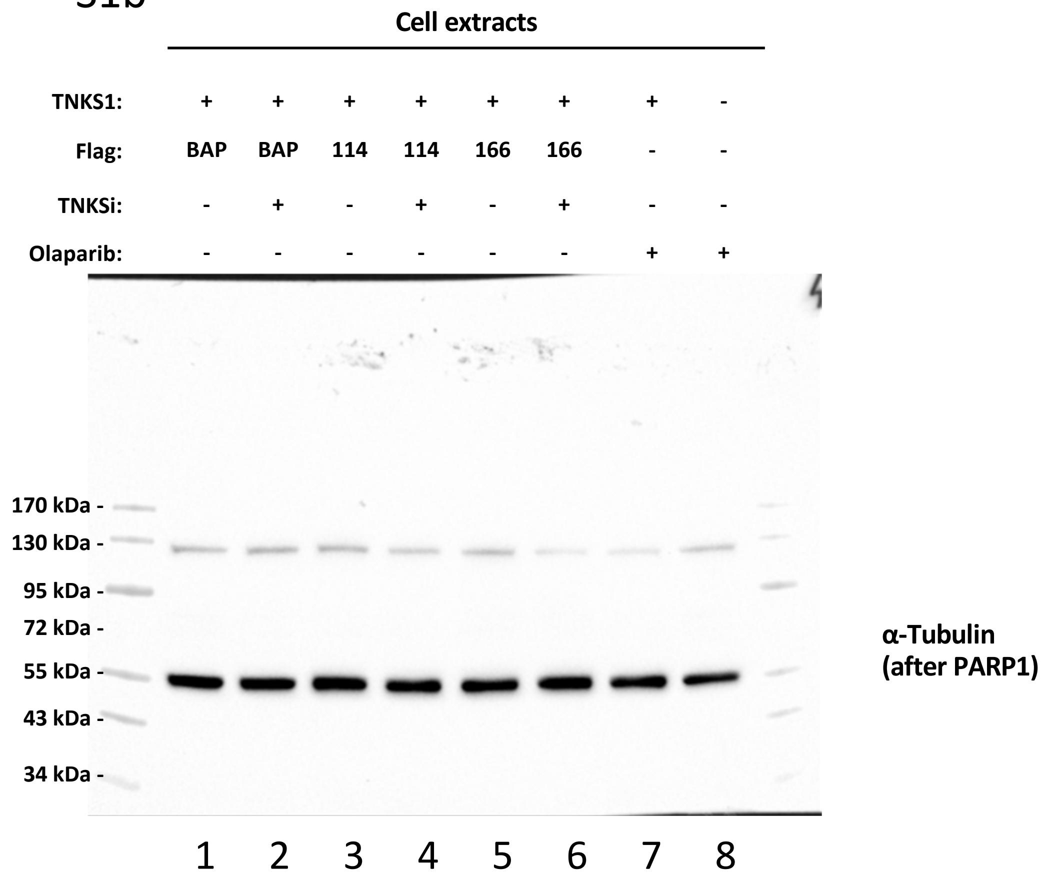
Cell extracts
TNKS1: + + + + + + + -
Flag: BAP BAP 114 114 166 166 - -
TNKSi: - + - + - + - -
Olaparib: - - - - - - + +
170 kDa -
130 kDa -
95 kDa -
72 kDa -
55 kDa -
43 kDa -
34 kDa -
α-Tubulin
(after PARP1)
1 2 3 4 5 6 7 8

S1b

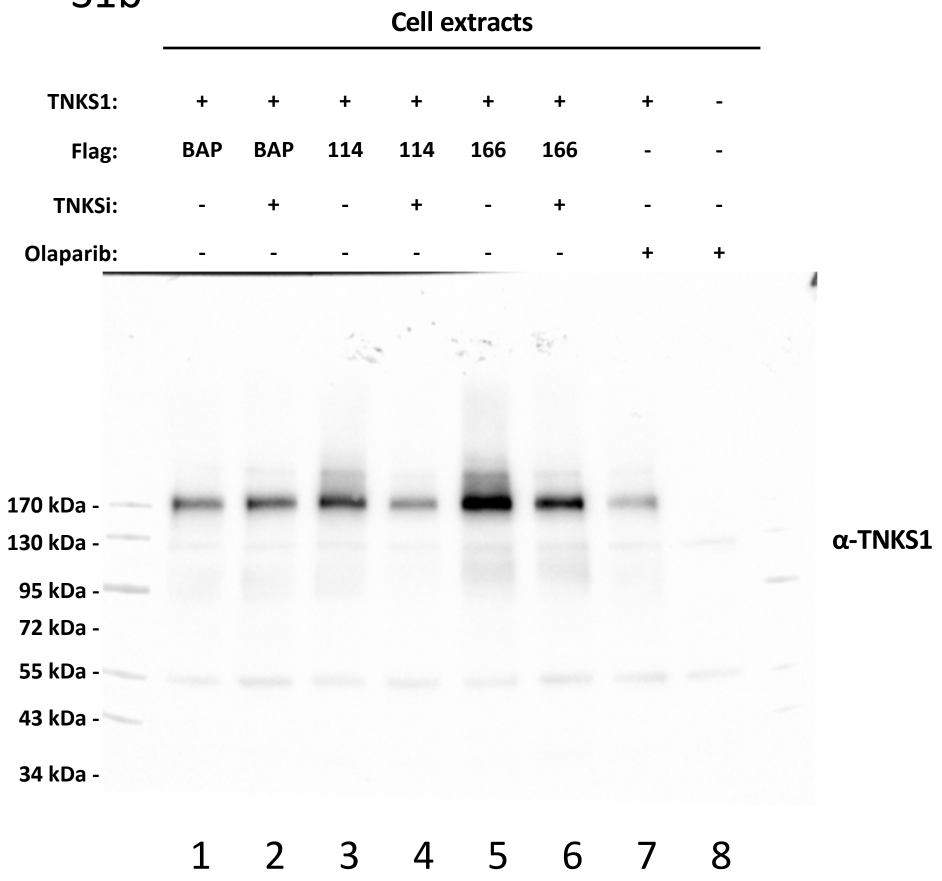
Cell extracts
TNKS1: + + + + + + + -
Flag: BAP BAP 114 114 166 166 - -
TNKSi: - + - + - + - -
Olaparib: - - - - - - + +
170 kDa -
130 kDa -
95 kDa -
72 kDa -
55 kDa -
43 kDa -
34 kDa -
α-TNKS1
1 2 3 4 5 6 7 8

S1b

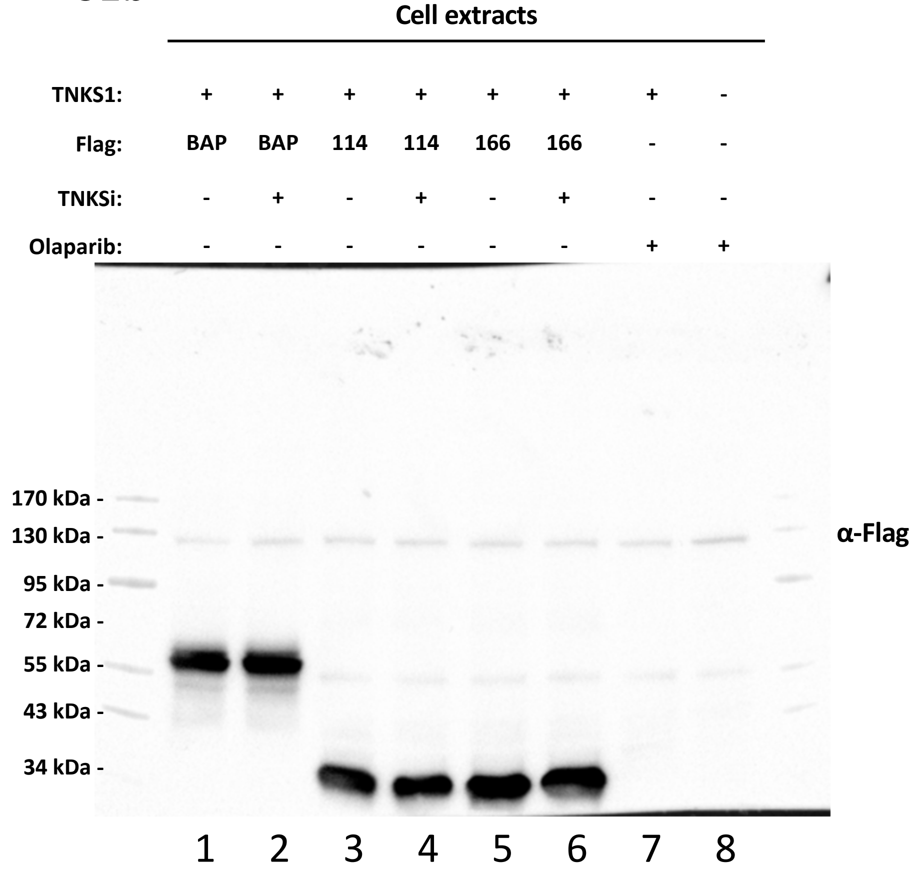

Cell extracts
TNKS1: + + + + + + + -
Flag: BAP BAP 114 114 166 166 - -
TNKSi: - + - + - + - -
Olaparib: - - - - - - + +
170 kDa -
130 kDa -
95 kDa -
72 kDa -
55 kDa -
43 kDa -
34 kDa -
α-Flag
1 2 3 4 5 6 7 8

S1c

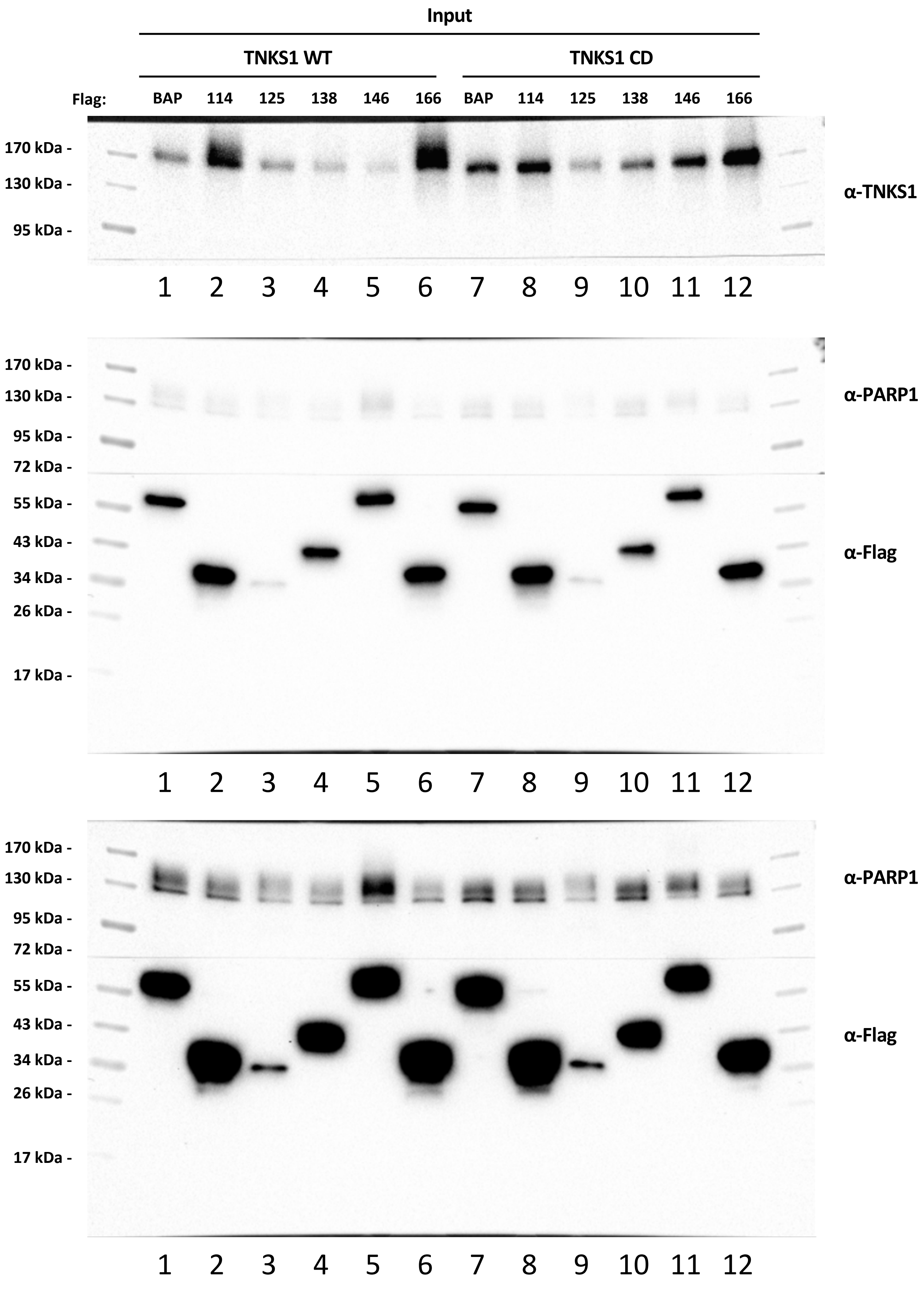
Input
TNKS1 WT
TNKS1 CD
Flag:
BAP
114
125
138
146
166
BAP
114
125
138
146
166
170 kDa -
130 kDa -
95 kDa -
α-TNKS1
1 2 3 4 5 6 7 8 9 10 11 12
170 kDa -
130 kDa -
95 kDa -
72 kDa -
55 kDa -
43 kDa -
34 kDa -
26 kDa -
17 kDa -
α-PARP1
α-Flag
1 2 3 4 5 6 7 8 9 10 11 12
170 kDa -
130 kDa -
95 kDa -
72 kDa -
55 kDa -
43 kDa -
34 kDa -
26 kDa -
17 kDa -
α-PARP1
α-Flag
1 2 3 4 5 6 7 8 9 10 11 12

S1c

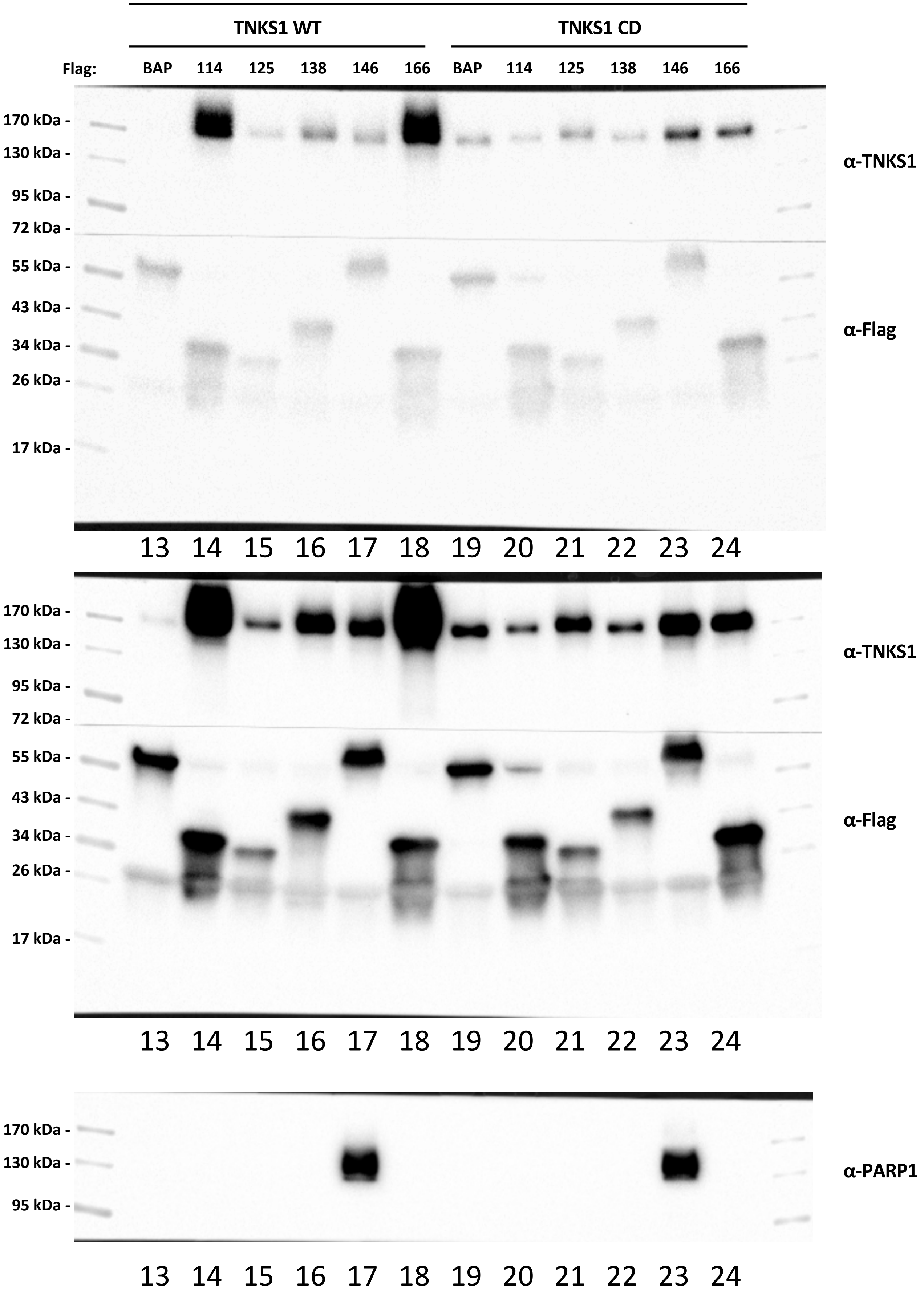
Flag IP
TNKS1 WT
TNKS1 CD
Flag: BAP 114 125 138 146 166 BAP 114 125 138 146 166
170 kDa -
130 kDa -
95 kDa -
72 kDa -
55 kDa -
43 kDa -
34 kDa -
26 kDa -
17 kDa -
α-TNKS1
α-Flag
13 14 15 16 17 18 19 20 21 22 23 24
170 kDa -
130 kDa -
95 kDa -
72 kDa -
55 kDa -
43 kDa -
34 kDa -
26 kDa -
17 kDa -
α-TNKS1
α-Flag
13 14 15 16 17 18 19 20 21 22 23 24
170 kDa -
130 kDa -
95 kDa -
α-PARP1
13 14 15 16 17 18 19 20 21 22 23 24

S1d

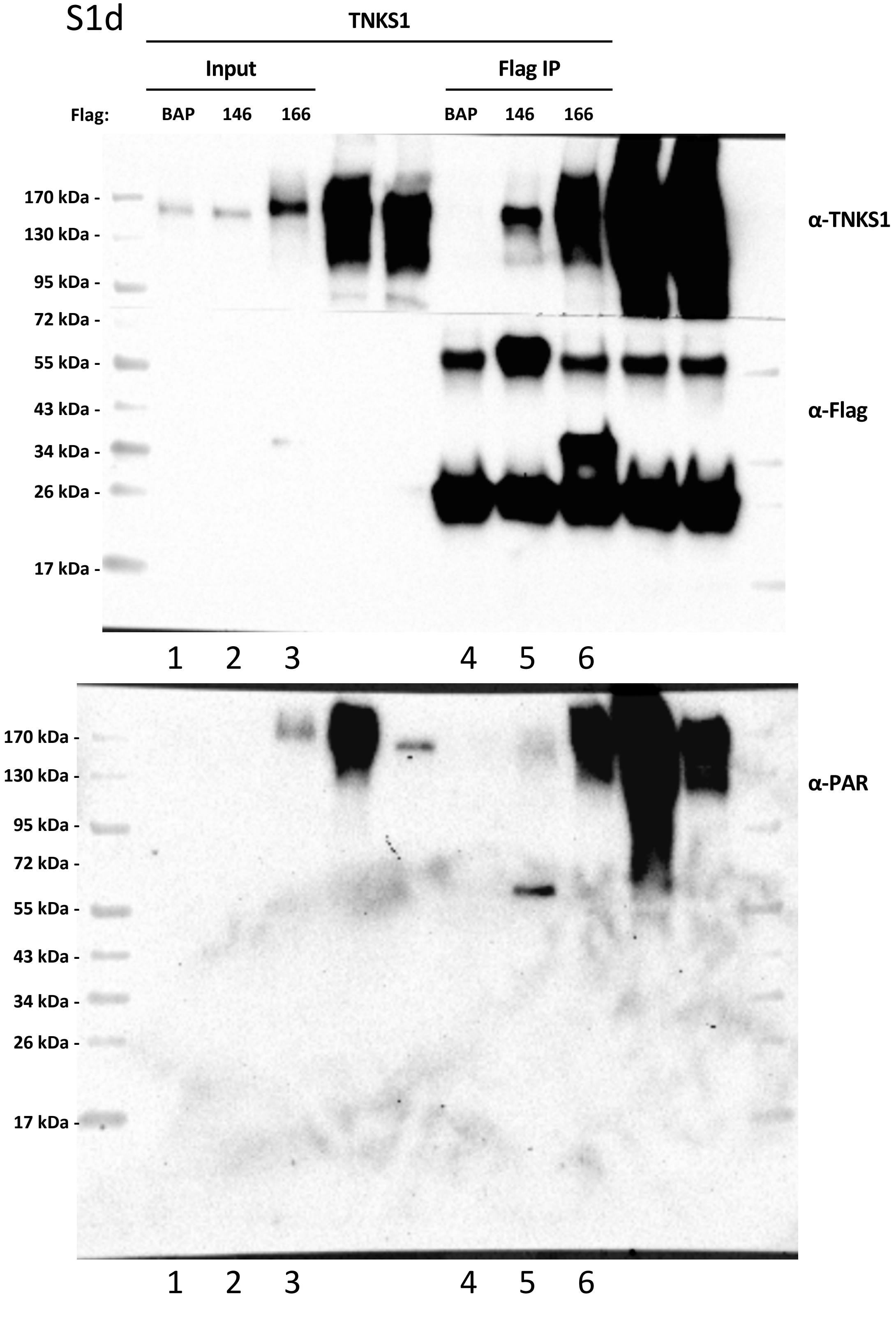
TNKS1
Input
Flag IP
Flag:
BAP
146
166
BAP
146
166
170 kDa -
130 kDa -
95 kDa -
72 kDa -
55 kDa -
43 kDa -
34 kDa -
26 kDa -
17 kDa -
α-TNKS1
α-Flag
1 2 3 4 5 6
170 kDa -
130 kDa -
95 kDa -
72 kDa -
55 kDa -
43 kDa -
34 kDa -
26 kDa -
17 kDa -
α-PAR
1 2 3 4 5 6

S2a

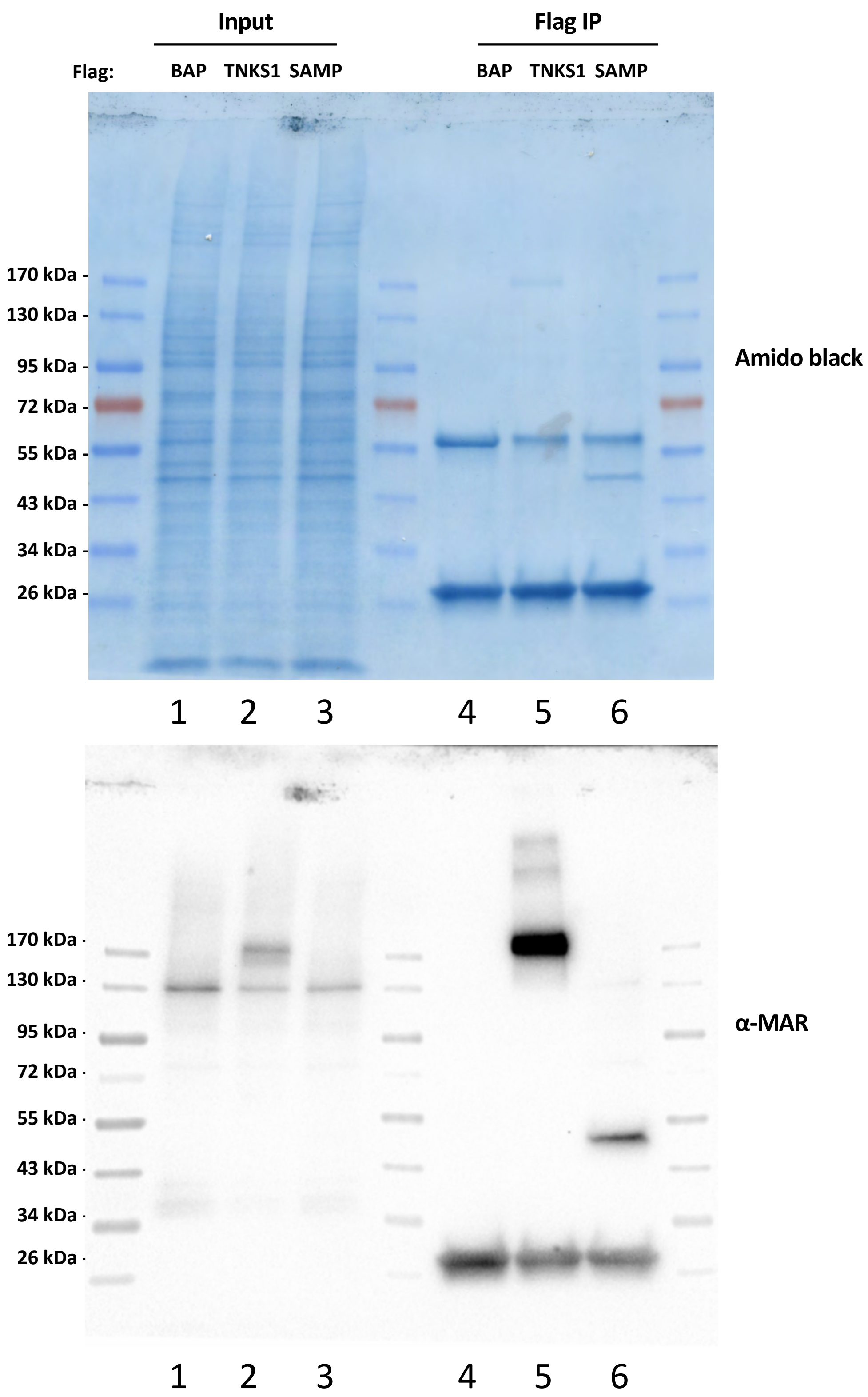

Input
Flag IP
Flag:
BAP
TNKS1
SAMP
BAP
TNKS1
SAMP
170 kDa
130 kDa
95 kDa
72 kDa
55 kDa
43 kDa
34 kDa
26 kDa
Amido black
1
2
3
4
5
6
170 kDa
130 kDa
95 kDa
72 kDa
55 kDa
43 kDa
34 kDa
26 kDa
α-MAR
1
2
3
4
5
6

S2a

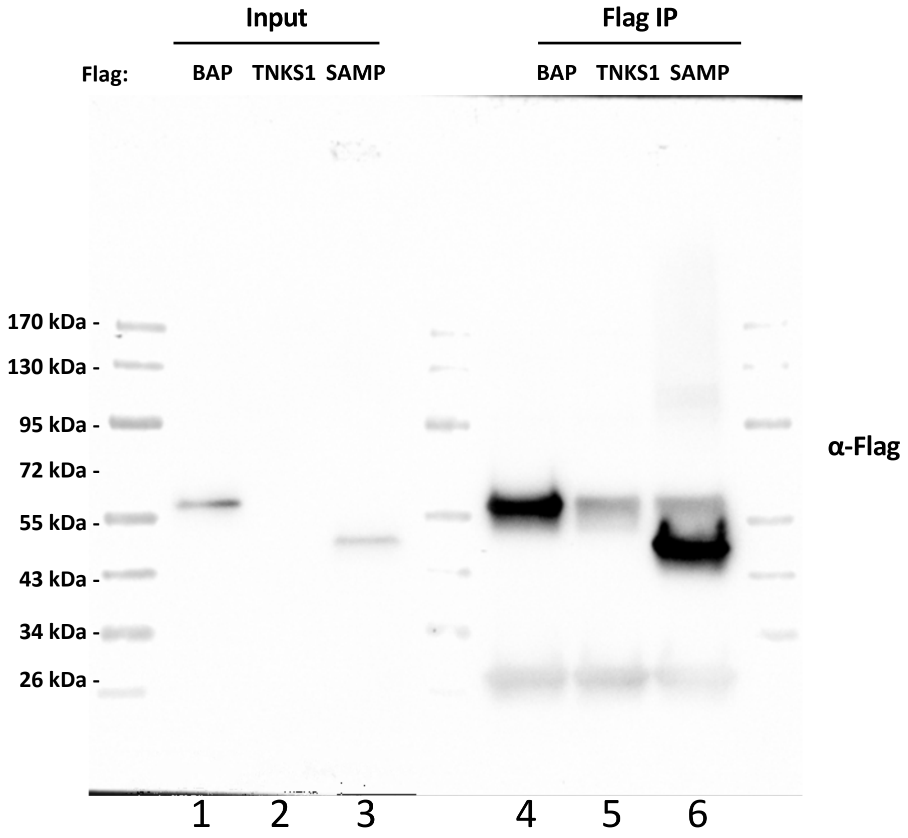
Input
Flag IP
Flag:
BAP
TNKS1
SAMP
BAP
TNKS1
SAMP
170 kDa -
130 kDa -
95 kDa -
72 kDa -
55 kDa -
43 kDa -
34 kDa -
26 kDa -
α-Flag
1
2
3
4
5
6

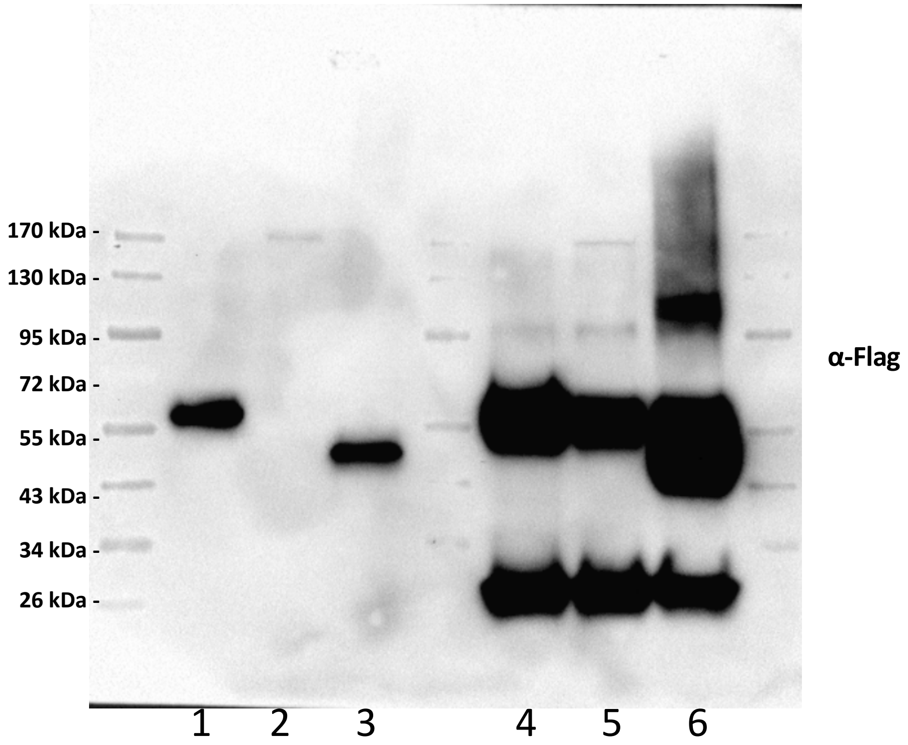
170 kDa -
130 kDa -
95 kDa -
72 kDa -
55 kDa -
43 kDa -
34 kDa -
26 kDa -
α-Flag
1
2
3
4
5
6

S3a

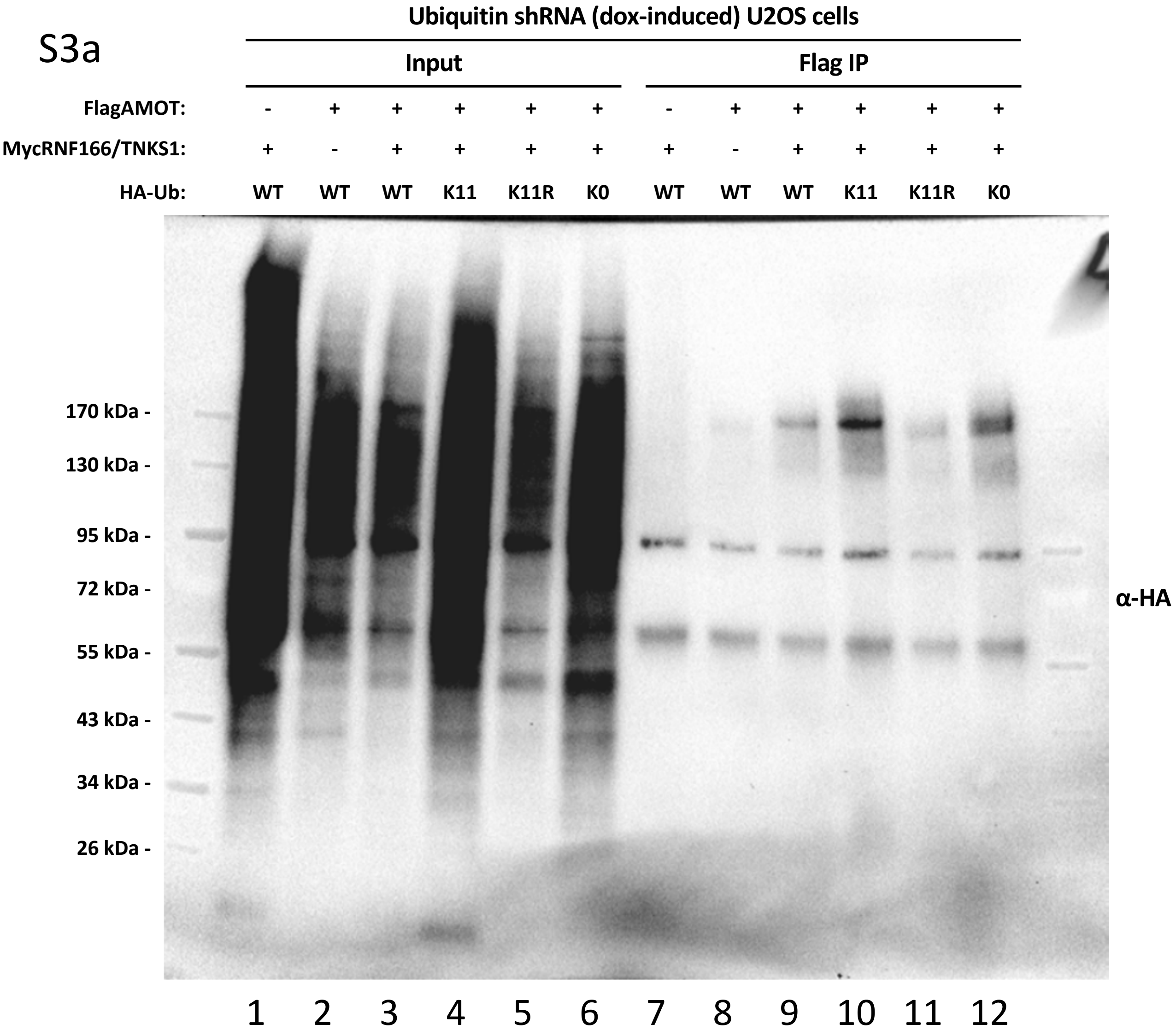

Ubiquitin shRNA (dox-induced) U2OS cells
Input
Flag IP
FlagAMOT: - + + + + + - + + + + +
MycRNF166/TNKS1: + - + + + + + - + + + +
HA-Ub: WT WT WT K11 K11R K0 WT WT WT K11 K11R K0
170 kDa -
130 kDa -
95 kDa -
72 kDa -
55 kDa -
43 kDa -
34 kDa -
26 kDa -
α-HA
1 2 3 4 5 6 7 8 9 10 11 12

S3a

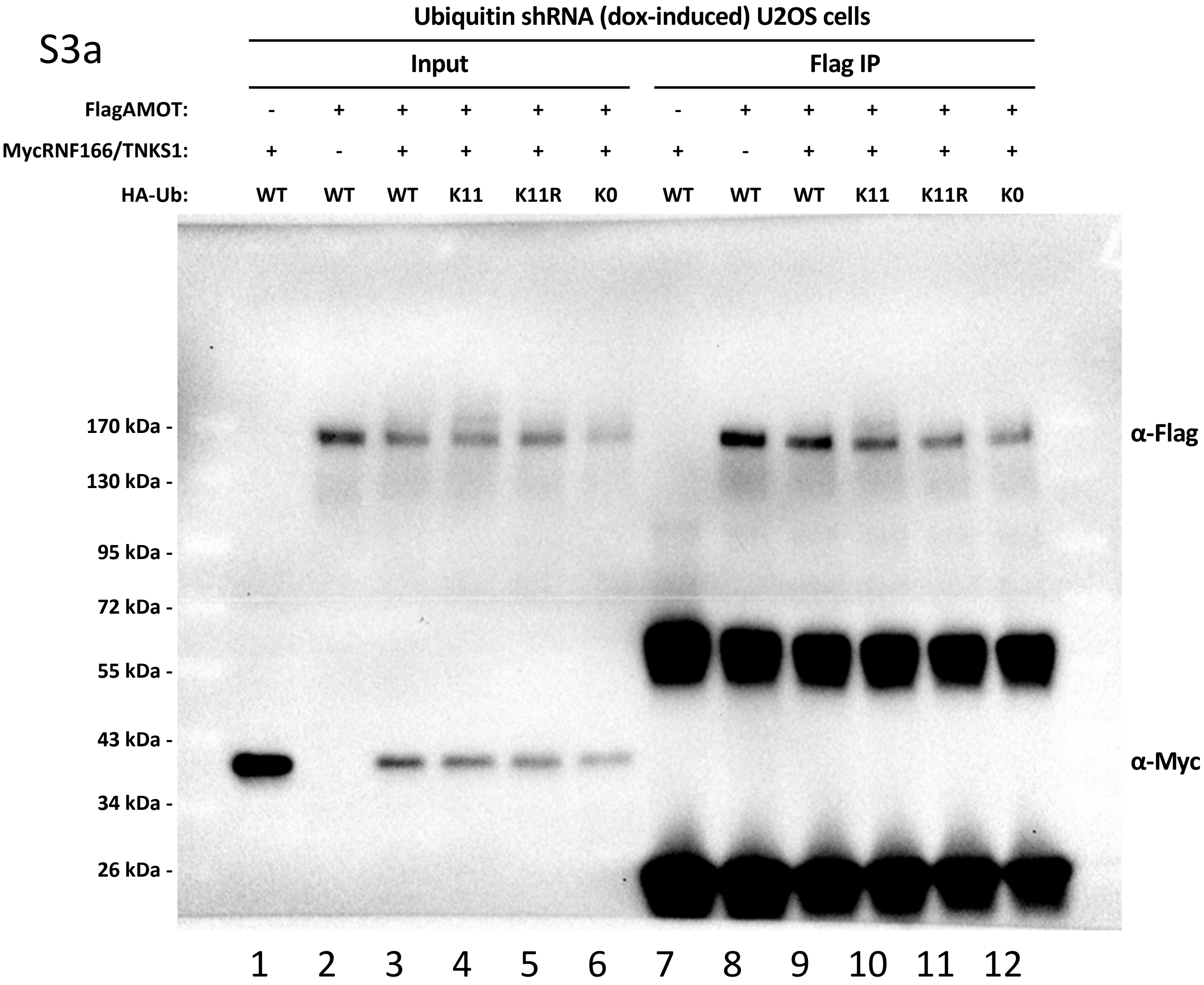
Ubiquitin shRNA (dox-induced) U2OS cells
Input
Flag IP
FlagAMOT: - + + + + + - + + + + +
MycRNF166/TNKS1: + - + + + + + - + + + +
HA-Ub: WT WT WT K11 K11R K0 WT WT WT K11 K11R K0
170 kDa -
130 kDa -
95 kDa -
72 kDa -
55 kDa -
43 kDa -
34 kDa -
26 kDa -
α-Flag
α-Myc
1 2 3 4 5 6 7 8 9 10 11 12
